# Metabolic modeling identifies determinants of thermal growth responses in *Arabidopsis thaliana*

**DOI:** 10.1111/nph.20420

**Published:** 2025-01-24

**Authors:** Philipp Wendering, Gregory M. Andreou, Roosa A. E. Laitinen, Zoran Nikoloski

**Affiliations:** ^1^ Bioinformatics Department Institute of Biochemistry and Biology, University of Potsdam Karl‐Liebknecht‐Str. 24‐25 Potsdam 14476 Germany; ^2^ Systems Biology and Mathematical Modeling Group Max Planck Institute of Molecular Plant Physiology Am Mühlenberg 1 Potsdam 14476 Germany; ^3^ Organismal and Evolutionary Research Programme, Faculty of Biological and Environmental Sciences, Viikki Plant Science Centre University of Helsinki Viikinkaari 1 Helsinki 00790 Finland

**Keywords:** *Arabidopsis thaliana*, growth, metabolic engineering, temperature effects, temperature resilience

## Abstract

- Temperature is a critical environmental factor affecting nearly all plant processes, including growth, development, and yield. Yet, despite decades of research, we lack the ability to predict plant performance at different temperatures, limiting the development of climate‐resilient crops. Further, there is a pressing need to bridge the gap between the prediction of physiological and molecular traits to improve our understanding and manipulation of plant temperature responses.
- Here, we developed the first enzyme‐constrained model of *Arabidopsis thaliana*'s metabolism, facilitating predictions of growth‐related phenotypes at different temperatures.
- We showed that the model can be employed for *in silico* identification of genes that affect plant growth at suboptimal growth temperature. Using mutant lines, we validated the genes predicted to affect plant growth, demonstrating the potential of metabolic modeling in accurately predicting plant thermal responses.
- The temperature‐dependent enzyme‐constrained metabolic model provides a template that can be used for developing sophisticated strategies to engineer climate‐resilient crops.

## Abstract 2

See also the Commentary on this article by Wang, **247**: 5–7.

## Zusammenfassung

- Temperatur ist ein wichtiger Umweltfaktor, der sich auf fast alle pflanzlichen Prozesse, einschließlich Wachstum, Entwicklung und Ertrag, auswirkt. Trotz jahrzehntelanger Forschung sind wir jedoch nicht in der Lage, die Leistung von Pflanzen bei unterschiedlichen Temperaturen vorherzusagen, was die Entwicklung klimabeständiger Nutzpflanzen einschränkt. Darüber hinaus besteht die dringende Notwendigkeit, die Lücke zwischen der Vorhersage physiologischer und molekularer Merkmale zu schließen, um unser Verständnis und die Möglichkeiten der Manipulation pflanzlicher Reaktionen auf Temperaturänderungen zu verbessern.
- Hier haben wir das erste enzymbasierte stöchiometrische Stoffwechselmodell von *Arabidopsis thaliana* entwickelt, welches die Vorhersage wachstumsbezogener Phänotypen unter verschiedenen Temperaturen ermöglicht.
- Wir haben gezeigt, dass das Modell zur computergestützten Identifikation von Genen eingesetzt werden kann, die das Pflanzenwachstum bei suboptimalen Wachstumstemperaturen beeinflussen. Anhand von T‐DNA Insertionslinien validierten wir die Gene, die das Pflanzenwachstum beeinträchtigen, und zeigten damit das Potenzial der Stoffwechselmodellierung für die präzise Vorhersage pflanzlicher Temperaturantworten.
- Das entwickelte temperaturabhängige, enzymbasierte Stoffwechselmodell bietet eine Basis für die Entwicklung ausgefeilter Strategien zur Entwicklung klimabeständiger Nutzpflanzen.

## Introduction

Global food security depends on crop yields that are severely threatened by more fluctuating and increasing temperatures – a hallmark of future climate scenarios (Wheeler & von Braun, 2013). Ambient temperature affects all aspects of the plant life cycle, from development and growth to reproduction (Casal & Balasubramanian, 2019; Zhu *et al*., 2022). Plant responses to temperature changes are most immediately observed at the level of metabolism, followed by changes in gene expression to reestablish homeostasis (Casal & Balasubramanian, 2019). Considering that metabolism is tightly linked to plant growth (Meyer *et al*., 2007; Pyl *et al*., 2012), metabolic changes can facilitate rapid plant adaptation to temperature changes at a minimal growth penalty. While we understand that metabolic flexibility is achieved by rerouting nutrient flows within the plant metabolic network, we know little about (1) which enzymes limit plant metabolic changes in temperature? And (2) how these limits emerge from temperature‐dependent biochemical constraints under which the metabolic network operates? The availability of a mathematical model that can accurately predict genetic and molecular determinants that affect plant temperature responses will address both questions.

A few metabolic models have already considered the effect of temperature on processes that directly affect plant growth (Clark *et al*., 2020; Wendering & Nikoloski, 2023). For instance, the classical mathematical model of C_3_ photosynthesis (Farquhar *et al*., 1980) – an indispensable metabolic pathway for photoautotrophic growth – has been extended to predict effects of temperature changes in net CO_2_ assimilation (Scafaro *et al*., 2023). However, this and other modeling efforts addressing responses of metabolic pathways to temperature change (Kannan *et al*., 2019; Herrmann *et al*., 2020; Inoue & Noguchi, 2021) consider only a few, lumped metabolic reactions. As a result, these models cannot be used to identify all gene targets modulating plant thermal responses, thus restricting their capacity to predict mitigation strategies. In addition, they cannot be used to make predictions about plant growth responses, due to the limited focus on one selected metabolic pathway. By contrast, genome‐scale metabolic models, representing the entirety of known metabolic reactions in a system, have been successfully used to predict growth‐related phenotypes and genetic engineering strategies for their modulation using approaches from the constraint‐based modeling framework (Herrmann *et al*., 2019; Tong *et al*., 2023; Wendering & Nikoloski, 2023). These models allow the design of rational engineering strategies to modulate metabolic phenotypes, including growth (Küken & Nikoloski, 2019). Temperature effects have already been considered in genome‐scale metabolic models of *Escherichia coli* (Chang *et al*., 2013) and *Saccharomyces cerevisiae* (Li *et al*., 2021); however, these studies either focused on a relatively narrow temperature range (Chang *et al*., 2013) or required additional parameter tuning to reproduce growth rates at superoptimal growth temperatures (Li *et al*., 2021).

Here, we present the first plant metabolic model that, by capturing temperature effects on enzyme properties and photosynthesis‐related parameters, can accurately predict growth of *Arabidopsis thaliana* at different temperatures. Due to the fine‐grained representation of metabolism, our model can correctly identify genes affecting temperature‐dependent growth in *A. thaliana*. Due to the enzyme‐constrained formulation of the model, the prediction of growth is also accompanied by predictions of reaction fluxes and enzyme abundances. Our contribution also facilitates the identification of temperature‐specific growth‐limiting metabolites and proteins, pointing to additional ways to improve plant temperature resilience. Therefore, our study provides a novel direction for engineering temperature‐resilient plants for future climate scenarios.

## Materials and Methods

### Refinement and extensions to the Arabidopsis core model

To simulate temperature‐dependent plant growth and reaction fluxes, all constraint‐based simulations were carried out using a refined version of the AraCore model (Arnold & Nikoloski, 2014), which captures the reaction stoichiometries and gene associations in the primary metabolism of *Arabidopsis thaliana* (L.) Heynh. Reading the AraCore model using the COBRA toolbox function *readCbModel* resulted in six gene artifacts for the stoichiometries of protein complexes; these were removed from the model. Additionally, refinements of the AraCore model from previous publications were considered (Yuan *et al*., 2016a; Blätke & Bräutigam, 2019; von Bismarck *et al*., 2023). The biomass reaction was further updated, such that the substrate mass fractions add up to 1 g g^−1^ dry weight (DW). Finally, the biomass reaction (Bio_opt) was reformulated to include pseudo‐metabolites for protein, DNA, RNA, carbohydrate, and lipid, which draw appropriate metabolites from the common pool with known molar fractions in biomass. The pseudometabolites enter the biomass reaction with coefficients of −1. The final refined model (aracore v.2.1) is publicly available at https://github.com/pwendering/ArabidopsisCoreModel.

To enable the investigation of effects on enzyme kinetics and protein stability, the AraCore model was extended to an enzyme‐constraint model, termed ecAraCore, using the gecko toolbox v.2.0.2 (Sánchez *et al*., 2017). Here, the flux of a reaction is limited by the product of enzyme abundance $\left[E\right]$ and turnover number ($k_{\mathrm{cat}}$):

(Eqn 1) $$v\leq \left[E\right]\cdot k_{\mathrm{cat}}$$

Further, the sum of enzyme abundances is limited by a fraction of the total protein content, $P_{\mathrm{tot}}$:

(Eqn 2) $$\sum_{i}\left[E_{i}\right]\cdot {\mathrm{MW}}_{i}\leq P_{\mathrm{tot}}\cdot f\cdot \sigma$$

The fraction of the total protein content is determined by the factors f and σ, representing the coverage of all proteins of the organism by the model and the average enzyme saturation, respectively; MW denotes the molecular weight of the respective enzyme. Consdering that the integration of f and σ led to unrealistically small predictions of relative growth rate, both were set to one.

### Constraint‐based modeling based on the refined AraCore model

To predict temperature‐dependent relative growth rates (RGRs), we relied on the flux through the light‐limited biomass reaction (Bio_opt) in the ecAraCore model, subject to constraints on metabolic steady state and the developed temperature‐dependent constraints. From this optimization problem, the maximum RGR was obtained. Under the assumption that plants allocate their resources parsimoniously, we further minimized the sum of fluxes in a second step while guaranteeing for near‐optimum RGR. All presented values for predicted net CO_2_ assimilation rates (A) and reaction flux distributions (v) originate from this second optimization step. All predictions were carried out with temperatures between 10°C and 40°C, which is a range that *A. thaliana* typically experiences in nature (Casal & Balasubramanian, 2019). To explore the solution space of the temperature‐dependent ecAraCore model, flux variability analysis and flux sampling were performed. The constraint‐based optimization procedures are further detailed in Supporting Information Methods S1 and Figs S1–S2.

### Introduction of temperature dependences into the constraint‐based model

The effects of temperature on metabolic fluxes were considered its impacts on: (1) enzyme kinetics; (2) the total protein content; and (3) photosynthesis. Briefly, the effects of temperature on enzyme kinetics ($k_{\mathrm{cat}}$ values) were incorporated by assuming that the catalytic optimum of the enzyme is equal to its stability optimum and that its catalytic rate is limited by the availability of native enzyme at high temperatures. Following these assumptions, a temperature model for enzyme kinetic rates (Hobbs *et al*., 2013) was fitted for each $k_{\mathrm{cat}}$ value in the model (see ‘Temperature adjustment of turnover numbers’ in the Materials and Methods section for more details). Further, experimental data on the total protein content ($P_{\mathrm{tot}}$ ) for *A. thaliana* Col‐0 at different temperatures were obtained from the literature to derive an empirical model (see ‘Temperature dependence of total protein content’ in the Materials and Methods section for more details). The Eqns 1 and 2 change accordingly:

(Eqn 3) $$v\leq \left[E\right]\cdot k_{\mathrm{cat}}\left(T\right)$$

(Eqn 4) $$\sum_{i}\left[E_{i}\right]\cdot {\mathrm{MW}}_{i}\leq P_{\mathrm{tot}}\left(T\right)\cdot f\cdot \sigma$$

The temperature dependences on photosynthesis and CO_2_ uptake were introduced by considering the temperature dependences of the individual parameters as listed in Table S1. The conversion between area and mass units was done using temperature‐dependent leaf mass per area (Table S2; Fig. S3).

### Inference of key temperatures from TPP data

To determine the key temperatures of proteins, cross‐species thermal proteome profiling (TPP) data were first downloaded from the Meltome Atlas (Jarząb *et al*., 2020) and filtered for ‘*Arabidopsis thaliana* seedling lysate’. The relative abundances at the respective temperatures were fitted to the *beta growth function* (Yin *et al*., 2003), which was mirrored at the inflection point to model a sigmoidal decay:

(Eqn 5) $$f_{N}\left(T\right)=a\left(1+\frac{b+T}{b-c}\right){\left(-\frac{T}{b}\right)}^{\frac{b}{b-c}}$$

The function $f_{N}\left(T\right)$ describes the fraction of nondenatured (native) protein at temperature T. The parameters a, b, and c were estimated using the MATLAB (MATLAB, 2020) *fit* function. The parameter b is the temperature optimum ($T_{\mathrm{opt}}$); the melting temperature was determined as the larger root of $g\left(T\right)=f_{N}\left(T\right)-0.5a$. A threshold of $R_{\mathrm{adj}}^{2}\geq 0.6$ was applied to filter out fits of insufficient quality (Fig. S4c,d). As a result, key temperatures were matched to 53% of the proteins in the ecAraCore model.

### Prediction of missing $T_{\mathrm{opt}}$ values

To obtain optimal temperatures for the remaining 47% of the proteins in the metabolic model, a Random Forest regression model was trained. The model predicts $T_{\mathrm{opt}}$ based on amino acid sequence features. To this end, $T_{\mathrm{opt}}$ for all proteins in the Meltome Atlas were obtained using the *beta growth function*, and amino acid sequences were downloaded from UniProt (Bateman *et al*., 2021). After feature selection, the final model was trained with 69 amino acid sequence features to predict $T_{\mathrm{opt}}$ values. A list of all considered features can be found in Dataset S1. This model exhibited an adjusted $R^{2}$ value of 0.62 and an RMSE of 6.83°C from fivefold cross‐validation. The Random Forest regression model is available via a command line interface tool (https://github.com/pwendering/topt‐predict). In its current state, the tool further allows for automated extraction of amino acid features for given amino acid sequences, which are then standardized using the distribution properties of the training set that was used to train the Random Forest regressor. Additional details can be found in Methods S1.

### Temperature adjustment of turnover numbers

To model the temperature dependence of enzyme‐catalyzed reaction rates, macromolecular rate theory (MMRT) was proposed, which considers the change in heat capacity explicitly in the Eyring equation (Hobbs *et al*., 2013):

(Eqn 6) $$\begin{array}{rcl} \log_{e}\left(k\right) & = & \text{MMRT}\left(T\right)\\ & = & \log_{e}\left(\frac{k_{B}T}{h}\right)-\frac{\Delta H_{T_{0}}^{‡}+\Delta C_{p}^{‡}\left(T-T_{0}\right)}{\mathrm{RT}}\\ & & +\;\frac{\Delta S_{T_{0}}^{‡}+\Delta C_{p}^{‡}\log_{e}\left(T/T_{0}\right)}{R} \end{array}$$

with $k_{B}$ denoting the Boltzmann constant, h, the Planck's constant, R, the universal gas constant, and $T_{0}$ the reference temperature (assumed to be 293.15 K).

The parameters $\Delta H_{T_{0}}^{‡}$ (enthalpy change), $\Delta S_{T_{0}}^{‡}$ (entropy change), and $\Delta C_{p}^{‡}$ (heat capacity change) were estimated using a system of three equations:

(Eqn 7) $$\text{MMRT}{\left(T\right)}_{|T=T_{\mathrm{opt}}}=\log_{e}\left(k_{\mathrm{cat}}^{\max}\right)$$

(Eqn 8) $${\text{MMRT}}^{\prime }{\left(T\right)}_{|T=T_{\mathrm{opt}}}=0$$

(Eqn 9) $$\text{MMRT}{\left(T\right)}_{|T=T_{H}}=\log_{e}\left(f_{N}\left(T_{H}\right)\cdot k_{\mathrm{cat}}^{\max}\right)$$

Eqn 7 ensures that the maximum measured $k_{\mathrm{cat}}$ value is reached at the optimal temperature and Eqn 8 sets the optimum of the function to be at $T_{\mathrm{opt}}$ by equating the first derivative of $\text{MMRT}\left(T\right)$ at $T_{\mathrm{opt}}$ with zero. The third equation in the system, Eqn 9, sets the value of the $k_{\mathrm{cat}}$ at $T_{H}$ to the associated fraction of native protein multiplied by the maximum $k_{\mathrm{cat}}$. For proteins with available fits to TPP data, $T_{H}$ was set to the maximum experimental temperature of 70.4°C and $f_{N}\left(T_{H}\right)$ is the native fraction read out from the function fit. For the remaining proteins, $T_{H}$ was set to 100°C, with $f_{N}\left(T_{H}\right)=10^{-6}$, assuming almost complete unfolding at 100°C. The three thermodynamic parameters were estimated in a protein‐ and reaction‐specific manner, that is for every protein and reaction catalyzed by the protein, three parameters were estimated depending on the respective $k_{\mathrm{cat}}$. The distribution of the resulting parameter estimates is shown in Fig. S5.

### Temperature dependence of total protein content

To determine the temperature dependence of protein content, experimental measurements of the total protein content of *A. thaliana* Col‐0 were collected from 18 studies (25 data points, Dataset S2) with adult plants grown at irradiances between 100 and 465 μmol m^−2^ s^−1^ as well as with photoperiods between 8 and 12 h. The sampling time ranged from 18 d after germination (DAG) to 42 d after sowing (DAS). If sampling was performed multiple times per day, the latest time point was selected. In total, seven different functions were fitted to the dataset, including a linear function, three polynomial functions, a sigmoid function, the *beta growth function* (Yin *et al*., 2003), and a function similar to the probability density function of the gamma distribution (bcdf) (Fig. S6). The best fits with respect to RMSE were obtained for the cubic, the *beta growth function*, and the bcdf. For temperature‐dependent modeling, the bcdf was selected, as it prevents the modeled protein content from approaching zero too far below lethal temperatures.

### Robustness and sensitivity analyses

To assess the robustness of the model's predictions to randomly introduced uncertainties in its parameters, 1000 parameter samples were used to predict temperature responses in RGR and A. To this end, we assumed a SD (σ) of 5% of the mean value (μ) for each of the considered parameters. Moreover, we assumed that the parameters are log‐normally distributed with the following parameters:

(Eqn 10) $$\mu_{\log}=\log\mu$$

(Eqn 11) $$\sigma_{\log}=\sqrt{\log_{e}\left(1+\frac{\sigma^{2}}{\mu^{2}}\right)}$$

Like this, parameter distributions for $V_{\text{cmax}},K_{c},K_{o},k_{c},k_{o}$, and $J_{\max}$ were generated and transformed back to linear scale to use them in the temperature‐dependent constraints.

The sensitivity of the model to individual parameter changes was investigated by increasing and decreasing the values of $V_{\text{cmax}},K_{c},K_{o},k_{c},k_{o}$, ϕ, $J_{\max}$, $g_{s}$, and $g_{m}$ by 10% and rerunning the predictions of RGR and A at 10°C, 25°C, and 40°C.

The parameters included in the sensitivity and robustness analyses are important parameters of the FvCB model (Farquhar *et al*., 1980) and extensions thereof (Farquhar & Wong, 1984; Niinemets *et al*., 2009), which were considered in the updated model constraints. Explanations of the parameters are given in Table S1.

To further assess the sensitivity of the model to different values of the ratio between chloroplastic CO_2_ and O_2_ concentrations, the RGR responses and pairwise rank correlations between predictions with different ratios have been computed (Methods S1; Fig. S7).

### Reaction flexibility index

To describe the flexibility of reaction fluxes at a given temperature irrespective of the magnitude of its median flux, we defined a measure, which we termed reaction flexibility index (RFI). The RFI for reaction i at temperature j is defined by the quotient of the interquartile range (*iqr*) and the median of sampled fluxes for this reaction at the specified temperature:

(Eqn 12) $${\mathrm{RFI}}_{i,j}=\frac{\mathrm{iqr}\left(v_{i,j}\right)}{{\tilde{v}}_{i,j}}$$

### Identification of limiting metabolites

Limiting metabolites at different temperatures (10°C to 40°C in 5°C steps) were determined by adding import reactions for each metabolite in the ecAraCore model individually, with an upper limit of 1 mmol gDW^−1^ h^−1^. This limit was arbitrarily chosen and does not correspond to any assumed external concentration. Moreover, the ratio between nitrate and ammonia import reactions was fixed to 3 : 1 (M'rah Helali *et al*., 2010). The resulting relative growth rate from each simulated metabolite supply was recorded and the increase with respect to the default model was calculated. For a better comparison of the sets of metabolites found at each temperature, the growth increases were scaled to the maximum per temperature. A threshold of 0.1 on the scaled growth increases and a threshold of 1% on the unscaled relative increase were then used to filter out irrelevant metabolites.

Further, the growth responses were clustered using K‐medoids clustering (1‐cosine similarity as distance measure). The input for the clustering was the relative increase in relative growth rate. Metabolites with an increase below 1% at all temperature were excluded. As for the median flux sums, the optimal number of clusters, K, was determined by choosing K, which corresponds to the maximum median Silhouette Index (Fig. S8). For a light intensity of 150 μmol m^−2^ s^−1^, *K* = 11 was chosen and *K* = 8 was chosen with *I* = 400 μmol m^−2^ s^−1^. An ambient partial pressure of $p\left(CO_{2}\right)=380\;\text{μmol}$ was used for all simulations.

### Identification of limiting proteins

To identify proteins that are limiting growth at different temperatures (10°C to 40°C in 5°C steps), the temperature adjustment of $k_{\mathrm{cat}}$ was removed for each protein one at a time. This was performed to mimic the replacement of the protein by a thermostable version. Each of the resulting optimization problems was then solved to maximize the flux through the biomass reaction (i.e. RGR). To be able to compare potential engineering targets across all temperatures, the increase in the RGR of *in silico* engineered lines compared with the RGR of the wild‐type (WT) was scaled by the maximum increase observed per temperature. A threshold of 0.05 on the scaled growth increases and a threshold of 0.01% on the unscaled relative increase were then used to filter out irrelevant proteins. A light intensity of 400 μmol m^−2^ s^−1^ and $p\left(CO_{2}\right)=380\;\text{μmol}$ were used for all simulations.

### Prediction of thermosensitive knockout mutants

To predict how relative growth rate changes with temperature, knockouts at the reaction level were simulated by setting the upper bound of each reaction to zero that carried flux in the solution of the WT model. A light intensity of 150 μmol m^−2^ s^−1^ and $p\left(CO_{2}\right)=380\;\text{μmol}$ were used for all simulations. The predicted RGRs for WT and mutants were then compared by the fold changes between the RGR of the WT by the RGR of the mutant. Considering that the effect of knocking out a gene cannot fully be predicted in case of functional redundancy, we focused on qualitative results, considering all knockouts with a reduction of 1% or more as having a reduced relative growth rate. Knockouts with no effect were determined by identifying *in silico* knockouts that showed > 99% of the WT RGR across all tested temperatures (17°C, 25°C, 27°C, 35°C, 45°C).

### Plant growth conditions and temperature shift

To experimentally validate the predictions, *A. thaliana* T‐DNA insertion lines were obtained from the Nottingham Arabidopsis Stock Center (NASC) (Table S3). Before seed sowing, seeds were stratified over 5 d at 4°C in the dark in a 0.1% agarose solution. Each line was then sown in a 2 : 1 (peat : vermiculite) soil mixture and germinated for 10 d in growth rooms under long‐day (LD) conditions, 16 h : 8 h, 23°C : 19°C, light : dark with 240 μmol m^−2^ s^−1^ light radiation and 50–60% humidity. Each line was grown in four replicates in 7‐cm pots, each replicate pot containing four plants. After 10 d, all pots were moved to a controlled growth chamber to constant 17°C LD conditions. To minimize error due to the growth chamber, the trays were moved and rotated every second day. Once the plants had 6–8 true leaves, leaf rosettes from each pot were cut and weighed for fresh weight. The rosettes were dried in an oven set to 65°C for 3 d and immediately reweighed to provide dry weight measurements. The plants were grown in four batches, each containing the Col‐0 WT and partially overlapping sets of T‐DNA insertion lines.

### Statistical analysis of dry weight measurements

To test for statistical differences in final dry weight between the WT and the T‐DNA insertion lines, linear mixed‐effect models were used to accommodate for the effect of the four different batches. The models were created using the R (v.4.1.2) package lme4 (v.1.1–32, functions *lmer* and *lme*). In both models, the batch number was set as random effect. To test for significant differences between WT and T‐DNA insertion lines, *post hoc* test procedures from the packages emmeans (v.1.8.8, function *emmeans*) and multcomp (v.1.4–25, function *glht*) were applied to each of the two linear mixed‐effect models both with and without Benjamini–Hochberg correction for multiple hypothesis testing. In the statistical analysis, we only considered the genotypes for which at least three replicates were measured.

## Results

### Integrating temperature‐dependent constraints in a model of *A. thaliana* metabolism

**Fig. 1:**
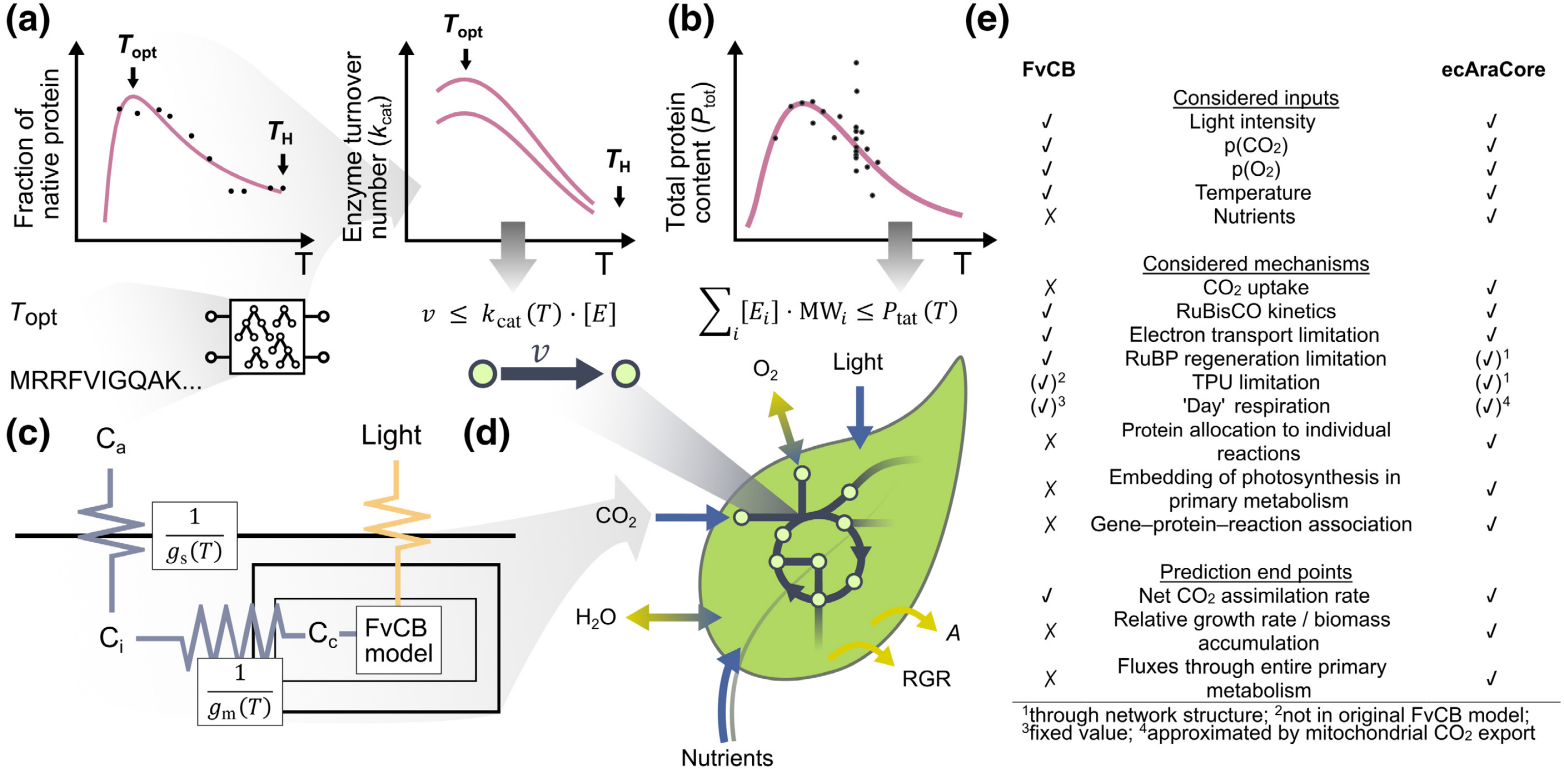
Temperature‐dependent model of *Arabidopsis thaliana*'s central metabolism. Experimental data on protein thermostability and enzyme kinetics, total protein content ($P_{\mathrm{tot}}$), and CO_2_ uptake along with prediction from the classical C_3_ photosynthesis model were used to derive temperature‐dependent constraints that were integrated into a metabolic model of *A. thaliana*'s central metabolism. (a) Key temperatures of protein thermostability (i.e. optimal temperature, $T_{\mathrm{opt}}$, and heat denaturation temperature, $T_{H}$) were inferred from thermal protein profiling data (Jarząb *et al*., 2020); for proteins for which such data were not available, $T_{\mathrm{opt}}$ was predicted using a Random Forest regression model trained on features derived from amino acid sequences, as indicated by the scheme below the line graph (Supporting Information Dataset S1). Protein key temperatures were used to model temperature‐dependent, enzyme‐specific $k_{\mathrm{cat}}$ values (cf Materials and Methods section). (b) Temperature dependence of $P_{\mathrm{tot}}$ was described by a gamma‐distribution‐like function (Dataset S2). (c) The Farquhar, von Caemmerer, and Berry model was parametrized for *A. thaliana* Col‐0 considering temperature‐dependent parameters. The uptake of CO_2_ from air to the carboxylation site of RuBisCO was considered by including temperature‐dependent stomatal conductance ($g_{s}$) and mesophyll conductance ($g_{m}$). (d) Enzyme‐constrained metabolic model that integrates light intensity as well as ambient partial pressures of CO_2_ (modeled by $g_{s}$ and $g_{m}$) and O_2_ as additional input parameters, and allows the prediction of relative growth rate and net CO_2_ assimilation rate, A, along with reaction fluxes, v, and abundances of enzymes, $\left[E\right],$ with molecular weight MW. (e) Comparison of the FvCB model and the ecAraCore model with respect to the considered inputs and mechanisms, and the traits that can be predicted. TPU: triose phosphate utilization.

To develop an accurate model that allows us to predict metabolic phenotypes of *A. thaliana* grown at different temperatures, we made use of available data for the Columbia‐0 (Col‐0) accession. To this end, the model considered the temperature dependence of: (1) enzyme catalytic rates; (2) total protein content; and (3) photosynthesis (Fig. 1). First, to describe the temperature dependence of enzyme catalytic rates, we required access to key temperatures of protein thermostability (i.e. optimal temperature, $T_{\mathrm{opt}}$, and heat denaturation temperature, $T_{H}$). We determined these key temperatures from available thermal protein profiling (TPP) data (Jarząb *et al*., 2020) (Figs 1a, S4). To predict $T_{\mathrm{opt}}$ of *A. thaliana* proteins for which no TPP data were available, we trained a Random Forest regression model using features derived from amino acid sequences of 12 species (cf Materials and Methods section, Methods S1). The resulting model used 69 features (Dataset S1) and matched the performance of other published efforts (adjusted $R^{2}$ value of 0.62 and an RMSE of 6.83°C from fivefold cross‐validation, Table S4) (Li *et al*., 2022; Yang *et al*., 2022). Reaction‐specific turnover numbers ($k_{\mathrm{cat}}$) of enzymes in the model were obtained from the BRENDA database (Chang *et al*., 2021) using well‐established procedures (Domenzain *et al*., 2022). We then fitted the macromolecular rate theory model (Hobbs *et al*., 2013) to describe the temperature dependence of $k_{\mathrm{cat}}$ values (Figs S4e, S5; Materials and Methods section). Second, we determined the temperature dependence of the total protein content by fitting experimental data from 16 studies (Figs 1b, S6; Dataset S2). Third, to model the temperature dependence of photosynthesis, we employed the C_3_‐photosynthesis model from Farquhar, von Caemmerer, and Berry (FvCB model, Farquhar *et al*., 1980) to introduce constraints on the net CO_2_ assimilation rate (A), the ratio between the oxygenation and carboxylation reactions catalyzed by the RuBisCO enzyme (ϕ), as well as the light (i.e. electron transport) and CO_2_ uptake limitations to A (Fig. 1c; Methods S1). We also modeled the relationship between ambient and chloroplastic CO_2_ partial pressure by including the effects of stomatal conductance, $g_{s},$ and mesophyll conductance, $g_{m}$. The FvCB model was parametrized using experimental data specific for Col‐0 whenever possible, considering the temperature dependences of the 12 model parameters (Table S1; Figs S3, S9–S11).

The resulting temperature constraints were integrated into a refined model of *A. thaliana* central metabolism (aracore v.2.1, Fig. 1d) (Arnold & Nikoloski, 2014). This metabolic model comprises 415 metabolites, involved in 585 reactions associated with 706 genes. We also introduced enzyme constraints, whereby each reaction flux is limited by the product of an enzyme‐ and reaction‐specific $k_{\mathrm{cat}}$ and the abundances of the considered enzymes (Domenzain *et al*., 2022). Further, the sum of all enzyme contents was bounded by the total protein content ($P_{\mathrm{tot}}$). This enzyme‐constrained model, termed ecAraCore, contains 2507 variables and 1335 constraints arising from the enzyme mass balance constraints added for 671 proteins included in the model. The ecAraCore model was extended by three additional constraints derived from the FvCB model. As a result, the ecAraCore model allows the usage of ambient temperature, light intensity, CO_2_ and O_2_ partial pressures as input to predict protein abundances, reaction fluxes, the RGR, and the net CO_2_ assimilation rate, A. It therefore extends the predictive ability of the FvCB model by considering the uptake of CO_2_, nutrient assimilation, the embedding of photosynthesis in the network of primary metabolism, the allocation of protein to individual reactions, and the association of reactions to catalyzing enzymes and their encoding genes (Fig. 1e). In addition to the prediction of the net CO_2_ assimilation rate, it extends the FvCB model further by allowing the prediction of RGR and fluxes through individual reactions in central metabolism as well as genetic interventions that go beyond those of single enzyme (i.e. RuBisCO).

### Prediction of thermal responses of growth and the net CO_2_ assimilation rate in *A. thaliana*

**Fig. 2:**
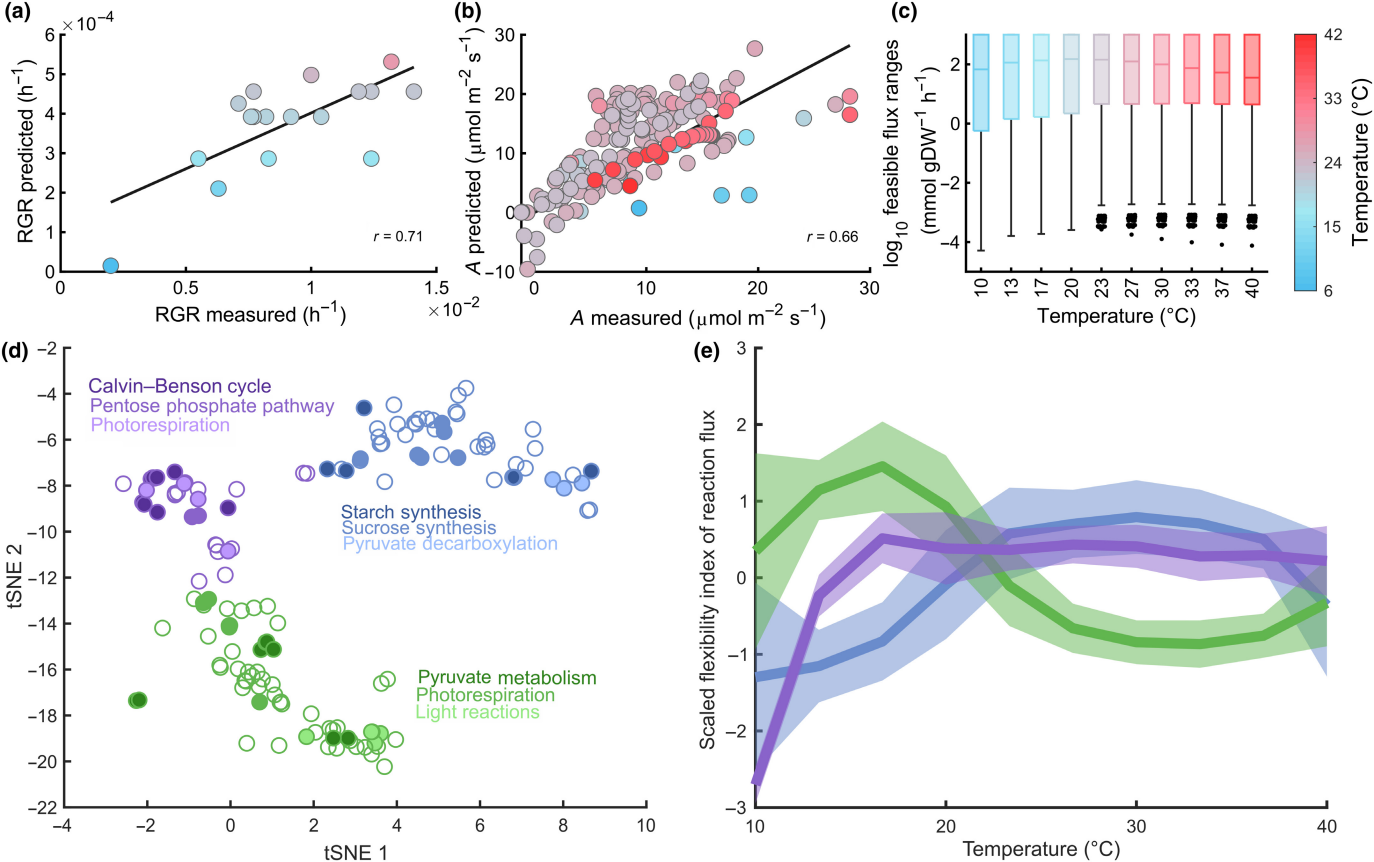
Predictions of growth‐related traits in *Arabidopsis thaliana* at different temperatures. Comparison of predicted (a) relative growth rate (RGR) at light intensity of $150\;\text{μmol}\;m^{-2}s^{-1}$ and (b) net CO_2_ assimilation rate, A, to experimental data for *A. thaliana* at different temperatures (Supporting Information Datasets S3 and S4). The Pearson's correlation coefficient is denoted by r. The line in (a) depicts a linear univariate regression model fitted to the data points. The line in (b) indicates perfect agreement between experimental and predicted data. (c) Distribution of feasible flux ranges at different temperatures, as obtained by flux variability analysis without constraining the RGR. Box plots show the interquartile range (IQR), the middle line represents the median, and vertical lines represent whiskers that either extend to 1.5 times the IQR or the minimum or maximum value, respectively. gDW, gram dry weight. (d) Two‐dimensional representation of plasticity of reaction fluxes to temperature obtained by using t‐SNE (Maaten & Hinton, 2008). The flexibility index of a reaction flux at a given temperature was determined by the quotient of the interquartile range and the median of sampled reaction fluxes. These were obtained by flux sampling (*n* = 30 000) at 90% of the optimal RGR and minimum total flux through the network, obtained by parsimonious flux balance analysis. The coordinates obtained from t‐SNE were subject to K‐medoids clustering. The resulting clusters (*K* = 3) are color‐coded. The three most abundant pathways per cluster are encoded by different shades of the respective cluster color. (e) Median of *z*‐scaled values for the flexibility index values for the three clusters shown in (d). The shaded area represents the SD of the *z*‐scaled values of the flexibility index in the clusters.

We next used the developed ecAraCore model to predict the steady‐state net CO_2_ assimilation rate and RGR (i.e. the rate of accumulation of new dry mass per unit of existing dry mass), for a plant growing at temperatures between 10°C and 40°C–a range that *A. thaliana* experiences in nature (Casal & Balasubramanian, 2019). This was performed using parsimonious flux balance analysis (Lewis *et al*., 2010) (Fig. 2a,b). To assess the accuracy of predicted temperature‐dependent RGR, we compiled a dataset including 13 studies in which RGR of Col‐0 was measured for plants grown at temperatures ranging from 6°C to 28°C (Dataset S3). We found that the predicted response of RGR to increasing temperature agreed qualitatively with experimental data (Fig. 2a, Pearson r=0.71, $P=9.3\times 10^{-4}$). In addition, increasing temperature under the optimum for A (predicted at 30.2°C) led to improved RGR, in line with experimental observations in Col‐0 (Casal & Balasubramanian, 2019).

Considering that RGR is determined by the partitioning of carbon fixed by photosynthesis, we further tested the performance of the model in predicting the net CO_2_ assimilation rate, A. To this end, we assembled a dataset comprising 175 measurements of A from 21 studies performed in a range of growth conditions, covering temperatures from 7°C to 42°C. The most varying factors within the dataset included the following: the light intensity (coefficient of variation, CV = 0.74), O_2_ partial pressure (CV = 0.85), and CO_2_ partial pressure (ambient: CV = 0.56, intercellular: CV = 0.58). These factors also showed the highest Pearson's correlation coefficient to A, with r=0.39 ($P=6.7\times 10^{-8}$) for the light intensity, r=−0.37 (P=0.01) for O_2_ partial pressure, and r=0.35 ($P=2.9\times 10^{-6}$) and r=0.34 ($P=5.7\times 10^{-6}$) for ambient and intercellular CO_2_ partial pressure, respectively. We then used the data about temperature, light intensity, ambient CO_2_ partial pressure, and ambient O_2_ partial pressure as input for the FvCB and ecAraCore model to predict A and compared it with the available measurements. For both models, we found agreement with experimental data for the FvCB model (Fig. S12, r=0.70, $P=3.9\times 10^{-22}$, median absolute deviation, MAD = 4.7) and the ecAraCore model (Fig. 2b, $r=0.66,P=2.7\times 10^{-9}$, MAD = 3.9) (Dataset S4). In addition, the ecAraCore model predicted the effect of high temperature (*T* > 30°C) more accurately than the FvCB model, as quantified by the difference in MAD values ($P=3.8\times 10^{-3}$, left‐tailed Wilcoxon rank sum test, Fig. S12c,d). However, both models tended to overestimate the effect of high ambient CO_2_ partial pressure (>600μbar) and light intensity ($>1000\;\text{μmol}\;m^{-2}s^{-1}$) on A, with MAD values of $6.3\;\text{μmol}\;m^{-2}s^{-1}$ for the ecAraCore model and $7.4\;\text{μmol}\;m^{-2}s^{-1}$ for the FvCB model (Fig. S12e–h). This extensive testing with unseen data (i.e. data that were not used in the generation of the model, Datasets S3 and S4) demonstrated that the ecAraCore model can predict growth‐related traits and can therefore be used to investigate metabolic determinants of thermal responses in *A. thaliana*.

To assess the influence of parameter uncertainties on the temperature‐dependent predictions of RGR and A, we performed sensitivity analyses by: sampling random parameter configurations within an assumed standard variation of 5% around the mean values that were used in the model; and increasing and decreasing individual parameter values by 10% and quantifying the resulting change in RGR and A (see Materials and Methods section). In these analyses, changes in $V_{\text{cmax}},K_{c},K_{o},k_{c},k_{o}$, and $J_{\max}$ were considered. In the second analysis, additionally, $g_{m}$, $g_{s}$, and ϕ were included. The results of the first sensitivity analysis showed that the model's predictions were robust to parameter uncertainties, where RGR showed smaller deviations from the mean than A (Fig. S13). Among the tested parameters, the $k_{\mathrm{cat}}$ values of RuBisCO for O_2_ and CO_2_ had the greatest impact on the predictions under the tested conditions (Fig. S14). Further, $J_{\max}$ affected the predictions when temperatures of 25°C or higher were simulated. Notably, the predictions were performed at with a light intensity of $150\;\text{μmol}\;m^{-2}\;s^{-1}$ and $C_{a}$ of 400μbar, noting that other conditions may yield different solutions.

### Effect of metabolic flexibility on *A. thaliana*'s growth at different temperatures

The predicted growth results from a distribution of steady‐state reaction fluxes (Nikoloski *et al*., 2015). Therefore, we asked whether the thermal metabolic flexibility, that is the capacity of the network to support flux rerouting to maintain growth, changes with temperature. Here, we quantified thermal metabolic flexibility by the flux ranges of the underlying metabolic reactions. By providing the interval between minimum and maximum flux per reaction, these ranges describe the solution space of possible fluxes containing alternative optima in terms of flux distributions. We quantified both feasible and operational flux ranges. The feasible flux range for a reaction is specified by the minimum and maximum flux it obtains at steady state; a flux range is referred to as operational if it is achieved by additionally imposing a minimal RGR. To investigate the effect of temperature‐dependent constraints on steady‐state flux ranges, we first determined the feasible ranges of each reaction for temperatures between 10°C and 40°C (Fig. 2c). Although the largest median of feasible range size was observed at 20°C, below the predicted growth optimum (30.2°C), we found that the feasible range was significantly and highly correlated with RGR over the considered temperatures for 40% of reactions (Pearson's correlation coefficient, r≥0.8, P<0.05 , adjusted using Benjamini–Hochberg procedure). Further, the temperature responses of operational ranges for more than twice as many reactions (89%) were found to be correlated with the response of RGR (cf Methods S1; Fig. S15). These findings suggest that metabolic flexibility may affect RGR.

We then asked whether changes in metabolic flexibility of reactions in different pathways are coordinated. If so, thermal flexibility profiles would differ between reactions and would be more similar within a pathway than between pathways. The reaction ranges described above only provide the limits of reaction fluxes but do not consider the probability distribution of the individual reaction fluxes Therefore, we next performed uniform flux sampling (Price *et al*., 2004) at near‐optimal RGR. Based on 30 000 steady‐state flux distributions, we determined the sum of fluxes through reactions comprising the considered pathways, which we termed pathway fluxes. We found that median pathway fluxes respond similar to temperature changes as the optimal RGR (Figs S16, S17). Temperature‐dependent changes in the flexibility of individual reactions were determined based on the interquartile range of the probability distribution of sampled fluxes. The interquartile ranges were scaled by the median flux to allow a comparison irrespective of the magnitude of flux, yielding a reaction flexibility index (Eqn 12).

Equipped with these indices, we identified three clusters of reactions, with different shapes and optimal temperatures of their responses (Fig. 2d,e). The first cluster (green, Fig. 2d) consists of reactions with optimal temperatures of 17°C, largely involved in pyruvate metabolism, light reactions, and photorespiration. The second cluster (purple, Fig. 2d) comprises reactions that show an increase in the flexibility index up to 17°C, reaching a plateau thereafter; these reactions are a part of the Calvin–Benson cycle, photorespiration, and pentose phosphate pathway. In the third cluster (blue, Fig. 2d), we found starch synthesis, sucrose synthesis, and pyruvate decarboxylation to be the most represented pathways. This set of reactions showed a broad temperature optimum between 23°C and 37°C. Our results indicate that thermal responses of metabolic flexibility are not necessarily coordinated within pathways; however, they are similar within larger metabolic subsystems with different response profiles. Considering that reactions differed in their responses to temperature change, we reasoned that metabolites and proteins that limit growth are temperature‐specific.

### Identification of growth‐limiting metabolites and proteins at different temperatures

At the level of metabolism, both metabolite concentrations and enzyme efficiencies can be limiting to growth, complicating the assessment of factors that limit growth when plants are experiencing temperature change. To identify temperature‐specific limiting metabolites, we investigated the responses of predicted RGR to increased availability of individual metabolites (cf Materials and Methods section). Hence, metabolites most‐limiting to growth will result in the highest increase in RGR upon supplementation. This modeling scenario mimics plant supplementation or spraying with bioavailable nutrients as a facile strategy to mitigate negative effects of temperature changes (Calvo *et al*., 2014). Across all tested temperatures, the predicted RGR changes across metabolites were highly correlated (Fig. S18a,b). However, we identified that the most‐limiting metabolites differed between the tested temperatures (Fig. S18c–f). We observed that the increased availability of most metabolites yielded the greatest impact at 10°C, associated with the smallest RGR (Figs S19, S20; Datasets S5 and S6). Few metabolites (e.g. l‐tryptophan and bicarbonate, Dataset S6) were most limiting to growth at 40°C; by contrast, simulated supplementation of other metabolites (e.g. l‐arginine and O‐acetyl‐l‐serine) showed similar increases in RGR at both high and low temperatures. Since the considered objective for this analysis was the flux through the biomass reaction, 79% of the biomass constituents were limiting at least at one tested temperature. The remaining 21% of biomass metabolites were not limiting at any of the tested temperatures.

**Fig. 3:**
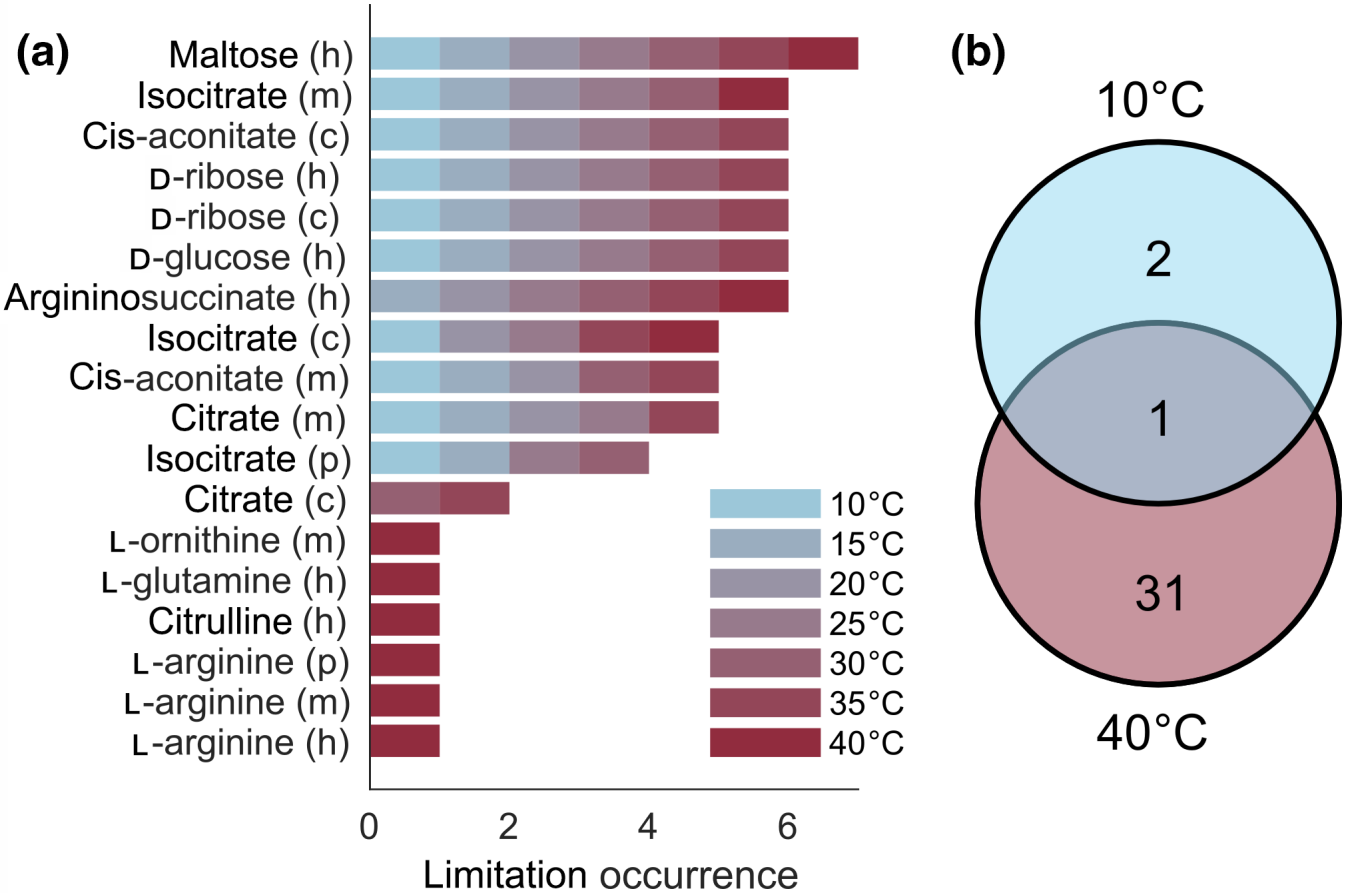
Metabolites and proteins imposing the strongest limitations on relative growth rate (RGR) in *Arabidopsis thaliana*. (a) Limiting metabolites were identified by simulating supplementation for each metabolite separately at a given temperature via an import reaction (cf Materials and Methods section; Supporting Information Dataset S6). Shown are the 10 metabolites, excluding the biomass components, ordered according to their occurrence as most limiting at seven temperatures, as indicated in the legend. The letters next to the metabolite names indicate the compartments: c, cytosol, h, chloroplast, m, mitochondrion, p, peroxisome. (b) Limiting proteins were identified by removing the thermal adjustment of associated $k_{\mathrm{cat}}$ values (cf Materials and Methods section; Dataset S7). Shown is the number and overlap in the predicted limiting proteins at two temperatures.

We then asked whether there was a common set of metabolites found as limiting across all considered temperatures. To this end, we compared the 10 most‐limiting metabolites at temperatures from 10°C to 40°C (Fig. 3a). As a result, we found that while more than half of these metabolites were limiting across the whole temperature range, some (e.g. l‐arginine, l‐glutamine, citrulline, ornithine, and citrate) were only limiting at temperatures above 35°C. The identified most‐limiting metabolites feed into the amino acid synthesis, TCA cycle, starch synthesis, and the Calvin–Benson Cycle. It has been shown that the application of individual amino acids (e.g. l‐arginine, l‐glutamine), or amino acid‐containing biostimulants, can improve heat stress resistance of plant growth (Kauffman *et al*., 2007; Matysiak *et al*., 2020; Francesca *et al*., 2022), supporting the predicted growth‐limiting metabolites. Notably, most of the identified growth‐limiting metabolites were also identified by the AraCore model with only steady‐state constraints (88%). However, the temperature‐dependent ecAraCore model yielded temperature‐specific limitations that cannot be identified using the original AraCore model.

To identify proteins that pose thermal limitations on the network, we removed the temperature adjustment from $k_{\mathrm{cat}}$ values of single proteins and quantified the resulting change in RGR compared with the fully constrained model. These simulations mimic the engineering of thermotolerant proteins whose catalytic rates are not affected by temperature changes. The analysis was performed at temperatures between 10°C and 40°C in 5°C intervals. As a result, we identified two proteins that led to a predicted increase in RGR when their temperature dependence was relieved only at 10°C (Fig. 3b; Dataset S7): These included the large RuBisCO subunit (rbcL) and Cytochrome b6‐f subunit 5 (petG), indicating that these proteins were not affected by any temperature change above 10°C. By contrast, we found 31 proteins that increased the predicted RGR only at 40°C when temperature adjustments of their properties were not imposed. Of these, nine were carbonic anhydrases (ACA, BCA), four cytosolic fructose‐bisphosphate aldolases (FBA), two chloroplastic glyceraldehyde‐3‐phosphate dehydrogenases (GAPA), as well as components of photosystem I. Further, we found RuBisCO small subunits among the limiting proteins, which have been found as differentially expressed in *A. thaliana* plants grown at 10°C and 30°C (Cavanagh *et al*., 2023), indicating their temperature‐dependent role on growth. Interestingly, we found the RuBisCO activase (RCA) to be limiting at both 10°C and 40°C. This finding is supported by previous studies, which identified a temperature optimum of RCA activity in tobacco (Crafts‐Brandner & Salvucci, 2000), spinach (Yamori *et al*., 2006), and sweet potato (Cen & Sage, 2005).

### Experimental validation of reduced growth in knockout lines at suboptimal temperature

To investigate the applicability of our model in generating sophisticated metabolic engineering solutions, we first asked whether it can accurately predict the effects of gene knockouts on temperature‐dependent growth in *A. thaliana*. To this end, we simulated knockouts of reactions by fixing their fluxes to zero, thus avoiding issues related to functionally redundant proteins (i.e. isozymes that catalyze the same reaction, Dataset S8). We predicted 37 reactions whose knockouts did not cause lethality but reduced RGR with respect to the WT at 17°C, an ecophysiologically relevant temperature for *A. thaliana* (Todesco *et al*., 2010). Importantly, none of these reactions or underlying genes could be identified by the original AraCore model that considers only steady‐state constraints. Reactions that did not show any significant reduction in RGR at any temperature up to 40°C served as negative (no effect) instances.

**Fig. 4:**
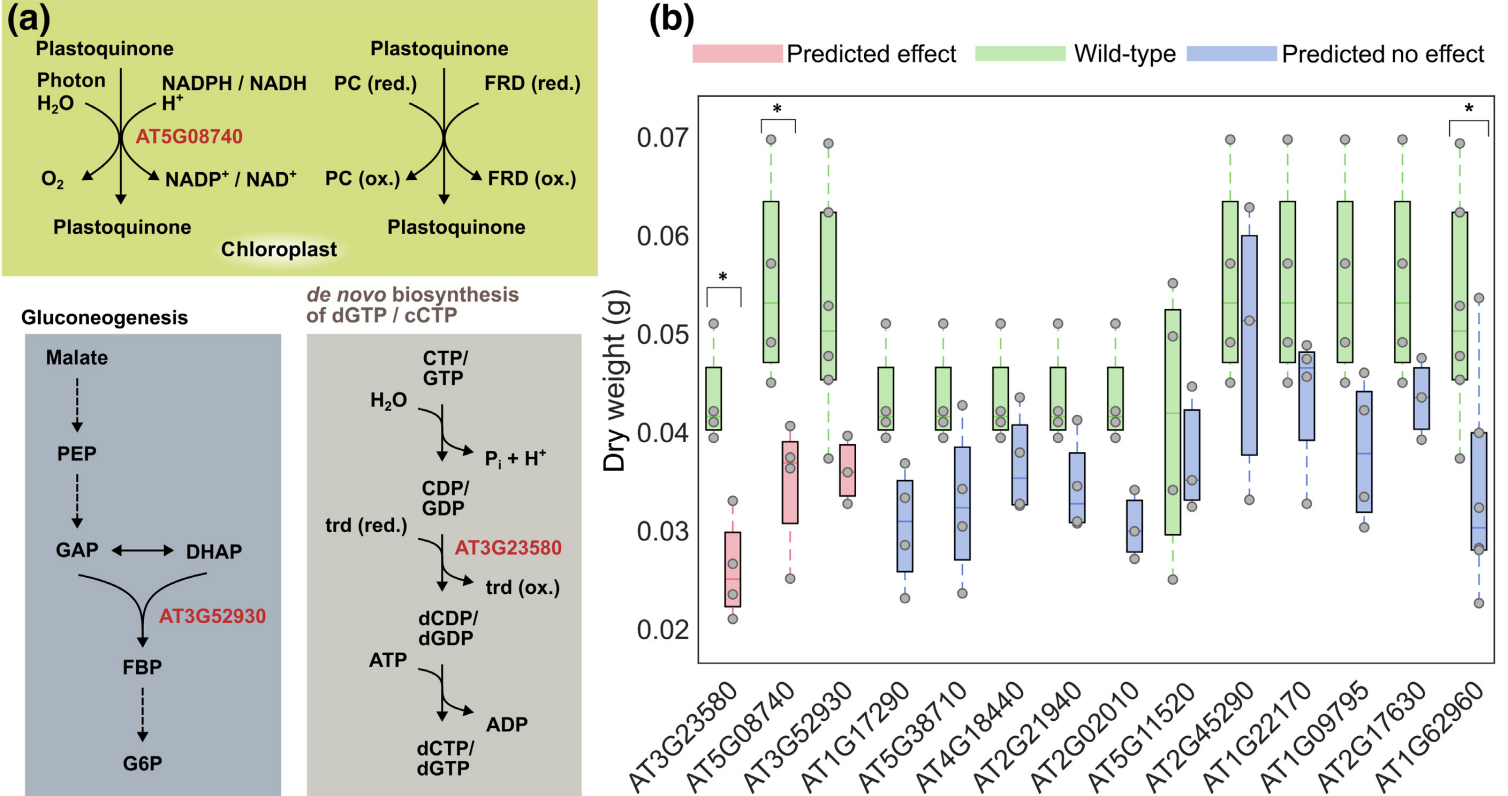
Experimental testing of predicted temperature‐dependent growth phenotypes. (a) Reactions and pathways affected by the single gene knockouts (red color), which were predicted to reduce the relative growth rate (RGR) in *Arabidopsis thaliana* Col‐0 at 17°C (cf Supporting Information Dataset S8 for all knockout predictions). (b) Dry weight of tested *A. thaliana* T‐DNA insertion lines with a predicted decrease (effect) or no change in RGR. Lines with no effect on RGR were those for which no change in RGR was predicted for temperatures up to 40°C. The plants were grown at 23°C for 10 d before transfer to 17°C for 12 d. The dry weight of each line was measured by pooling four plants grown in a single plot (cf Dataset S9). Considering that the plants were grown in four separate batches, the boxplots show the distribution of dry weights for each knockout line alongside the values of the wild‐type grown in the same batch (cf Materials and Methods section). Box plots show the interquartile range (IQR), the middle line represents the median, and vertical lines represent whiskers that either extend to 1.5‐times IQR or the minimum or maximum value, respectively. Stars over boxes indicate a significant difference to the wild‐type within the respective experimental batches as determined using a linear mixed‐effect model (lmer) and *post hoc* test (emmeans) with Benjamini–Hochberg correction of *P*‐values (Table S5). *, *P* < 0.05.

To experimentally validate our predictions, we selected *A. thaliana* T‐DNA insertion lines for three genes underlying the reactions predicted to reduce growth at 17°C and 11 genes predicted not to have any effect on growth (Fig. 4a). The selection was based on the availability of T‐DNA lines for the genes associated with the reactions in the model. All T‐DNA lines were grown at 23°C for 10 d and then shifted to 17°C for 12 d and scored for dry weight (Table S3; Dataset S9). Comparison of the measured dry weights between T‐DNA lines and the WT validated the predictions, supported by Matthews' correlation coefficient between 0.45 and 0.58, depending on the *post hoc* test used (Table S5). Of three knockout mutants with a predicted growth reduction, two were significantly different from the WT (Fig. 4b, true positives). By contrast, of 11 knockout mutants that were predicted to show no temperature‐sensitive growth phenotype, 10 showed no significant difference in the WT (true negatives). These experiments demonstrated that our model can accurately identify genes affecting growth at different temperatures and can be used to develop engineering strategies to modify growth for unseen future temperature scenarios.

## Discussion

Here, we presented the first temperature‐dependent metabolic model for *A. thaliana* – a plant for which sufficient data are available for parameterization and extensive *in silico* testing. The inclusion of all reactions in the central metabolism of *A. thaliana* allowed us to make fine‐grained predictions about the thermal metabolic flexibility of individual metabolic steps as well as of relative growth rate, as a key phenotype closely linked to metabolism. This is a significant improvement over past efforts that have considered temperature effects on lumped reactions and pathways modeling photosynthesis (Kannan *et al*., 2019; Herrmann *et al*., 2020; Inoue & Noguchi, 2021). As a result, our temperature‐dependent enzyme‐constrained model, ecAraCore, facilitated the identification of temperature‐specific growth‐limiting metabolites and proteins, thus directly pointing at cultivation management techniques (e.g. targeted supplementation of nutrients) and engineering targets.

Our predictions about relative growth rate and net CO_2_ assimilation rate were supported by the available data for the *A. thaliana* Col‐0 accession (Figs 2a,b, S12). Importantly, the effects of reaction knockouts on growth at specific suboptimal growth temperature were experimentally validated with measurements in mutant lines. These findings demonstrated that *in silico* modeling of plant metabolism that considers temperature effects on enzyme properties, protein content, and photosynthesis provides a first significant step toward closing the gap in accurate prediction of plant resilience to temperature. Notably, the model showed robust predictions of RGR and A when parameter uncertainties were considered. Among the tested parameters, the model was most sensitive to changes in the $k_{\mathrm{cat}}$ values of RuBisCO for O_2_ and CO_2_, as well as $J_{\max}$.

In this study, we focused on the production of biomass precursors as the main optimization objective; however, alternative objectives may be more or equally suitable for modeling metabolic fluxes under temperature stress. Such an objective could be identified by systematically measuring biomass compositions over a temperature range, and estimating changes in maintenance costs (Cheung *et al*., 2013). While growth is a very likely function to be optimized under optimal growth conditions in the vegetative stage, any applied stress could potentially alter the objective, diverting resources from biomass production. Nevertheless, biomass precursors still must be produced, even under stress. Moreover, ATP maintenance cost, which is included in the biomass, will also apply. Therefore, we used the biomass reaction under all tested scenarios.

While the results showed good correspondence between the predicted and measured RGR and A values at different temperatures, the predicted RGR optimum of 30.2°C was higher than expected for *A. thaliana* Col‐0. Likely, this observation results from uncertainties in the temperature models for the total protein content ($P_{\mathrm{tot}}$) or $k_{\mathrm{cat}}$ values. Considering that $P_{\mathrm{tot}}$ strongly affects the predicted RGR in enzyme‐constrained metabolic models, the temperature‐dependent model for this parameter will shape the overall response of RGR to temperature. Here, we relied on values from the literature to fit a temperature function for $P_{\mathrm{tot}}$, which was expected to result in a more generalizable model. However, the precision of the model would likely benefit from using measurements from a single experiment under controlled conditions. Another factor that introduced uncertainties concerns the models to predict optimal temperatures for unmeasured proteins. While the machine learning model achieved a good performance in predicting $T_{\mathrm{opt}}$ across the species in the Meltome Atlas (Jarząb *et al*., 2020), we observed that the predicted $T_{\mathrm{opt}}$ tended to be higher than the $T_{\mathrm{opt}}$ inferred directly from melting curves. Therefore, this shift may have caused the observed discrepancy in the RGR temperature optimum.

Nevertheless, the developed framework paves the way for modeling the effects of protein signaling cascades which link temperature sensing with metabolism (Ohama *et al*., 2017). Considering that our temperature‐dependent enzyme‐constrained model can easily be applied with approaches from the constrained‐based modeling framework, it also facilitates the engineering of more refined engineering strategies (e.g. gene overexpression and/or knockdown). The predictions of temperature‐dependent responses on growth are accompanied by predictions of corresponding enzyme abundance changes, which can be explored with dedicated quantitative proteomics studies – raising further targets for rational engineering of thermal resilience. Most importantly, coupled with quantitative metabolomics studies to characterize temperature‐dependent changes in major biomass components, our model can be directly expanded to the resource allocation level (Goelzer *et al*., 2024).

Additionally, the presented temperature‐dependent constraint sets can be transferred to crop species. To capitalize on this development, a metabolic model for the species of interest must be available, which exist for multiple agriculturally relevant crop species (Hay & Schwender, 2011; Simons *et al*., 2014; Yuan *et al*., 2016b; Chatterjee *et al*., 2017; Botero *et al*., 2018; Moreira *et al*., 2019; Gerlin *et al*., 2022). To extend the metabolic model by enzyme constraints, the required $k_{\mathrm{cat}}$ values can be obtained from public databases, using the value from the closest relative of unavailable (Domenzain *et al*., 2022). Alternatively, there exist multiple pipelines for predicting $k_{\mathrm{cat}}$ values (e.g. Kroll *et al*., 2023) or left out by scaling the upper bound of reaction fluxes (Chang *et al*., 2013). Moreover, key temperatures of protein thermostability are required to render $k_{\mathrm{cat}}$ values temperature‐dependent. These values can be either predicted using the developed machine learning model or experimentally measured following established protocols (Volkening *et al*., 2019; Jarząb *et al*., 2020; Lyu *et al*., 2023). To adapt the constraints from the FvCB model, values estimated from gas exchange measurements can be used in combination with the available temperature models. Finally, temperature‐dependent measurements of the total protein content are needed, which can easily be generated. Therefore, in future studies, our model can be applied as a template for rational engineering of crops with improved thermal resilience.

## Competing interests

None declared.

## Author contributions

PW and ZN wrote the original draft of the manuscript. ZN, PW, GMA and RAEL viewed and edited the manuscript. PW and ZN created display items. PW and ZN designed the research and developed the theoretical framework. PW wrote the computer code and conducted the simulations. RAEL and GMA designed the growth experiment with T‐DNA lines. GMA carried out the growth experiment. ZN and RAEL supervised the theoretical and experimental analyses. ZN acquired funding and administered the project.

## Disclaimer

The New Phytologist Foundation remains neutral with regard to jurisdictional claims in maps and in any institutional affiliations.

## Supporting information


**Dataset S1** Sequence‐based features for machine learning of protein thermostability optima.
**Dataset S2** Total protein content at different temperatures.
**Dataset S3** Experimental measurements of relative growth rates of *Arabidopsis thaliana* Col‐0 at different temperatures.
**Dataset S4** Experimental measurements of the net CO_2_ assimilation rate for *Arabidopsis thaliana* Col‐0 at different temperatures.
**Dataset S5** K‐medoids clustering of predicted growth responses to metabolite supplementation at different temperatures (irradiance: 150 μmol m^−2^ s^−1^).
**Dataset S6** K‐medoids clustering of predicted growth responses to metabolite supplementation at different temperatures (irradiance: 400 μmol m^−2^ s^−1^).
**Dataset S7** Responses in relative growth rate upon relief of *k*_cat_ adjustment of each protein individually at different temperatures (irradiance: 400 μmol m^−2^ s^−1^).
**Dataset S8** Predicted reduction in relative growth rate upon single gene knockouts.
**Dataset S9** Fresh and dry weights of different Arabidopsis T‐DNA lines and the Col‐0 wild‐type.

**Fig S1** Schematic representation of the fructose‐bisphosphate aldolases approach.
**Fig. S2** Schematic representation of the developed constraint‐based optimization workflow.
**Fig. S3** Function fits to leaf dry mass per area measured at different temperatures.
**Fig. S4** Key temperatures of thermostability and adjusted $k_{\mathrm{cat}}$ values.
**Fig. S5** Fitted parameters of the macromolecular rate theory function to describe the relationship between $k_{\mathrm{cat}}$ and temperature.
**Fig. S6** Function fits to the total protein content of *Arabidopsis thaliana* at different temperatures.
**Fig. S7** Influence of parameter γ, which models the O_c_ to C_c_ ratio, on the predicted relative growth rate and flux distributions.
**Fig. S8** Silhouette Index of K‐medoids clustering with different cluster numbers.
**Fig. S9** Temperature dependence of the Farquhar, von Caemmerer, and Berry model parameters.
**Fig. S10** Prediction of electron transport‐limited net CO_2_ assimilation rate ($A_{j}$) with different temperature models for $J_{\max}$.
**Fig. S11** Comparison of different temperature for stomatal conductance ($g_{s}$) in *Arabidopsis thaliana*.
**Fig. S12** Correlation of predicted and measured net CO_2_ assimilation rate (A) under different conditions.
**Fig. S13** Robustness analysis of the temperature‐dependent ecAraCore model.
**Fig. S14** Sensitivity analysis for the temperature‐dependent ecAraCore model.
**Fig. S15** Operational flux ranges at different temperatures.
**Fig. S16** Distribution of flux through selected pathways at different temperatures.
**Fig. S17** Distribution of flux through amino acid synthesis pathways at different temperatures.
**Fig. S18** Similarity between growth‐limiting metabolites found at different temperatures.
**Fig. S19** K‐medoids clustering of predicted growth responses to metabolite supplementation at $I=150\;\text{μmol}\;m^{-2}s^{-1}$.
**Fig. S20** K‐medoids clustering of predicted growth responses to metabolite supplementation $I=400\;\text{μmol}\;m^{-2}s^{-1}$.
**Methods S1** Detailed descriptions of the constraint‐based modeling workflow and derivation of temperature‐dependent constraints.
**Table S1** Experimental data and temperature dependences used to parameterize the Farquhar, von Caemmerer, and Berry model.
**Table S2** Experimental data on the leaf mass per area at different temperatures, which were used to describe the temperature dependence of LMA.
**Table S3** *Arabidopsis thaliana* T‐DNA insertion mutant lines assessed for their leaf development at 17°C.
**Table S4** Performance of different regression models with default parameters that were trained using the reduced feature set after feature selection.
**Table S5** Statistical comparison of measured plant dry weights of *Arabidopsis thaliana* T‐DNA insertion lines to the Col‐0 wild‐type using a linear mixed‐effect model.
Please note: Wiley is not responsible for the content or functionality of any Supporting Information supplied by the authors. Any queries (other than missing material) should be directed to the *New Phytologist* Central Office.

## Data Availability

The custom computer code that was developed for simulations and statistical analyses in this study is publicly available at https://github.com/pwendering/AraTModel. The code developed for machine learning of protein thermostability optima was deposited in a separate repository, which is publicly available at https://github.com/pwendering/topt‐predict. The refined AraCore model can be retrieved from https://github.com/pwendering/ArabidopsisCoreModel. All remaining data that can be used to replicate the study are provided with this manuscript and Supporting Information (Tables S1–S3; Datasets S1–S4, S9).
