# Supplementary material for: Metabolic modeling identifies determinants of thermal growth responses in Arabidopsis thaliana
Source: New Phytol. 2025 Jan 24;247(1):178–90. doi: 10.1111/nph.20420 (PMC12138187; doi:10.1111/nph.20420)
Supplement: Supplementary file 2 — Fig S1 Schematic representation of the fructose‐bisphosphate aldolases approach. Fig. S2 Schematic representation of the developed constraint‐based optimization workflow. Fig. S3 Function fits to leaf dry mass per area measured at different temperatures. Fig. S4 Key temperatures of thermostability and adjusted kcat values. Fig. S5 Fitted parameters of the macromolecular rate theory function to describe the relationship between kcat and temperature. Fig. S6 Function fits to the total protein content of Arabidopsis thaliana at different temperatures. Fig. S7 Influence of parameter γ, which models the Oc to Cc ratio, on the predicted relative growth rate and flux distributions. Fig. S8 Silhouette Index of K‐medoids clustering with different cluster numbers. Fig. S9 Temperature dependence of the Farquhar, von Caemmerer, and Berry model parameters. Fig. S10 Prediction of electron transport‐limited net CO2 assimilation rate (Aj) with different temperature models for Jmax. Fig. S11 Comparison of different temperature for stomatal conductance (gs) in Arabidopsis thaliana. Fig. S12 Correlation of predicted and measured net CO2 assimilation rate (A) under different conditions. Fig. S13 Robustness analysis of the temperature‐dependent ecAraCore model. Fig. S14 Sensitivity analysis for the temperature‐dependent ecAraCore model. Fig. S15 Operational flux ranges at different temperatures. Fig. S16 Distribution of flux through selected pathways at different temperatures. Fig. S17 Distribution of flux through amino acid synthesis pathways at different temperatures. Fig. S18 Similarity between growth‐limiting metabolites found at different temperatures. Fig. S19 K‐medoids clustering of predicted growth responses to metabolite supplementation at I=150μmolm−2s−1. Fig. S20 K‐medoids clustering of predicted growth responses to metabolite supplementation I=400μmolm−2s−1. Methods S1 Detailed descriptions of the constraint‐based modeling workflow and derivation of temperature‐dependent con [file NPH-247-178-s002.docx]

***New Phytologist* Supporting Information**

Article title: Metabolic modeling identifies determinants of thermal growth responses in *Arabidopsis thaliana*

Authors: Philipp Wendering, Gregory M. Andreou, Roosa A. E. Laitinen, Zoran Nikoloski

Article acceptance date: 9 January 2025

# Methods S1. Detailed descriptions of the constraint-based modeling workflow and derivation of temperature-dependent constraints. Here, the constraint-based modeling workflow is described in greater detail, proving background on basic constraint-based modeling approaches. Further, the derivation of temperature-dependent constraints is provided, including additional details on the prediction of missing $\boldsymbol{T}_{\boldsymbol{opt}}$ values. Finally, the resulting optimization problem as well as formulations for flux variability analysis and sampling are given.

## Constraint-based modeling workflow

### Metabolic model

All (temperature-dependent) flux simulations described in this study were performed using a refined version of the Arabidopsis core (AraCore) model (Arnold & Nikoloski, 2014). The model captures the reaction stoichiometries and gene associations of *Arabidopsis thaliana*’s primary metabolism. For the use in this study, the model was refined (c.f. “Refinement and extensions of the Arabidopsis core model”) and now comprises 415 metabolites, 585 reactions, and 706 genes. The AraCore model is a stoichiometric model, designed for simulation of cellular metabolism using constraint-based optimization. To enable efficient mathematical modeling, the individual reactions are represented as columns in a stoichiometric matrix, $\mathbf{S}$, where the row coefficients represent the stoichiometric coefficients of the metabolites that take part in the reactions. Besides enzymatic reactions, the model contains exchange (input or output) reactions for essential nutrients (e.g., H_2_O, NO_3_^-^, CO_2_, or O_2_). Moreover, it contains a pseudo-reaction, $v_{bio}$, which models the generation of biomass from its generated precursors using their respective molar fractions.

### Flux Balance Analysis

The fluxes, $\mathbf{v}$, through the reactions at metabolic steady state (i.e., metabolite concentrations do not change over time), can be simulated using flux balance analysis (FBA). FBA is a constraint-based optimization framework, where the flux through the biomass reaction is maximized (i.e., growth rate, referred to as RGR) while the metabolic system is at steady state. This is encoded in the following set of equations:

|  | $\max v_{bio}$ | (S1) |
| --- | --- | --- |

subject to

|  | $\mathbf{S}\cdot\mathbf{v}=\mathbf{0}$ | (S2) |
| --- | --- | --- |
|  | $\mathbf{v}^{\mathbf{min}}\leq\mathbf{v}\leq\mathbf{v}^{\mathbf{max}}$ | (S3) |

The equation (S3) denotes the lower and upper bounds imposed on each of the fluxes. Here, we used a value of $1000 mmol gDW^{-1}h^{-1}$ ($gDW$: gram dry weight). Notably, FBA predicts a single steady state flux distribution ($\mathbf{v}$) that results in the maximum RGR. However, since there are fewer metabolites and resulting mass-balance equation than reactions in the model, the system is underdetermined. This means that multiple solutions to this problem are possible. Therefore, experimental data can be integrated as additional constraints to narrow down the space of possible solutions to the ones that are biologically relevant (Fig. S1).


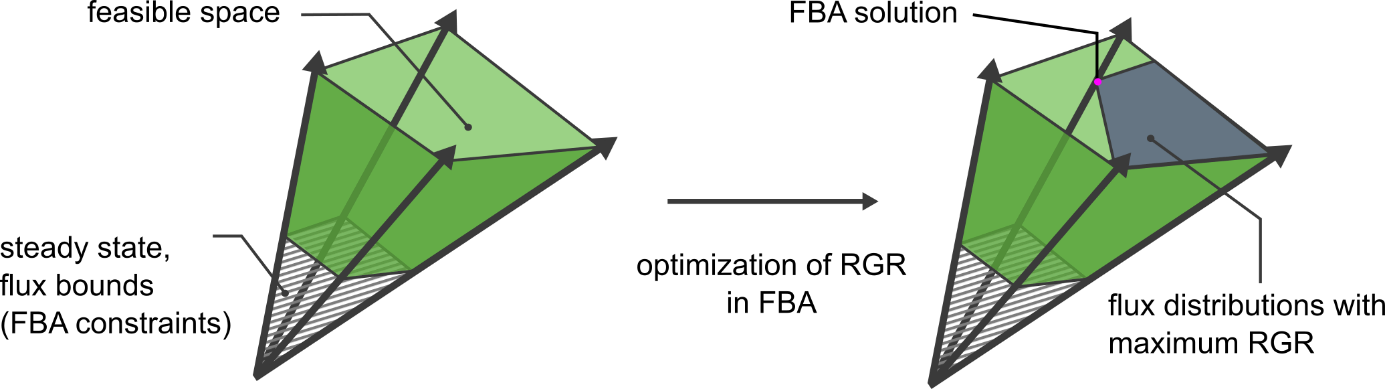


**Fig S1. Schematic representation of the FBA approach.** By applying constraints on the metabolic steady state and bounds on reaction fluxes, the feasible space becomes smaller (left). The optimization of the biomass objective yields a single flux distribution corresponding to the maximum growth rate (RGR) (right, magenta). However, there may be multiple alternative flux distributions that also allow for the same maximum RGR value (blue). FBA: flux balance analysis

### Enzyme constraints

To enable the investigation of temperature effects on enzyme kinetics and protein stability, the AraCore model was extended to an enzyme-constraint model, termed ecAraCore, using the GECKO toolbox v2.0.2 (Sánchez *et al.*, 2017). Here, the flux of a reaction is limited by the product of enzyme abundance $\left[ E \right]$ and turnover number ($k_{cat}$):

|  | $v\leq\left[ E \right]\cdot k_{cat} .$ | (S4) |
| --- | --- | --- |

Further, the sum of enzyme abundances is limited by a fraction of the total protein content, $P_{tot}$:

|  | $\sum_{i} \left[ E_{i} \right]\cdot\mathrm{MW}_{i}\leq P_{tot}\cdot f\cdot\sigma.$ | (S5) |
| --- | --- | --- |

The fraction of the total protein content is determined by the factors $f$ and $\sigma$, representing the coverage of all proteins of the organism by the model and the average enzyme saturation, respectively; $\mathrm{MW}$ denotes the molecular weight of the respective enzyme. Since the integration of $f$ and $\sigma$ led to unrealistically small predictions of relative growth rate, both were set to one.

### Photosynthesis and gas exchange constraints

Based on the C_3_ photosynthesis model by Farquhar et al. (Farquhar *et al.*, 1980) (FvCB model), as well as mesophyll and stomatal conductance, additional constraints were derived to model (1) the ratio between the RuBisCO oxygenation and carboxylation reactions, (2) the net CO_2_ assimilation rate ($A$), (3) the uptake of CO_2_ from the air, and (4) the impact on light and CO_2_ on $A$. The derivation of the constraints is explained in more detail in the section “Derivation of constraints based on the FvCB model”.

### Introduction of temperature dependences

The effects on temperature on metabolic fluxes were considered its impacts on (1) enzyme kinetics, (2) the total protein content, and (3) photosynthesis. Briefly, the effects of temperature on enzyme kinetics ($k_{cat}$ values) were incorporated into the by assuming that the catalytic optimum of the enzyme is equal to its stability optimum and that its catalytic rate is limited by the availability of native enzyme at high temperatures. Following these assumptions, a temperature model for enzyme kinetic rates (Hobbs *et al.*, 2013) was fitted for each $k_{cat}$ value in the model (see section “Temperature adjustment of turnover numbers” for more details). Further, experimental data on the total protein content ($P_{tot}$) for *A. thaliana* Col-0 at different temperatures were obtained from the literature to derive an empirical model (see section “Temperature dependence of total protein content” for more details). The equations (S4) and (S5) change accordingly:

|  | $v\leq\left[ E \right]\cdot k_{cat}\left( T \right)$ | (S6) |
| --- | --- | --- |
|  | $\sum_{i} \left[ E_{i} \right]\cdot\mathrm{MW}_{i}\leq P_{tot}\left( T \right)\cdot f\cdot\sigma.$ | (S7) |

The temperature dependences on photosynthesis and CO_2_ uptake were introduced by considering the temperature dependences of the individual parameters as listed in Table S1.

Together, these additional constraints and their temperature dependences further narrow down the solution space of the constraint-based problem (Fig. S2a).


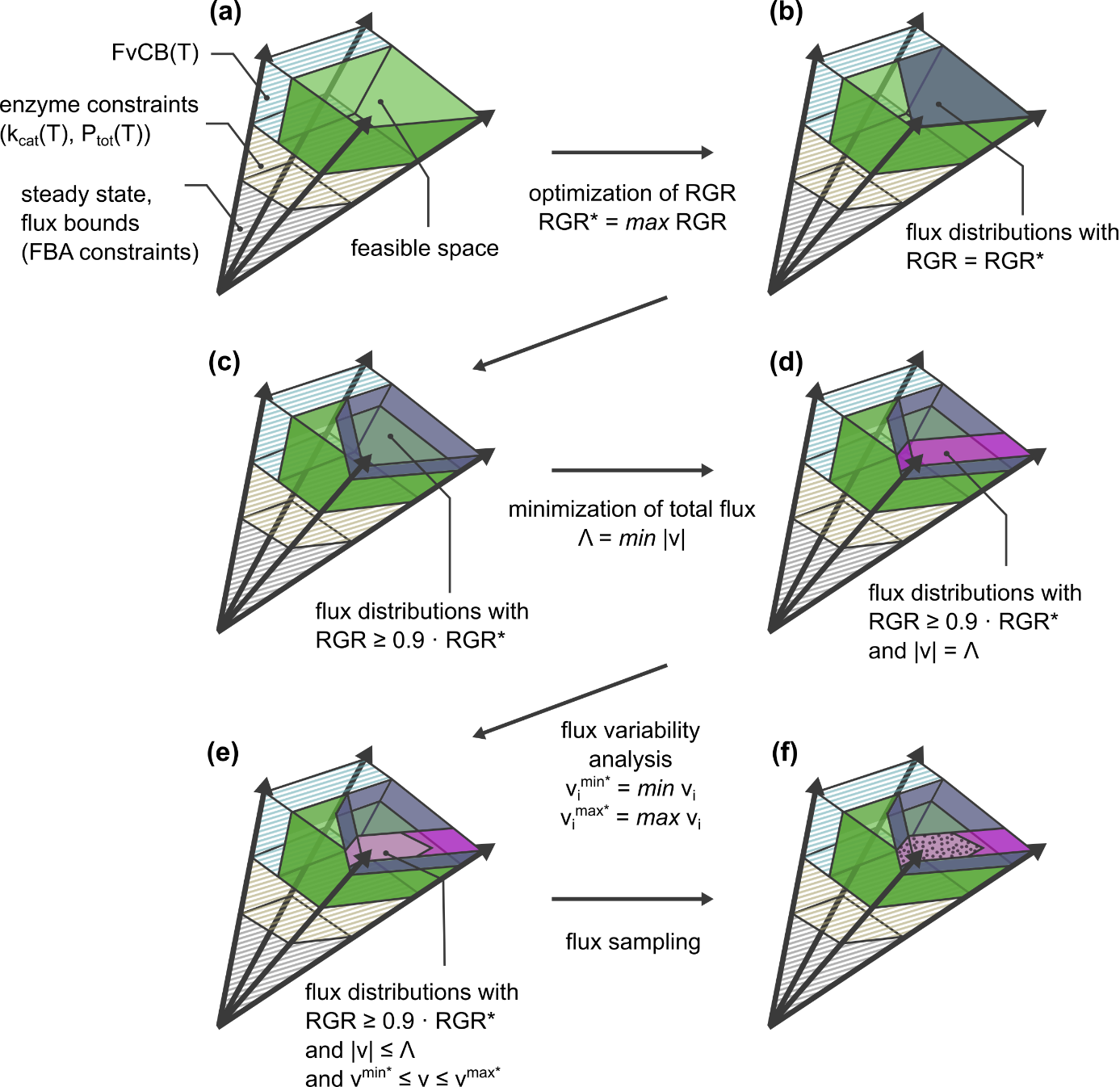


**Fig S2. Schematic representation of the developed constraint-based optimization workflow. (A)** The solution space of possible flux distributions is narrowed by applying constraints from flux balance analysis (FBA), (temperature-dependent) enzyme kinetics, and (temperature-dependent) photosynthesis and CO_2_ uptake. **(B)** Optimization of the relative growth rate (RGR) yields the maximum RGR. The space of solutions yielding the maximum RGR value is indicated in blue. **(C)** For further analysis, the solution space is constrained by a lower limit of 90% of the maximum RGR value for the exploration of the solution space. For the two-step procedure to predict the net assimilation rate, a lower limit of 99% of the maximum RGR values was applied. **(D)** The sum of fluxes was minimized and then fixed to the minimum value. This additional constraint narrowed down the solution space (magenta). **(E)** Subsequently, each individual flux was minimized and maximized subject to all previous constraints to narrow down the space for flux sampling (light magenta). **(F)** Finally, flux distributions were sampled within the constrained solution space. T: temperature, FvCB: Farquar-von Caemmerer-Berry model, P_tot_ total protein content, v: vector of reaction fluxes, Λ: minimum sum of fluxes, v^min^ and v^max^: vectors of minimum and maximum fluxes for each reaction

### Prediction of RGR and A

The predictions of RGR and $A$ were carried out in two steps: (1) optimization of RGR and (2) minimization of total flux. The first step extends the FBA approach by applying additional constraints that shape the solution space (Fig. S2a). As depicted in Fig. S2b, multiple flux distributions will result in the same RGR optimum. However, we found that the values for $A$ in the distributions obtained in the first step are not realistic, mainly because the CO_2_ export from the mitochondrion to the cytosolic compartment is overestimated. Therefore, we applied the principle of parsimonious enzyme use (i.e., pFBA (Lewis *et al.*, 2010)), which is commonly used in constraint-based metabolic modeling of plant metabolism (e.g., Lewis *et al.*, 2010; Shaw & Cheung, 2018, 2021; Gerlin *et al.*, 2022; Simas Coutinho Barbosa *et al.*, 2024). To this end, the sum of absolute flux values was minimized at 99% of the optimum RGR value to avoid numerical instabilities (Fig. S2c,d, here $0.99\cdot RGR^{*}$ instead of $0.9\cdot RGR^{*}$). The reported values for $A$ originate from the flux distribution obtained in this second step. The full optimization problem is shown in section “Optimization problem for prediction of temperature-dependent relative growth rate”.

### Exploration of the solution space

To further investigate the space of alternative solutions, we conducted flux sampling at 90% of the optimal RGR and while keeping the sum of fluxes to the minimum value (Fig. S2e,f). The applied sampling approach relied on generating random flux vectors and finding the closest feasible points within the constrained optimal space (Fig. S2e). To facilitate this projection, the minimum and maximum flux values under these constraints were obtained flux variability analysis and subsequently used as limits for the respective random flux values. The full optimization problems for flux variability analysis and sampling are shown in section “Flux variability analysis and flux sampling”.

## Prediction of missing $\boldsymbol{T}_{\boldsymbol{opt}}$ values (continued)

For the 47% of proteins in the ecAraCore model where $T_{opt}$ could not be inferred from TPP data, they were predicted using the Random Forst regression model. The model was trained to predict $T_{opt}$ based on amino acid features of the protein sequences. To this end, $T_{opt}$ for all proteins in the Meltome Atlas were obtained using the *beta growth function* and amino acid sequences were downloaded from UniProt (Bateman *et al.*, 2021). All *Homo sapiens* data sets were excluded as it was not possible to decide which proteins to include from Jurkat or K562 cells. Further, all “cells” data sets were not considered, ensuring usage of data sets that originate only from lysed cells. The *Mus musculus* BMDC lysate was also not included due to duplicated protein entries, which did not allow for an unambiguous mapping between proteins and fractions of native protein. To guarantee sufficient quality of the training data, only fits with $R_{adj}^{2}\geq0.6$ were used for machine learning of $T_{opt}$.

Amino acid sequence features were extracted using iFeatureOmega (Chen *et al.*, 2022), the ProtParam module of Biopython (Cock *et al.*, 2009), and the protlearn module. Further, five amino acid groups were defined as for Protstab2 (Yang *et al.*, 2022). In total, 2839 features were extracted for 15840 protein sequences. The features "SEQL" (sequence length), "MW" (molecular weight), "MEXTC_1” (molar extinction coefficient) were logarithmized before further processing.

To determine the smallest feature set with acceptable performance, a recursive feature elimination with 5-fold cross-validation was performed using a Random Forest regression model with default settings and a step size of 10. As a result, 69 features were selected (Dataset S1). The data set with reduced features was then divided into a training (60%) and a validation set (40%) and the predictive performance of nine other regression models was assessed with default parameters (Table S4). Prior to the regression, the training and test set were standardized based on the distribution of the training set. Since the Random Forest regression model showed the best performance of all approaches (before and after feature selection), it was selected for the prediction of $T_{opt}$.

Next, a grid search was conducted to find optimal parameters for the Random Forest regression model. The choice of optimal parameters, after hyperparameter tuning, resulted in a slightly increased performance compared to the default settings (Table S4). The importance of features of the trained model is shown in Dataset S1. The Random Forest regression model is available via a command line interface tool (<https://github.com/pwendering/topt-predict>). In its current state, the tool further allows for automated extraction of amino acid features for given amino acid sequences, which are then standardized using the distribution properties of the training set that was used to train the Random Forest regressor.

## Derivation of constraints based on the FvCB model

### Net CO_2_ assimilation rate

The relationship between the net CO_2_ assimilation rate, carboxylation rate, oxygenation rate, and the mitochondrial respiration is fundamental to the C_3_ photosynthesis model described by Farquhar et al. (Farquhar *et al.*, 1980). Using the corresponding reaction fluxes in the ecAraCore model ($v_{c}$: carboxylation reaction, $v_{o}$: oxygenation reaction, $v_{{exCO}_{2}}^{mito}$: export of CO_2_ from the mitochondrial compartment), we arrive at the following constraint:

|  | $A=v_{c}-\alpha v_{o}-v_{{exCO}_{2}}^{mito}.$ | (S8) |
| --- | --- | --- |

The variable $\alpha$ denotes the number of molecules CO_2_ released during photorespiration of one molecule O_2_. For the simulations, α was set to 0.5 as commonly done, but we note that its value may be dependent on temperature and has been shown to vary in photorespiratory mutants (Cousins *et al.*, 2008, 2011; Walker & Cousins, 2013). Further, the ratio between fluxes of the oxygenation and carboxylation reaction of RuBisCO is described by $\phi$, which can be calculated using the relative specificity of RuBisCO for CO_2_ over O_2_ ($S_{c/o}$) (von Caemmerer *et al.*, 2009):

|  | $\phi=\frac{1}{S_{c/o}}\cdot\frac{O_{c}}{C_{c}}=\frac{K_{c}\cdot k_{o}}{k_{c}\cdot K_{o}}\cdot\frac{O_{c}}{C_{C}}.$ | (S9) |
| --- | --- | --- |

As shown in Eq. (S9), $S_{c/o}$ can be expressed in terms of kinetic constants of RuBisCO ($K_{c}$: Michaelis-Menten constant ($K_{M}$) for the carboxylation reaction, $K_{o}$: $K_{M}$ value of the oxygenation reaction, $k_{c}$: $k_{cat}$ value of the carboxylation reaction, $k_{o}$: $k_{cat}$ value of the oxygenation reaction) and the partial pressures of O_2_ and CO_2_ at the carboxylation site ($O_{c}$ and $C_{c}$). The dependence of $\phi$ on temperature is introduced through the temperature dependences of these four kinetic constants (cf. Table S1).

With this information available, the ratio between $v_{o}$ and $v_{c}$ can be fixed by $\phi\left( T \right)$, allowing a deviation of $\tau=0.001$:

|  | $\left( 1-\tau\right)\phi v_{c}\leq v_{o}\leq\left( 1+\tau\right)\phi v_{c}.$ | (S10) |
| --- | --- | --- |

Using the two constraints from Eqs. (S8) and (S10), we can determine the value of $A$, and the ratio, $\phi$, of the oxygenation and carboxylation rate, can be set according to a given temperature. If $\phi$ was not set as described, flux through RuBisCO would only be directed through the carboxylation reaction when growth is optimized. Hence, no flux would go through the oxygenation reaction, which is not physiologically meaningful.

### Temperature-dependent uptake of CO_2_

Since CO_2_ influx directly impacts the growth rate under non-saturating conditions, its uptake must be limited appropriately to obtain realistic growth rates. Hence, the flux must be constrained, but the ambient $p(CO_{2})$ cannot be used directly to limit the import reaction in the metabolic model. To overcome this problem, we made use of the relationships between $A$, $C_{a}$, $C_{i}$, $C_{c}$, stomatal conductance, $g_{s}$, and mesophyll conductance, $g_{m}$, known from gas exchange experiments.

Niinemets and coworkers described the relationship between $A$ and the mesophyll conductance using the difference between intercellular and chloroplast partial pressures of CO_2_ ($C_{i},C_{c}$) (Niinemets *et al.*, 2009):

|  | $A=\left( C_{i}-C_{c} \right)g_{m}.$ | (S11) |
| --- | --- | --- |

As the ambient CO_2_ partial pressure $C_{a}$ should be used as an input to the constraint-based analysis, $C_{a}$ must be linked to $C_{i}$. Farquhar and Wong described the relationship between ambient and intercellular $p(CO_{2})$ in their empirical model as follows (Farquhar & Wong, 1984):

|  | $A=\frac{C_{a}-C_{i}}{\rho r}-E\frac{C_{a}+C_{i}}{2\rho}.$ | (S12) |
| --- | --- | --- |

The equation above describes a trade-off between the acquisition of CO_2_ at the cost of transpiration of water via stomata. The variable $\rho$ represents atmospheric pressure and $r$ is the total resistance to CO_2_ diffusion from ambient air to intercellular space. The total resistance $r$ is given by a weighted sum of stomatal resistance $r_{s}=1/g_{s}$ and boundary layer resistance $r_{b}$:

|  | $r=1.37r_{b}+1.6r_{s}.$ | (S13) |
| --- | --- | --- |

As in the original model by Farquhar and Wong (1984), $r_{b}$ was set to $1 m^{2} s mol^{-1}.$Given these values, the transpiration rate $E$ can be calculated as

|  | $E=\frac{e_{i}-e_{a}}{\left( r_{s}+r_{b} \right)\left( \rho-\left( e_{i}+e_{a} \right)/2 \right)}.$ | (S14) |
| --- | --- | --- |

The saturation vapor pressure was used as a proxy for the ambient water vapor pressure $e_{a}$ and the intercellular vapor pressure was approximated by $e_{i}=e_{a}-10 mbar$. Further, the value from $e_{a}$ can be calculated dependent on ambient temperature as (Murray, 1967):

|  | $e_{a}=6106.6\cdot{10}^{\frac{7.5\left( T-273.15 \right)}{T-35.85}} .$ | (S15) |
| --- | --- | --- |

To obtain an expression for $C_{i}$, Eq. (S12) can be re-arranged as follows:

|  | $A=C_{a}\left( c_{1}-c_{2} \right)-C_{i}\left( c_{1}+c_{2} \right),$ | (S16) |
| --- | --- | --- |
|  | $c_{1}=\frac{1}{\rho\cdot r};c_{2}=\frac{E}{2\rho}.$ | (S17) |

By introducing two additional constants, $c_{3}=c_{1}-c_{2}$ and $c_{4}=c_{1}+c_{2}$, and solving for $C_{i}$, we get

|  | $C_{i}=\frac{c_{3}C_{a}-A}{c_{4}}.$ | (S18) |
| --- | --- | --- |

Equivalently, Eq. (S11) was solved for $C_{i}$:

|  | $C_{i}=\frac{A}{g_{m}}+C_{c}.$ | (S19) |
| --- | --- | --- |

By equating Eqs. (S18) and (S19), we obtain the following expression for $C_{c}$:

|  | $C_{c}=\frac{{c_{3}C}_{a}-A}{c_{4}}-\frac{A}{g_{m}}.$ | (S20) |
| --- | --- | --- |

To link CO_2_ intake to the carboxylation reaction $v_{c}$ we use the definition described in the FvCB model (Farquhar *et al.*, 1980), assuming ribulose-1,5 bisphosphate saturation, and as a result constrained $v_{c}$ by

|  | $v_{c}\leq V_{cmax}\cdot\frac{C_{c}}{C_{c}+K_{c}\left( 1+\frac{O_{c}}{K_{o}} \right)},$ | (S21) |
| --- | --- | --- |

where $V_{cmax}$ denotes the maximum rate of the RuBisCO carboxylation reaction. Now, $C_{c}$ can be eliminated by Eq. (S21):

|  | $v_{c}\left( \frac{{c_{3}C}_{a}-A}{c_{4}}-\frac{A}{g_{m}}+K_{c}\left( 1+\frac{O_{c}}{K_{o}} \right) \right)\leq V_{cmax}\left( \frac{{c_{3}C}_{a}-A}{c_{4}}-\frac{A}{g_{m}} \right).$ | (S22) |
| --- | --- | --- |

As the oxygen partial pressure in the chloroplast ($O_{c}$) is unknown, we added a parameter $\gamma$, which represents the ratio between $O_{c}$ and $C_{c}$:

|  | $\gamma=\frac{O_{c}}{C_{c}}.$ | (S23) |
| --- | --- | --- |

In this study, we set $\gamma$ to the ratio between $O_{a}$ and $C_{a}$, due to the absence of data or models to describe this ratio. However, we note that $\gamma$ can be easily adjusted. To investigate the effect of the choice for $\gamma$, we tested four values of $\gamma:\left[ \frac{O_{a}}{C_{a}},\frac{O_{a}}{C_{i}}, \frac{O_{a}}{C_{c}}, \frac{O_{a}}{0.75C_{c}} \right]$, assuming that $C_{i}\approx0.75C_{a}$ and $C_{c}\approx0.75C_{i}$. As a result, we observed that different values of $\gamma$ scale the solution by a factor and lead to highly correlated flux distributions (Fig. S7). We note that $\gamma$ also affects the calculation of $\phi$ in the constraints.

By replacing $O_{c}$ in Eq. (S20) using Eq. (S23), we obtain

|  | $v_{c}\left( \frac{c_{3}C_{a}-A}{c_{4}}-\frac{A}{g_{m}}+K_{c}\left( 1+\frac{\gamma C_{c}}{K_{o}} \right) \right)\leq V_{cmax}\left( \frac{c_{3}C_{a}-A}{c_{4}}-\frac{A}{g_{m}} \right).$ | (S24) |
| --- | --- | --- |

Eq. (S24) correspond to the following equation when the parentheses are resolved:

|  | $v_{c}\frac{c_{3}C_{a}-A}{c_{4}}-v_{c}\frac{A}{g_{m}}+v_{c}K_{c}+v_{c}\frac{\gamma C_{c}K_{c}}{K_{o}}\leq V_{cmax}\left( \frac{c_{3}C_{a}-A}{c_{4}}-\frac{A}{g_{m}} \right).$ | (S25) |
| --- | --- | --- |

Next, $C_{c}$ is replaced using Eq. (S20):

|  | $v_{c}\frac{c_{3}C_{a}-A}{c_{4}}-v_{c}\frac{A}{g_{m}}+v_{c}K_{c}+\frac{v_{c}\gamma K_{c}}{K_{o}}\left( \frac{c_{3}C_{a}-A}{c_{4}}-\frac{A}{g_{m}} \right)\leq V_{cmax}\left( \frac{c_{3}C_{a}-A}{c_{4}}-\frac{A}{g_{m}} \right).$ | (S26) |
| --- | --- | --- |

To simplify this expression, we introduce an additional variable

|  | $Z=\frac{{c_{3}C}_{a}-A}{c_{4}}$ | (S27) |
| --- | --- | --- |

and an additional constant

|  | $c_{5}=\frac{\gamma K_{c}}{K_{o}}.$ | (S28) |
| --- | --- | --- |

The resulting Eq. (S29), below, was used as the third constraint to limit CO_2_ uptake by photosynthesis parameters:

|  | $v_{c}K_{c}+v_{c}Z\left( 1+c_{5} \right)-v_{c}A\left( \frac{1}{g_{m}}+\frac{c_{5}}{g_{m}} \right)\leq V_{cmax}\left( Z-\frac{A}{g_{m}} \right)$  $\Leftrightarrow v_{c}K_{c}+v_{c}Z\left( 1+c_{5} \right)-v_{c}A\left( \frac{1}{g_{m}}+\frac{c_{5}}{g_{m}} \right)-V_{cmax}Z+A\frac{V_{cmax}}{g_{m}}\leq0$ | (S29) |
| --- | --- | --- |

An overview of the parametrization and associated temperature-dependent adjustment function can be found in Table S1 and Fig. S9.

### Limitation of the photon import reaction by light-limited net CO_2_ assimilation rate

The electron-transport-limited net CO_2_ assimilation rate ($A_{j}$) according to the FvCB model (Farquhar *et al.*, 1980) is given by

|  | $A_{j}=\frac{J\left( C-\Gamma^{*} \right)}{4C-\Gamma^{*}}-R_{d},$ | (S30) |
| --- | --- | --- |

where $J$ denotes the potential rate of electron transport, $C$ denotes the CO_2_ partial pressure, $\Gamma^{*}$ is the CO_2_ compensation point without day respiration (i.e., $C$, where net assimilation of CO_2_ is equal to zero), and $R_{d}$ denotes day respiration. To integrate this constraint on $A$, $C$ must denote the CO_2_ partial pressure at the carboxylation site ($C_{c}$). From the assumption that net CO_2_ fixation is zero at the CO_2_ compensation point

|  | $\alpha\cdot\frac{v_{o}\left( \Gamma^{*} \right)}{v_{c}\left( \Gamma^{*} \right)}=\alpha\cdot\frac{O_{c}}{\Gamma^{*}}\cdot\frac{K_{c}k_{o}}{K_{o}k_{c}}=1,$ | (S31) |
| --- | --- | --- |

it follows that

|  | $\Gamma^{*}=\alpha\cdot O_{c}\cdot\frac{K_{c}k_{o}}{K_{o}k_{c}}=\frac{\alpha O_{c}}{S_{c/o}}.$ | (S32) |
| --- | --- | --- |

The parameter $\Gamma^{*}$ is thus dependent on the O_2_ partial pressure at the carboxylation site ($O_{c}$), the specificity of RuBisCO for CO_2_ over O_2_, and the number of CO_2_ molecules released during photorespiration of one molecule O_2_ ($\alpha=0.5$) (Farquhar *et al.*, 1980; von Caemmerer, 2000; Walker & Cousins, 2013). In Eq. (S20), we established a relationship between the ambient CO_2_ partial pressure ($C_{a}$) and $C_{c}$. Moreover, in Eq. (S21), the ratio between $O_{c}$ and $C_{c}$ is modeled by the tunable parameter $\gamma$, yielding an expression to describe $O_{c}$. By replacing $\Gamma^{*}$ using Eq. (S32), Eq. (S30) can be reformulated as follows:

|  | $A_{j}=\frac{J\left( C-\frac{\alpha O_{c}}{S_{c/o}} \right)}{4C-8\frac{\alpha O_{c}}{S_{c/o}}}-R_{d}.$ | (S33) |
| --- | --- | --- |

As a next step, $O_{c}$ is substituted using Eq. (S23):

|  | $A_{j}=\frac{J\left( C-\frac{\alpha\gamma C_{c}}{S_{c/o}} \right)}{4C-8\frac{\alpha\gamma C_{c}}{S_{c/o}}}-R_{d}$ | (S34) |
| --- | --- | --- |

Further, $C_{c}$ can be replaced by Eq. (S20):

|  | $A_{j}=\frac{J\left( \frac{c_{3}C_{a}-A}{c_{4}}-\frac{A}{g_{m}}-\frac{\alpha\gamma\left( \frac{c_{3}C_{a}-A}{c_{4}}-\frac{A}{g_{m}} \right)}{S_{c/o}} \right)}{4\left( \frac{c_{3}C_{a}-A}{c_{4}}-\frac{A}{g_{m}} \right)-8\frac{\alpha\gamma\left( \frac{c_{3}C_{a}-A}{c_{4}}-\frac{A}{g_{m}} \right)}{S_{c/o}}}-R_{d}.$ | (S35) |
| --- | --- | --- |

To simplify the expression, the previously introduced variable $Z$ (Eq. (S27)) is used to replace variables in Eq. (S35):

|  | $A_{j}=\frac{J\left( Z-\frac{A}{g_{m}}-\frac{\alpha\gamma\left( Z-\frac{A}{g_{m}} \right)}{S_{c/o}} \right)}{4\left( Z-\frac{A}{g_{m}} \right)-8\frac{\alpha\gamma\left( Z-\frac{A}{g_{m}} \right)}{S_{c/o}}}-R_{d}.$ | (S36) |
| --- | --- | --- |

Now, the term $Z-\frac{A}{g_{m}}$ can be placed outside the parentheses and later removed from the fraction:

|  | $A_{j}=\frac{\left( Z-\frac{A}{g_{m}} \right)J\left( 1-\frac{\alpha\gamma}{S_{c/o}} \right)}{\left( Z-\frac{A}{g_{m}} \right)\left( 4-8\frac{\alpha\gamma}{S_{c/o}} \right)}-R_{d}.$ | (S37) |
| --- | --- | --- |

By reducing the terms and introducing a variable $Y=\frac{\alpha\gamma}{S_{c/o}}$, we arrive the the following expression for $A_{j}$:

|  | $A_{j}=\frac{J\left( 1-Y \right)}{4+8Y}-R_{d}.$ | (S38) |
| --- | --- | --- |

Eq. (S38) can then be integrated into the constraint-based modeling problem as the following constraint, approximating $R_{d}$ with $v_{exCO_{2}}^{mito}$ as in Eq. (S8):

|  | $A\leq A_{j}\Longleftrightarrow v_{c}-\alpha v_{o}-v_{exCO_{2}}^{mito}\leq\frac{J\left( 1-Y \right)}{4+8Y}-v_{exCO_{2}}^{mito}$ |  |
| --- | --- | --- |
|  | $\Longleftrightarrow v_{c}-\alpha v_{o}\leq\frac{J\left( 1-Y \right)}{4+8Y}.$ | (S39) |

The parameter $J$ itself depends both on the light and temperature condition (von Caemmerer, 2000):

|  | $J=\frac{I_{2}+J_{max}-\sqrt{\left( I_{2}+J_{max} \right)^{2}}-4\theta I_{2}J_{max}}{2\theta},$ | (S40) |
| --- | --- | --- |

where $J_{max}$ denotes the light saturated electron transport rate, $\theta=0.7$ is an empirical curvature parameter (Evans, 1989) that describes that relationship of $J$ with the fraction of irradiance that can be used by the plant ($I_{2}$) (von Caemmerer, 2000). The value for $I_{2}$ is in turn approximated by

|  | $I_{2}=I\cdot q\cdot0.5\left( 1-f \right).$ | (S41) |
| --- | --- | --- |

The variable $q=0.85$ denotes the absorptance of the leaf (Evans, 1987; Evans & Terashima, 1987; von Caemmerer, 2000) and $f=0.15$ corrects for the spectral quality of light (Evans, 1987). The dependence of $J$ on temperature is introduced by the relationship of $J_{max}$ with temperature. Here, several models have been proposed (Farquhar *et al.*, 1980; Leuning, 2002; Bunce, 2008; Ali *et al.*, 2015) and applied for *A. thaliana* Col-0 using a reference value of $J_{max}^{ref}=138.5 \mu mol m^{-2}s^{-1}$ (Gandin *et al.*, 2012) (Fig. S10). The model selected for the simulations constitutes the relationship of $J_{max}$ with temperature across multiple plant species (Leuning, 2002), which was found to be a good consensus between the tested models.

To finally add the derived constraints to the metabolic model, the units of these parameters were transformed to match the units of the metabolic model (i.e., $\mu mol$ to $mmol$, $s^{-1}$ to $h^{-1}$, and $m^{-2}$ to $gDW^{-1}$ using LMA). The dependence of LMA on temperature was modeled by a sigmoid function using data from different *A. thaliana* accessions at temperatures between 10 °C and 25 °C (Flexas *et al.*, 2007; Pyl *et al.*, 2012; Pons, 2012; von Caemmerer & Evans, 2015). Despite showing higher RMSE, the sigmoid function was chosen over the Gaussian function because it results in more conservative results towards higher temperatures. A comparison of functions is shown in Fig. S3.

## Optimization problem for prediction of temperature-dependent relative growth rate

Stoichiometric models, such as the AraCore model, are frequently used to predict growth rates and reaction fluxes. Flux balance analysis (FBA) allows the prediction of steady state fluxes by solving a linear optimization problem with the objective of maximizing the production of known biomass precursors with their respective molar fractions per gram dry weight. As a result, one obtains the growth rate ($h^{-1}$) assuming that dry weight is increasing exponentially.

The AraCore model was extended to include protein constraints as well as constraints on photosynthesis and CO_2_ uptake, resulting in the following optimization problem with one quadratic constraint, due to Eq. (S29), as indicated below:

$$\max_{v,A,Z,e} v_{bio}$$

s.t. (subject to)

|  | $\mathbf{Nv=0}$ | (S42) |
| --- | --- | --- |
|  | $\boldsymbol{0\leq v\leq}\mathbf{v}^{\mathbf{max}}$ | (S43) |
|  | $e_{m}-\sum_{i\in GPR\left( m \right)} \frac{v_{i}}{k_{cat}^{m,i}}=0, \forall m\in P$ | (S44) |
|  | $\sum_{m \in\bigcup_{i} GPR\left( i \right)} e_{m}\cdot\mathrm{MW}_{m}\leq\sigma\cdot f\cdot P_{tot}$ |  |
|  | $\boldsymbol{0\leq e\leq}\mathbf{e}^{\mathbf{max}}$ | (S45) |
|  | $A=v_{c}-\alpha v_{o}-v_{{exCO}_{2}}^{mito}$ |  |
|  | $\left( 1-\tau\right)\phi v_{c}\leq v_{o}\leq\left( 1+\tau\right)\phi v_{c}$ |  |
|  | $Z=\frac{{c_{3}C}_{a}-A}{c_{4}}$ |  |
|  | $v_{c}K_{c}+v_{c}Z\left( 1+c_{5} \right)-v_{c}A\left( \frac{1}{g_{m}}+\frac{c_{5}}{g_{m}} \right)-ZV_{cmax}+A\frac{V_{cmax}}{g_{m}}\leq0$ | (quadratic) |
|  | $v_{c}-\alpha v_{o}\leq\frac{J\left( 1-Y \right)}{4+8Y}$ |  |
|  | $\tau=0.001 .$ |  |

The constraints in Eqs. (S42) and (S43) ensure metabolic steady state and upper limits for the flux through the reactions, respectively; Eq. (S44) denotes the enzyme mass balance constraints from the GECKO formalism (Domenzain *et al.*, 2022), and Eq. (S45) provides lower and upper bounds for each protein abundance ([0, Inf)). The calculated value for $f$ was 0.42. In the simulations, the values for $f$ and $\sigma$ were both set to one, to avoid predictions of relative growth rate of low magnitude.

In a second optimization step, we performed parsimonious FBA, whereby the sum of all reaction fluxes was minimized, while keeping $v_{bio}$ at the optimum from solving the problem above. This involved adding a new constraint: $v_{bio}\geq0.99*v_{bio}^{*}$ and changing the objective to $\min\left\| \mathbf{v} \right\|_{1}$. Since the $k_{cat}$ adjustment functionality of the GECKO toolbox was not able to provide corrections of $k_{cat}$ values that result in appropriate growth rate predictions (the relative difference between prediction and measurement was 95.48%). To remedy this issue, all $k_{cat}$ values were multiplied with a factor of 20. This allowed us to arrive at predicted net assimilation rates ($I=800 \mu mol m^{-2}s^{-1}$, $p\left( CO_{2} \right)=380 \mu mol$ (Weston *et al.*, 2011)), that reach the values predicted by the FvCB model and relative growth rates that are only one order of magnitude below the measured relative growth rates (Fig. 2a). The upper bounds for the photon import reaction (Im_hnu) and the CO_2_ import reaction (Im_CO2) were set to ${10}^{4} mmol gDW^{-1}h^{-1}$ to avoid any limitation that may arise from using the default bound of ${10}^{3} mmol gDW^{-1}h^{-1}$. Note that, as shown on Fig. 2a in the main text, the predictions show qualitatively good agreement with measured growth rates.

## Flux variability analysis and flux sampling

The flux distribution that results from the application of a specific constraint set can be described by (*i*) predicting the minimum and maximum possible flux values for each reaction (flux variability analysis (FVA) (Mahadevan & Schilling, 2003)) and (*ii*) sampling random flux vectors to arrive at a probability distribution for the flux of each reaction. FVA can be performed with and without enforcing a minimum percentage of a previously obtained objective value, resulting in operational and feasible ranges, respectively. To predict all operational ranges, we determined the optimal relative growth rate. Next, the minimum sum of fluxes ($\Lambda$) was determined by minimizing the first norm and fixed as an additional constraint. Subsequently, the flux through each reaction in the model was minimized and maximized while ensuring 90% of the optimal objective value ($v_{bio}^{*}$, i.e., relative growth rate):

$$\forall i\in R,\min_{v,A,Z,e} /\max_{v,A,Z,e} v_{i}$$

s.t.

|  | $\mathbf{Nv=0}$ |  |
| --- | --- | --- |
|  | $\boldsymbol{0\leq v\leq}\mathbf{v}^{\mathbf{max}}$ |  |
|  | $v_{bio}\geq0.9\cdot v_{bio}^{*}$ |  |
|  | $\left\Vert\mathbf{v} \right\Vert_{1}\leq\Lambda$ |  |
|  | $e_{m}-\sum_{i\in GPR\left( m \right)} \frac{v_{i}}{k_{cat}^{m,i}}=0, \forall m\in P$ |  |
|  | $\sum_{m \in\bigcup_{i} GPR\left( i \right)} e_{m}\cdot MW_{m}\leq\sigma\cdot f\cdot P_{tot}$ |  |
|  | $\boldsymbol{0\leq e\leq}\mathbf{e}^{\mathbf{max}}$ |  |
|  | $A=v_{c}-\alpha v_{o}-v_{{exCO}_{2}}^{mito}$ |  |
|  | $\left( 1-\tau\right)\phi v_{c}\leq v_{o}\leq\left( 1+\tau\right)\phi v_{c}$ |  |
|  | $Z=\frac{{c_{3}C}_{a}-A}{c_{4}}$ |  |
|  | $v_{c}K_{c}+v_{c}Z\left( 1+c_{5} \right)-v_{c}A\left( \frac{1}{g_{m}}+\frac{c_{5}}{g_{m}} \right)-ZV_{cmax}+A\frac{V_{cmax}}{g_{m}}\leq0$ |  |
|  | $v_{c}-\alpha v_{o}\leq\frac{J\left( 1-Y \right)}{4+8Y}$ |  |
|  | $\tau=0.001 .$ |  |

Next, flux sampling was performed by projecting 30,000 random flux vectors ($\mathbf{v}^{\mathbf{rand}}$) onto the flux space, within the operational ranges. This was performed by minimizing the absolute distance between the predicted reaction fluxes and $\mathbf{v}^{\mathbf{rand}}$, weighted by the maximum of the respective operational ranges ($\mathbf{v}^{\mathbf{max}})$, which were obtained by FVA. In each sampling iteration, 1% of the reactions was randomly chosen for the minimization objective ($R^{select}$).

$$\min_{v,A,Z,e,\delta^{+},\delta^{-}} \sum_{i\in R^{select}} \frac{\left| v_{i}-v_{i}^{rand} \right|}{v_{i}^{max}}=\sum_{i\in R^{select}} \frac{{|\delta}_{i}|}{v_{i}^{max}}=\sum_{i\in R^{select}} \frac{\delta_{i}^{+}+\delta_{i}^{-}}{v_{i}^{max}}$$

s.t.

|  | $\mathbf{Nv=0}$ |  |
| --- | --- | --- |
|  | $\boldsymbol{0\leq v\leq}\mathbf{v}^{\mathbf{max}}$ |  |
|  | $v_{bio}\geq0.9\cdot v_{bio}^{*}$ |  |
|  | $\left\Vert\mathbf{v} \right\Vert_{1}\leq\Lambda$ |  |
|  | $\mathbf{v-}\mathbf{v}^{\mathbf{rand}}\mathbf{=}\boldsymbol{\delta}^{\mathbf{+}}\mathbf{-}\boldsymbol{\delta}^{\mathbf{-}}$ | (S46) |
|  | $\boldsymbol{\delta}^{\boldsymbol{+}}\boldsymbol{,}\boldsymbol{\delta}^{\boldsymbol{-}}\boldsymbol{\geq}\boldsymbol{0}$ |  |
|  | $e_{m}-\sum_{i\in GPR\left( m \right)} \frac{v_{i}}{k_{cat}^{m,i}}=0, \forall m\in P$ |  |
|  | $\sum_{m \in\bigcup_{i} GPR\left( i \right)} e_{m}\cdot MW_{m}\leq\sigma\cdot f\cdot P_{tot}$ |  |
|  | $\boldsymbol{0\leq e\leq}\mathbf{e}^{\mathbf{max}}$ |  |
|  | $A=v_{c}-\alpha v_{o}-v_{{exCO}_{2}}^{mito}$ |  |
|  | $\left( 1-\tau\right)\phi v_{c}\leq v_{o}\leq\left( 1+\tau\right)\phi v_{c}$ |  |
|  | $Z=\frac{{c_{3}C}_{a}-A}{c_{4}}$ |  |
|  | $v_{c}K_{c}+v_{c}Z\left( 1+c_{5} \right)-v_{c}A\left( \frac{1}{g_{m}}+\frac{c_{5}}{g_{m}} \right)-ZV_{cmax}+A\frac{V_{cmax}}{g_{m}}\leq0$ |  |
|  | $v_{c}-\alpha v_{o}\leq\frac{J\left( 1-Y \right)}{4+8Y}$ |  |
|  | $\tau=0.001 .$ |  |

Here, $R$ and $P$ denote the sets of reactions and proteins in the ecAraCore model, respectively. To allow the minimization of the absolute distance between $\mathbf{v}$ and $\mathbf{v}^{\mathbf{rand}}$ using a linear objective, the distance $\delta_{i}$ between each pair of predicted and random fluxes was split into a positive and a negative component (i.e., $\delta^{+}$and $\delta^{-}$, Eq. (S46)), which are both greater or equal to zero. Both FVA and sampling were performed with a light intensity of $I=150 \mu mol m^{-2}s^{-1}$ and $p\left( CO_{2} \right)=380 \mu bar$. All flux samplings (n=30,000) reached a coverage of at least 81.6% with an average of 83.2%. Coverage ($c$) was calculated by the following formula (Binns *et al.*, 2015):

| $c=1-\frac{1}{\left\vert R \right\vert}\sum_{i=1}^{\left\vert R \right\vert} \frac{\max\left( gaps\left( v_{i} \right) \right)}{v_{i}^{max}-v_{i}^{min}}.$ | (S47) |
| --- | --- |

The gaps for each reaction, $gaps\left( v_{i} \right)$, is determined by the intervals between each two consecutive sampled fluxes.

# SI Figures


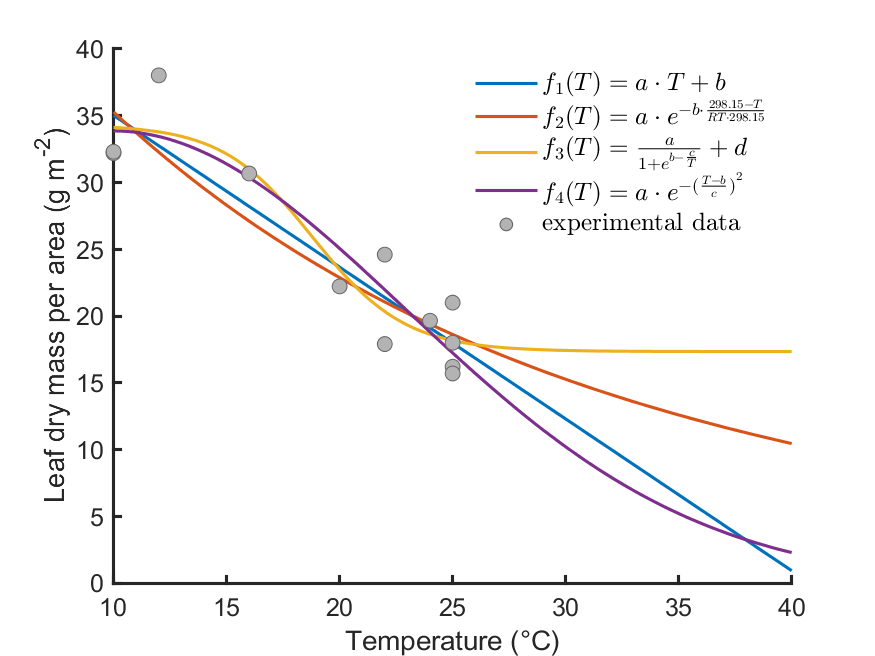


**Fig. S3. Function fits to leaf dry mass per area measured at different temperatures.** The experimental data for *A. thaliana* originate from different experiments with comparable growth conditions (Table S2). RMSE (root mean squared error) values (g/m^2^) for the four functions used are f_1_: 3.026; f_2_: 3.280; f_3_: 2.953; f_4_: 2.919. T: temperature


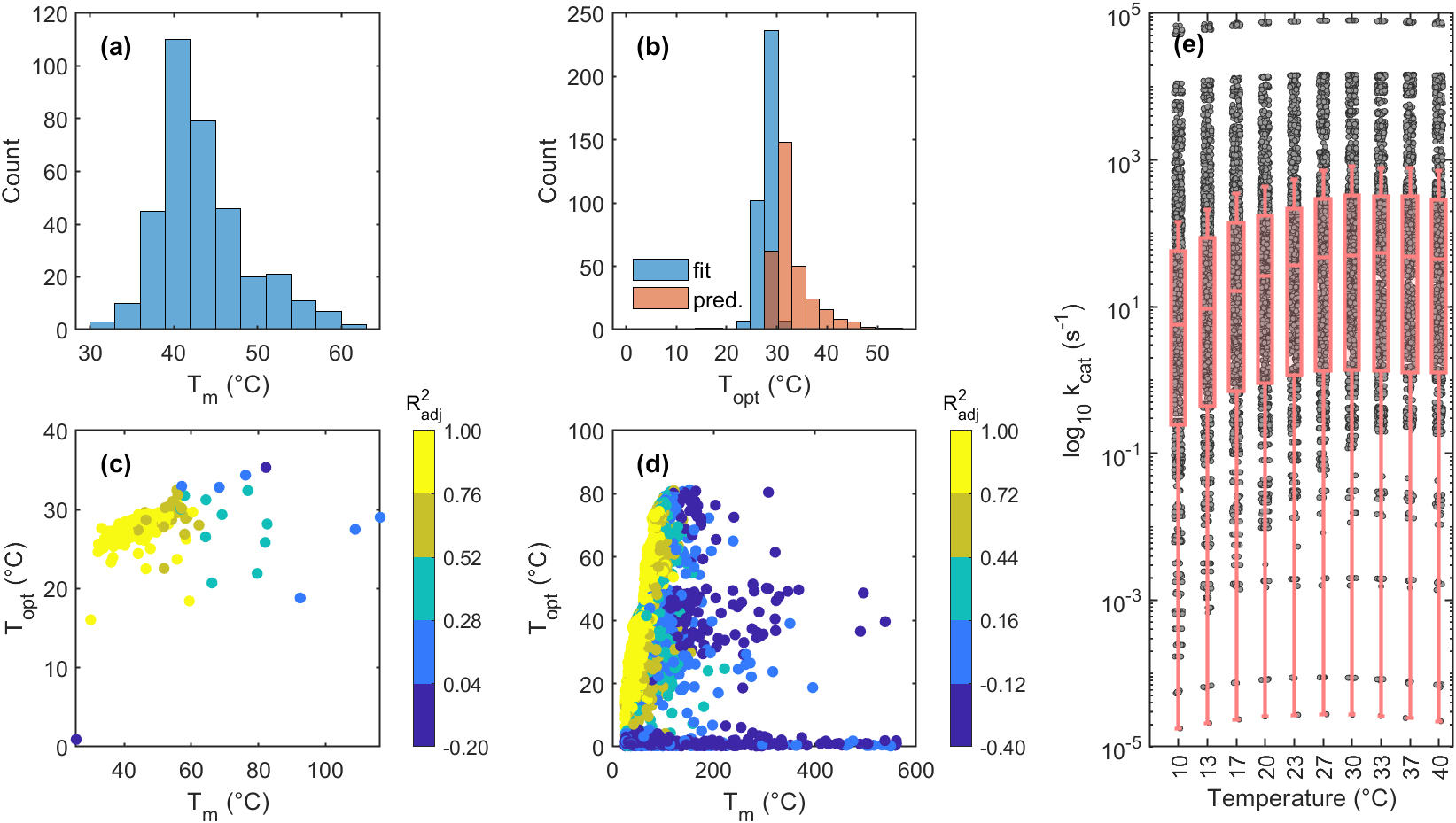


**Fig. S4. Key temperatures of thermostability and adjusted** $\boldsymbol{k}_{\boldsymbol{cat}}$ **values.** Distribution of **(a)** melting temperature (T_m_) and **(b)** optimal temperature (T_opt_) for 354 and 672 proteins contained in the ecAraCore model, respectively. These parameters were obtained by fitting the modified beta growth function (Yin *et al.*, 2003) to thermal protein profiling data for *A. thaliana* from the Meltome Atlas (Jarząb *et al.*, 2020) (cf. Methods). Panel (b) also includes the distribution of predicted T_opt_, as obtained by a Random Forest regression model (cf. Methods, Methods S1). **(c)** Relationship between T_m_ and T_opt_ of proteins in the ecAraCore model, colored by fit quality (adjusted R^2^). **(d)** Relationship between T_m_ and T_opt_ across all proteins in the Meltome Atlas, colored by fit quality (adjusted R^2^). **(e)** Distribution of log-scaled $k_{cat}$ values in the ecAraCore model, adjusted to different temperatures using MMRT (Hobbs *et al.*, 2013). The lines in the boxplots denote the median values, the edges of the box represent the 25% and 75% percentiles of the distribution. The whiskers extend to 1.5 times the interquartile range or the maximum of minimum values, starting from the top or bottom edge of the box.


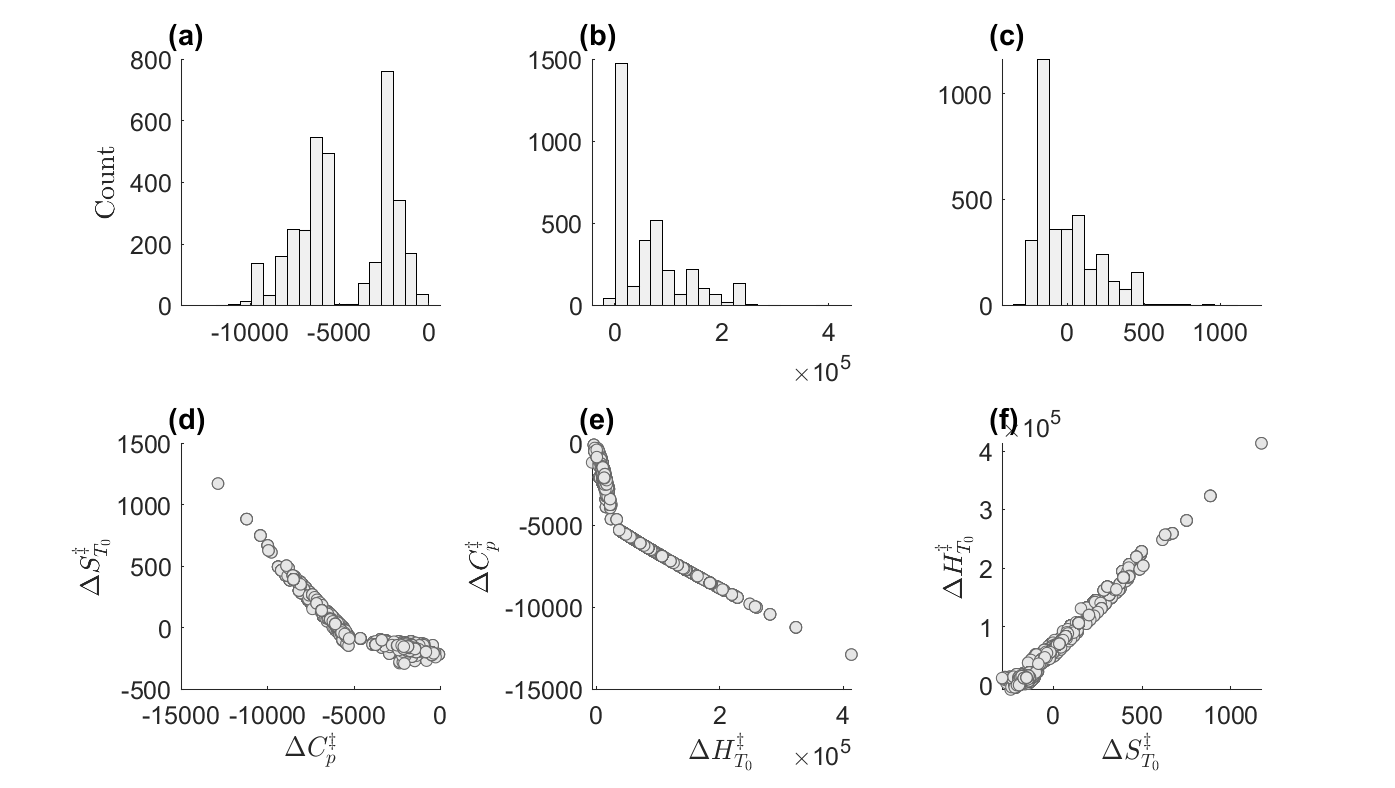


**Fig. S5. Fitted parameters of the MMRT function to describe the relationship between** $\boldsymbol{k}_{\boldsymbol{cat}}$ **and temperature.** The three-parameter function from MMRT (Hobbs *et al.*, 2013) was fitted using key temperatures of protein thermostability (cf. Methods). Panels **(a-c)** show the distribution of the changes in heat capacity ($\Delta C_{p}^{\ddagger}$), enthalpy ($\Delta H_{T_{0}}^{\ddagger}$), and entropy ($\Delta S_{T_{0}}^{\ddagger}$) (n=3405). The pairwise relationships between the parameters are depicted in panels (**d-f**).


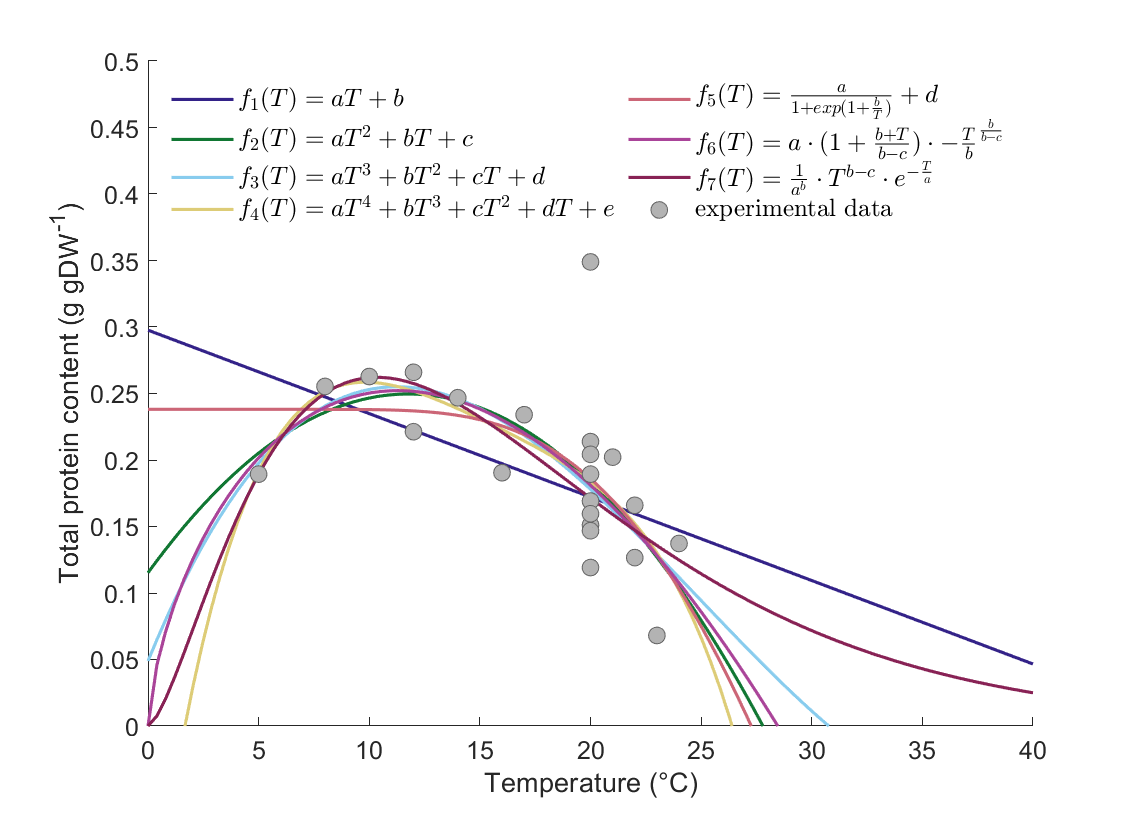


**Fig. S6. Function fits to the total protein content of *A. thaliana* at different temperatures.** The experimental data originate from different experiments with comparable growth conditions (Dataset S2). RMSE (root mean squared error) values (g/gDW) for the seven functions are f_1_: 0.044; f_2_: 0.031; f_3_: 0.032; f_4_: 0.033; f_5_: 0.035; f_6_: 0.030; f_7_: 0.031. gDW: gram dry weight, T: temperature.


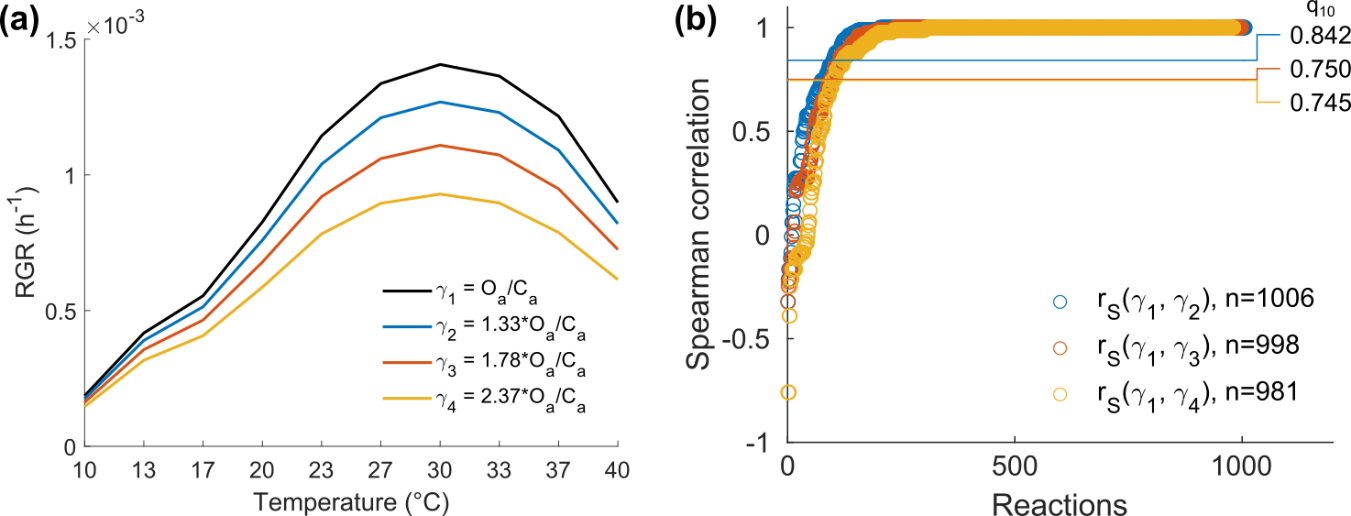


**Fig. S7. Influence of parameter** $\boldsymbol{\gamma}$ **on the predicted RGR and flux distributions.** The ratio between $O_{c}$ and $C_{c}$ is modeled by the parameter $\gamma$. The predictions shown in the Results were performed using $\gamma_{1}=O_{a}C_{a}^{-1}$. Additionally, three alternative values four $\gamma$ were tested to investigate its impact on the predictions: $\gamma_{2\ldots4}:\left[ \frac{O_{a}}{C_{i}}, \frac{O_{a}}{C_{c}}, \frac{O_{a}}{0.75C_{c}} \right]$, assuming that $C_{i}\approx0.75C_{a}$ and $C_{c}\approx0.75C_{i}$. **(a)** Predicted RGR temperature responses with different $\gamma$ values. **(b)** Spearman correlation between the predicted temperature responses per reaction flux in the flux distributions corresponding to the RGR values in (a). The correlations for the reactions were sorted ascendingly. While the ecAraCore model contained overall 2509 reactions, not all correlations could be calculated due to reactions carrying zero flux across (almost) all temperatures. The exact numbers of reactions per comparison are given in the legend. Moreover, to illustrate the high correspondence, the 10% quantiles (q_10_) of the correlations per comparison are indicated by horizontal lines in the respective color. RGR: relative growth rate, O_a_ and O_c_: ambient and chloroplastic partial pressures of O_2_, C_a_ and C_c_: ambient and chloroplastic partial pressures of CO_2_


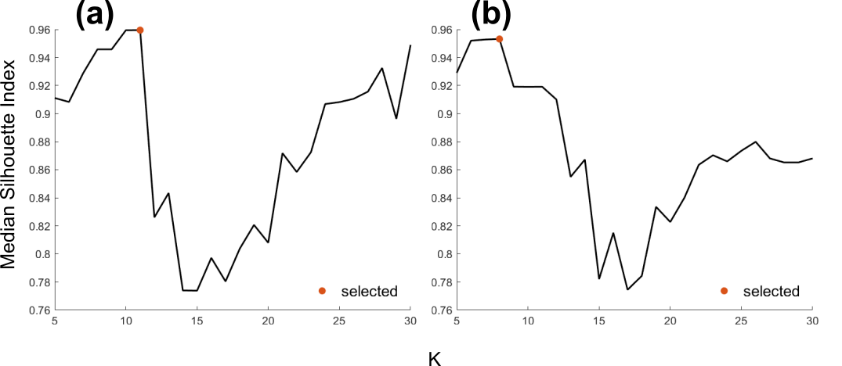


**Fig. S8. Silhouette Index of K-medoids clustering with different cluster numbers**. Clustering of predicted growth responses of *A. thaliana* to supplementation with light intensities of **(a)** $I=150 \mu mol m^{-2}s^{-1}$ and **(b)** $I=400 \mu mol m^{-2}s^{-1}$, respectively (Figs. S19 and S20). The selected number of clusters is indicated by the orange dot. I: irradiance


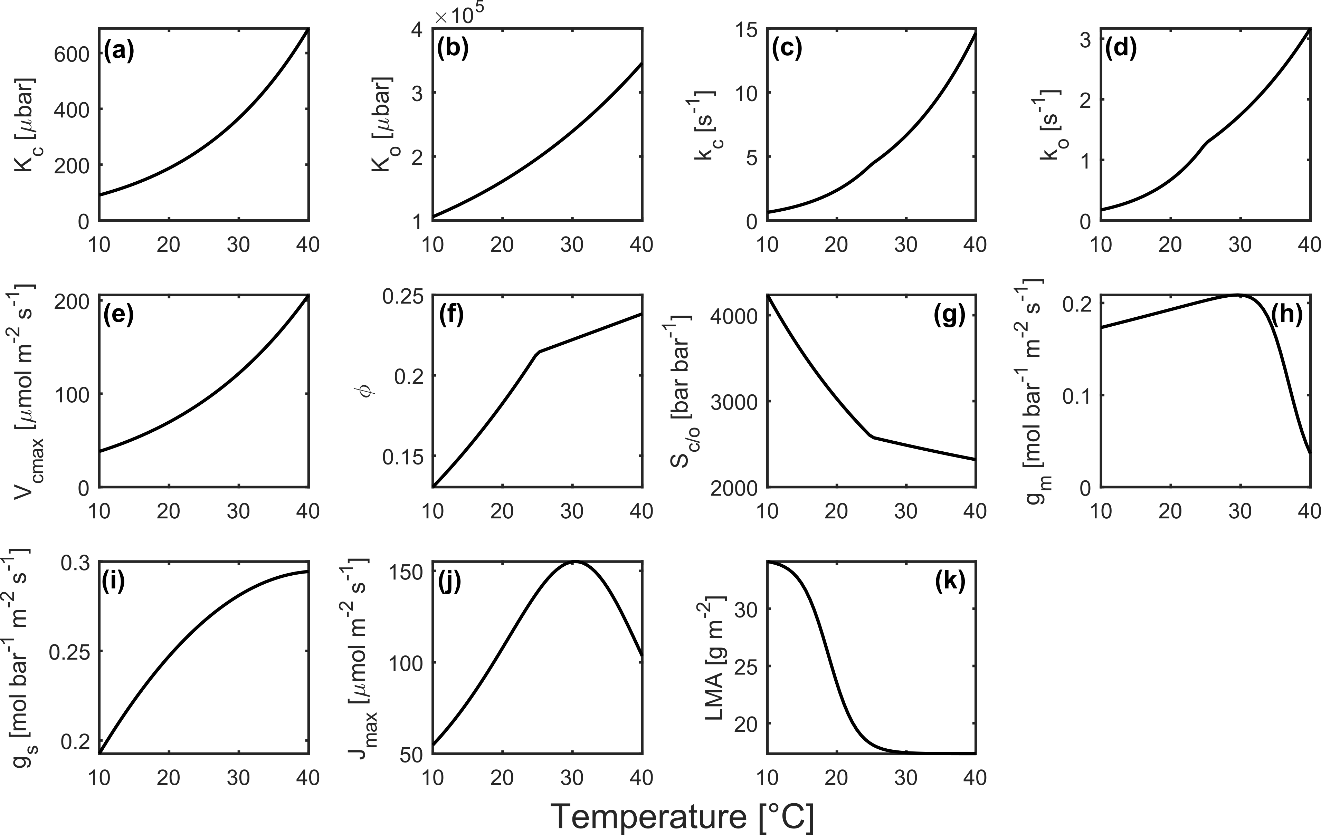


**Fig. S9. Temperature dependence of the FvCB model parameters.** The temperature-dependent parameters are specific to *A. thaliana* and include **(a)** Michaelis-Menten value (K_M_) of the RuBisCO enzyme for CO_2_ (K_c_), **(b)** K_M_ value of the of the RuBisCO enzyme for O_2_ (K_o_), **(c)** k_cat_ value of the RuBisCO enzyme with CO_2_ as a substrate (k_c_), **(d)** k_cat_ value of the RuBisCO enzyme with O_2_ as a substrate (k_o_), **(e)** maximum velocity of the RuBisCO carboxylation reaction (V_cmax_), **(f)** ratio between the oxygenation and carboxylation reactions catalyzed by RuBisCO (φ), **(g)** specificity of RuBisCO for CO_2_ over O_2_ (S_c/o_), **(h)** mesophyll conductance (g_m_), **(i)** stomatal conductance (g_s_), **(j)** light saturated potential rate of electron transport (J_max_), and **(k)** leaf mass per area (LMA).


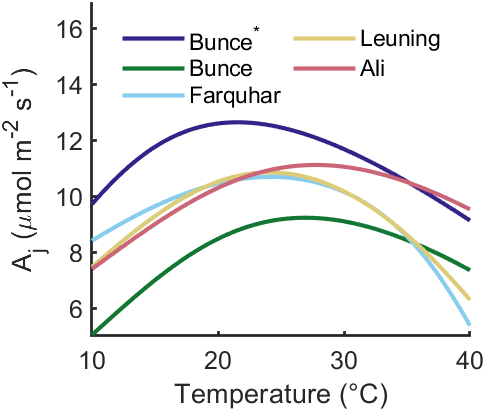


**Fig. S10. Prediction of electron transport-limited net CO_2_ assimilation rate (**$\boldsymbol{A}_{\boldsymbol{j}}$**) with different temperature models for** $\boldsymbol{J}_{\boldsymbol{max}}$**.** Five temperature models (Farquhar *et al.*, 1980; Leuning, 2002; Bunce, 2008; Ali *et al.*, 2015) for the light saturated potential rate of electron transport ($J_{max}$) were compared with a reference value of $J_{max}=138.5 \mu mol m^{-2} s^{-1}$ (Gandin *et al.*, 2012), except for “Bunce*”, where a value of $J_{max}=288 \mu mol m^{-2}s^{-1}$ was used (Bunce, 2008).


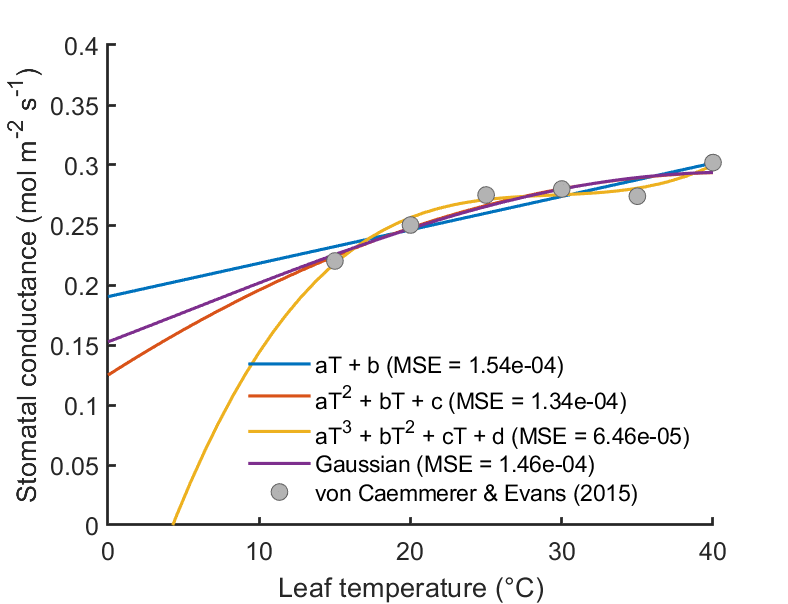


**Fig. S11. Comparison of different temperature for stomatal conductance (**$\boldsymbol{g}_{\boldsymbol{s}}$**) in *A. thaliana*.** The equation of the Gaussian model was $g_{s}\left( T \right)=a\cdot e^{-\left( \frac{T-b}{c} \right)^{2}}$. The data points shown in grey originate were extracted from Fig. 1 of the original publication (von Caemmerer & Evans, 2015) using WebPlotDigitizer version 4.6 (<https://automeris.io/WebPlotDigitizer>). T: temperature, $g_{s}$: stomatal conductance, MSE: mean squared error


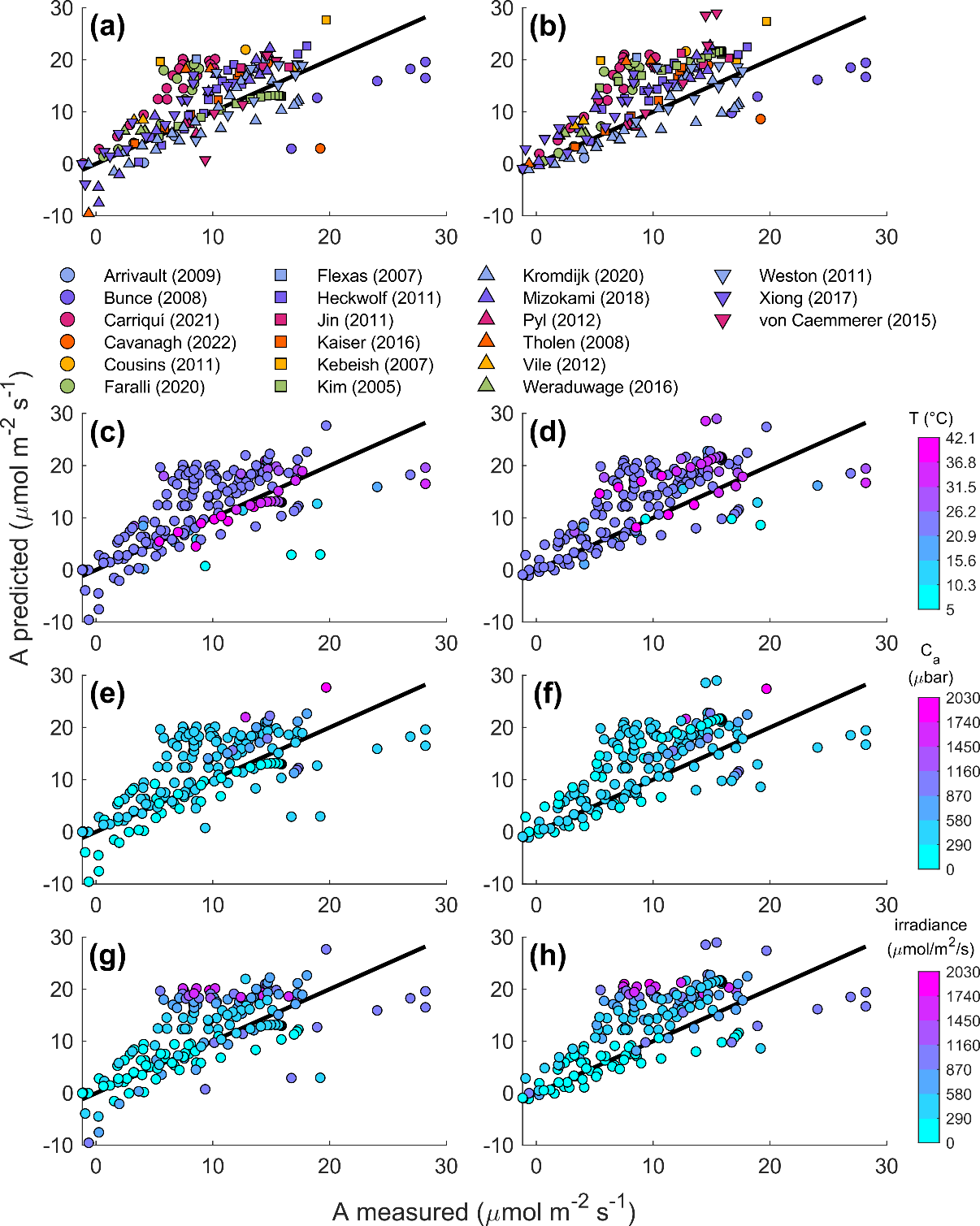
**Fig. S12. Correlation of predicted and measured net CO_2_ assimilation rate (**$\boldsymbol{A}$**) under different conditions.** $A$ was predicted using the FvCB and ecAraCore model using temperature, ambient (C_a_, ecAraCore) or intercellular (C_i_, FvCB model) partial pressure of CO_2_, ambient O_2_ partial pressure, and light intensity (irradiance, I) from multiple experiments with *A. thaliana* Col-0 as inputs. Missing data on C_i_ were imputed by multiplying C_a_ with 0.75. The Pearson correlations ($r$) of the ecAraCore model and FvCB model with the experimental data were 0.66 ($P=1.3\cdot{10}^{-23}$) and 0.70 ($P=2.5\cdot{10}^{-27}$), respectively. The predictions from both models were color-coded by **(a,b)** the study (first author (year)), as well as **(c,d)** the temperature (the data shown in (c) are identical to Fig. 2b), **(e,f)** ambient p(CO_2_), and **(g,h)** light intensity used in the experiment. T: temperature, FvCB: Farquar-von Caemmerer-Berry model (Farquhar *et al.*, 1980)


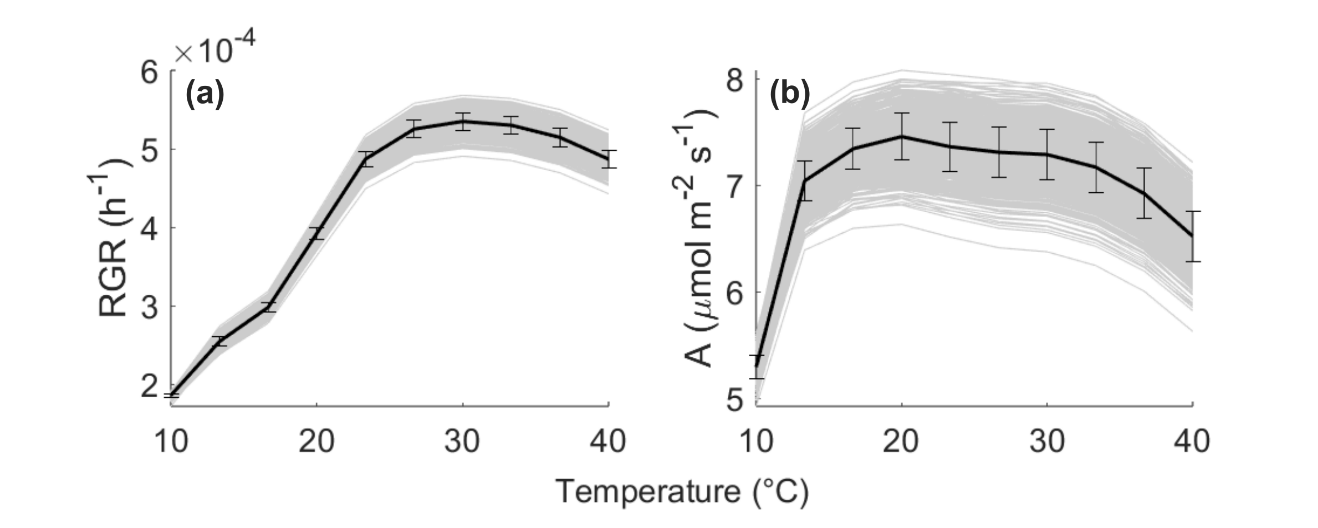


**Fig. S13. Robustness analysis of the temperature-dependent ecAraCore model.** To assess the robustness of the model’s predictions of *A. thaliana*’s temperature responses in relative growth rate (RGR) **(a)** and $A$ **(b)**, 1000 random parameter samples were drawn from log-normal distributions for $V_{cmax}, K_{c}, K_{o}, k_{c}, k_{o}$, and $J_{max}$. The standard deviation was assumed to be 5% of the respective parameter value. The gray lines show the predicted temperature responses with the sampled parameter combinations. The black line represents the predictions with the original model parameters. The error bars show standard deviation from the predictions with the sampled parameter sets. $V_{cmax}$: maximum velocity of the RuBisCO carboxylation reaction, $K_{c}$: Michaelis-Menten value (K_M_) of the RuBisCO enzyme for CO_2_, $K_{o}$: K_M_ value of the of the RuBisCO enzyme for O_2_ (K_o_), $k_{c}$: k_cat_ value of the RuBisCO enzyme with CO_2_ as a substrate, $k_{o}$: k_cat_ value of the RuBisCO enzyme with O_2_ as a substrate, $J_{max}$: light-saturated potential rate of electron transport


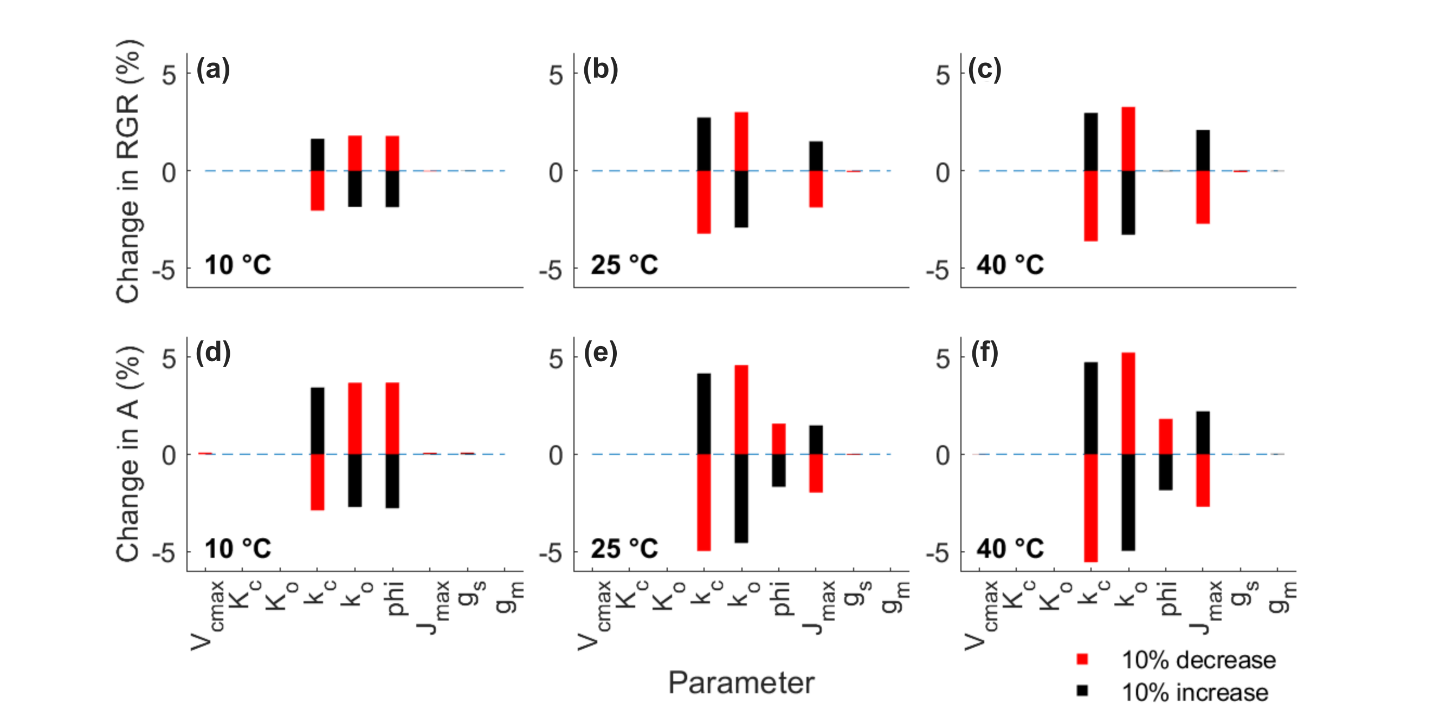


**Fig. S14. Sensitivity analysis for the temperature-dependent ecAraCore model.** The values nine model parameters were increased (black) and decreased (red) by 10%, respectively, and the change in predicted relative growth rate (RGR) **(a-c)** net CO_2_ assimilation rate ($A$) **(d-f)** were recorded. The simulations were performed at 10 °C (a, d), 25 °C (d, e), and 40 °C (c, f), with a light intensity of $150 \mu mol m^{-2} s^{-1}$ and $C_{a}=400 \mu bar$. $V_{cmax}$: maximum velocity of the RuBisCO carboxylation reaction, $K_{c}$: Michaelis-Menten value (K_M_) of the RuBisCO enzyme for CO_2_, $K_{o}$: K_M_ value of the of the RuBisCO enzyme for O_2_ (K_o_), $k_{c}$: k_cat_ value of the RuBisCO enzyme with CO_2_ as a substrate, $k_{o}$: k_cat_ value of the RuBisCO enzyme with O_2_ as a substrate, phi: ratio between the oxygenation and carboxylation reactions catalyzed by RuBisCO (φ), $J_{max}$: light-saturated potential rate of electron transport, $g_{s}$: stomatal conductance, $g_{m}$: mesophyll conductance


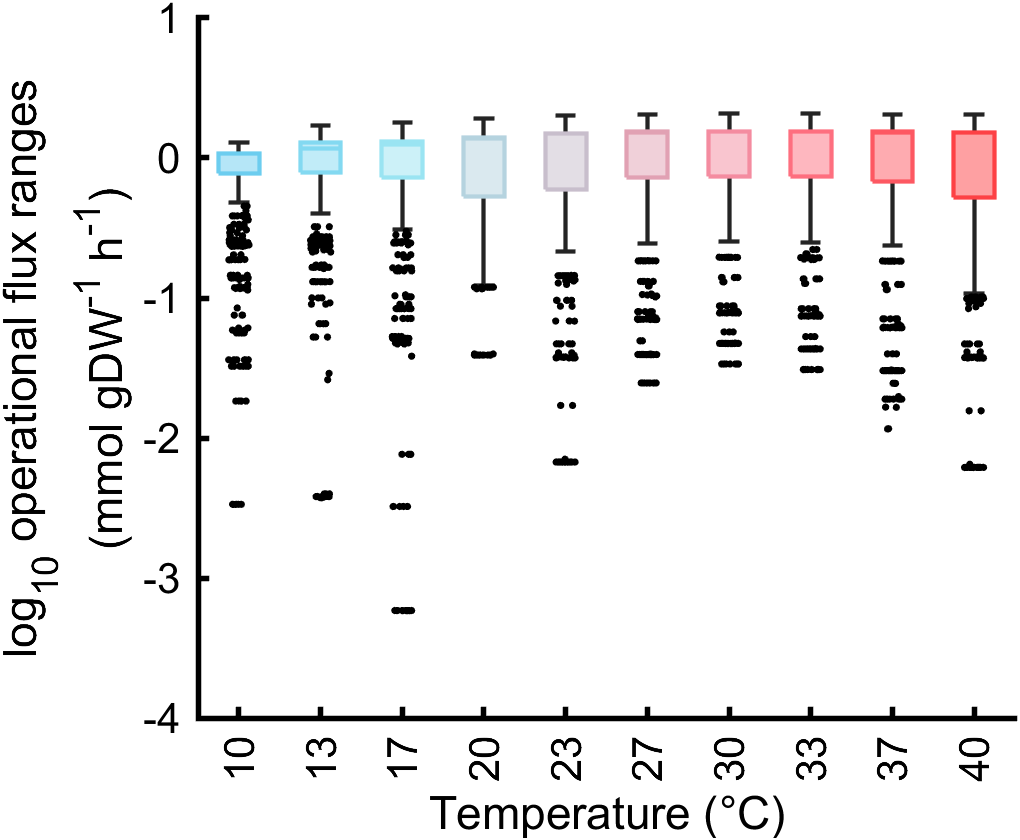


**Fig. S15. Operational flux ranges at different temperatures.** The flux through each reaction in the ecAraCore model was minimized and maximized while guaranteeing at least 90% of the respective optimal relative growth rate at each temperature. Moreover, the sum of fluxes was fixed to the minimum sum of fluxes at the optimal growth rate at a given temperature. Box plots show the interquartile range (IQR), the middle line represents the median, and vertical lines represent whiskers that either extend to 1.5 times the IQR or the minimum or maximum value, respectively. gDW: gram dry weight


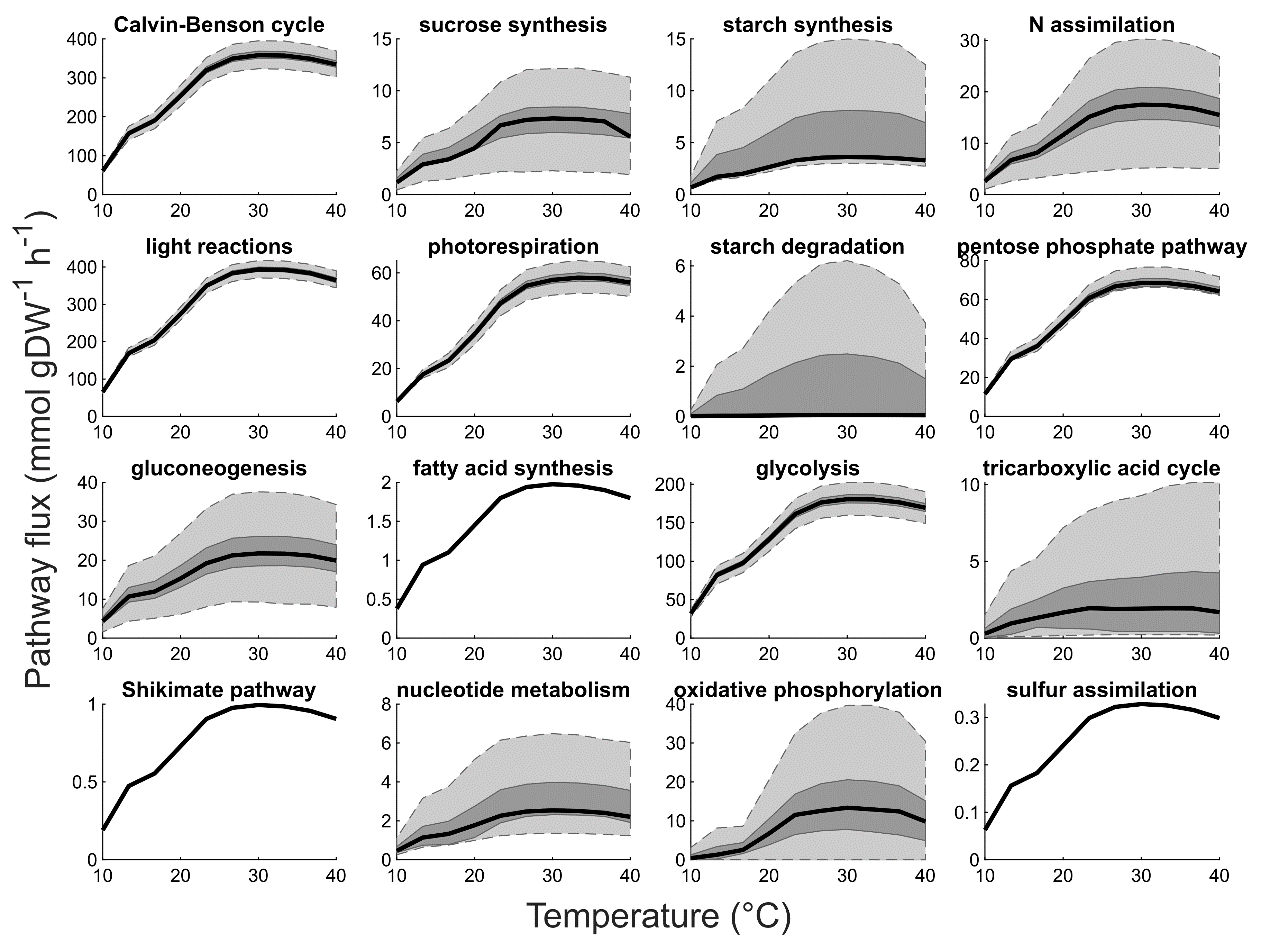


**Fig. S16. Distribution of flux through selected pathways at different temperatures.** Pathway flux at different temperatures was obtained by flux sampling of the ecAraCore model (n=30,000) at 90% of the optimal RGR and minimum total flux through the network, obtained by parsimonious flux balance analysis. The bold black line represents the median, the dark grey area shows the interquartile range, and the lighter grey area mimics whiskers of a boxplot. gDW: gram dry weight


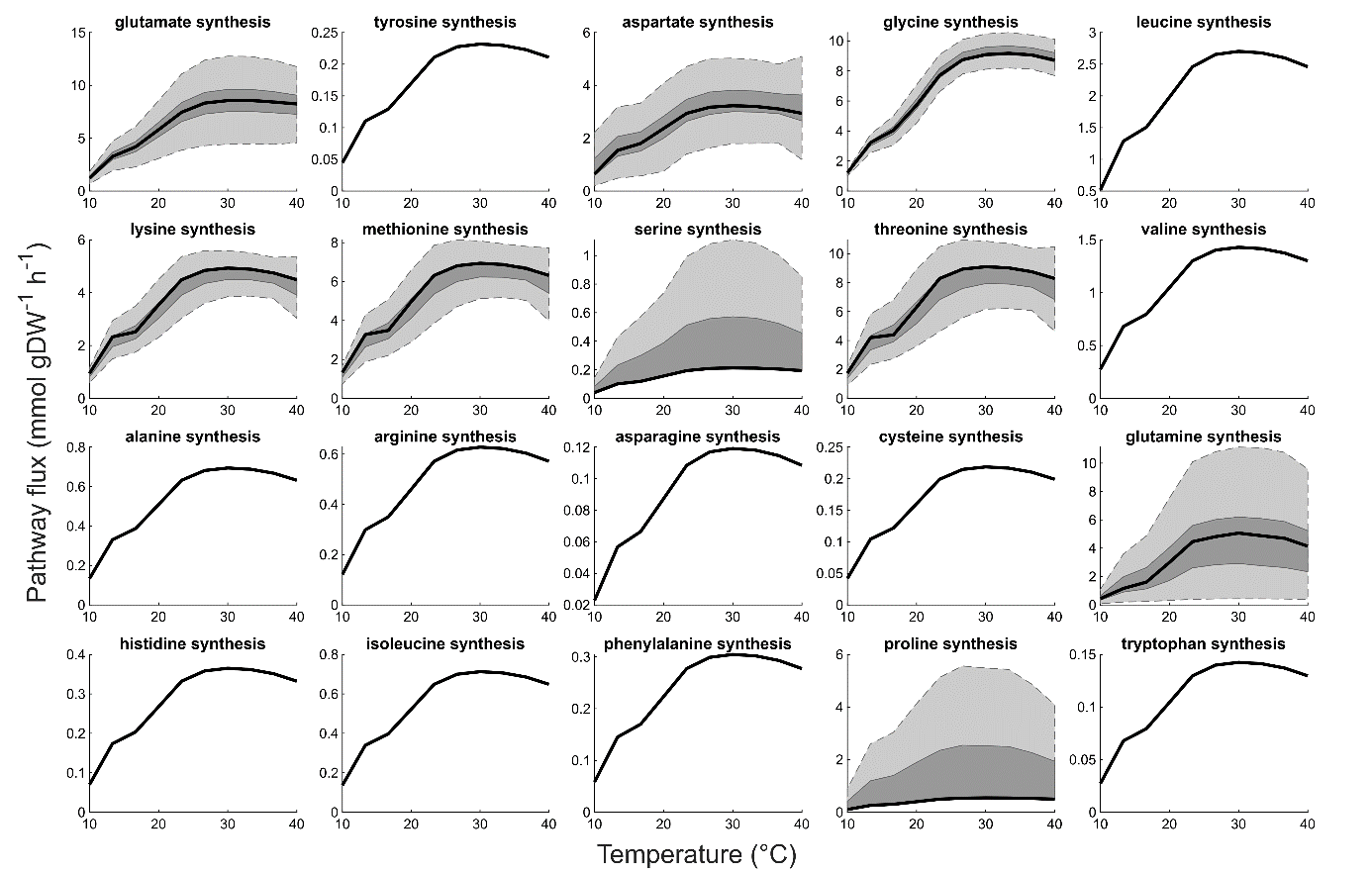


**Fig. S17. Distribution of flux through amino acid synthesis pathways at different temperatures.** Sums of fluxes through selected pathways at different temperatures as obtained by flux sampling of the ecAraCore model (n=30,000) at 90% of the optimal RGR and minimum total flux through the network, obtained by parsimonious flux balance analysis. The bold black line represents the median, the dark grey area shows the interquartile range, and the lighter grey area mimics whiskers of a boxplot. gDW: gram dry weight


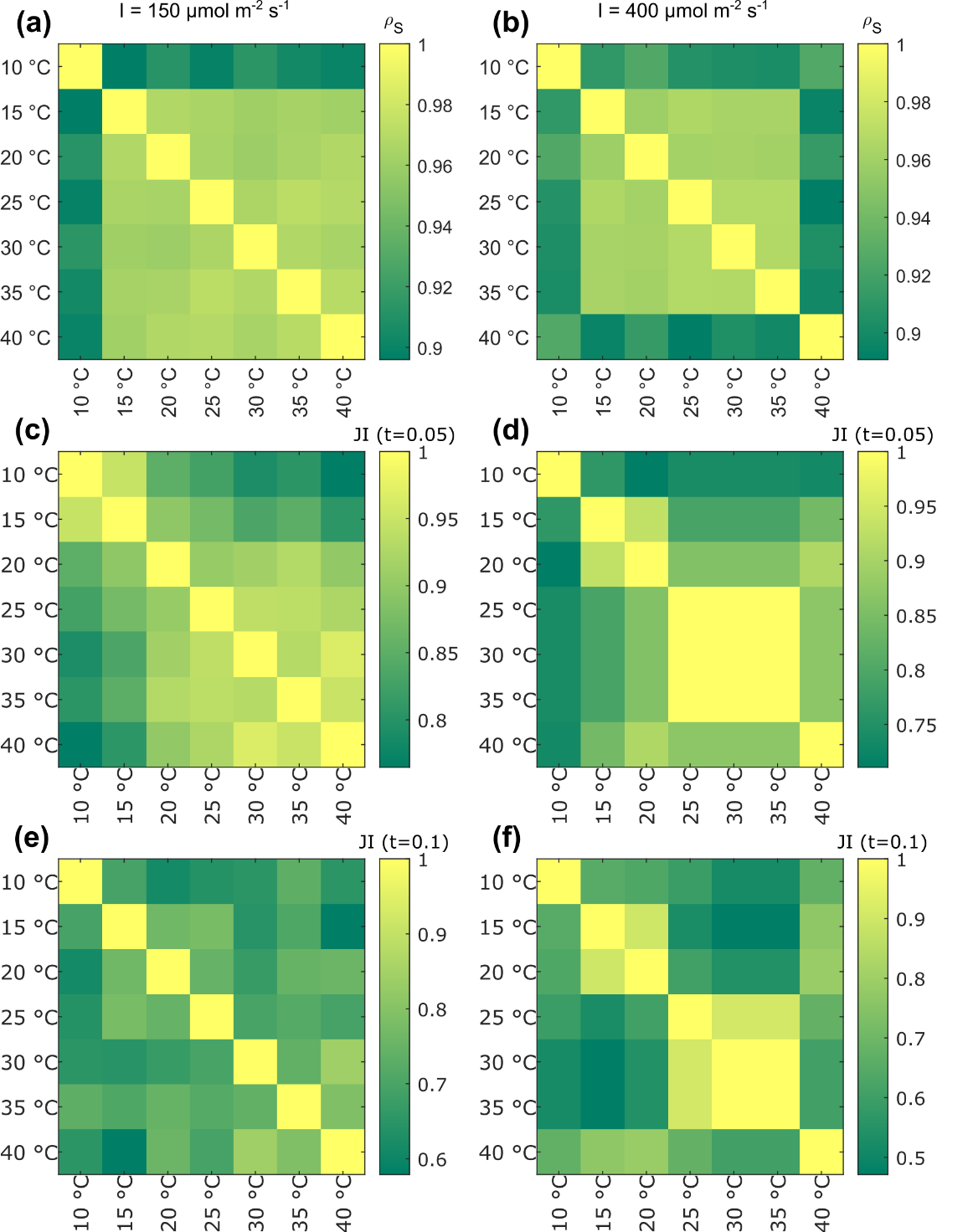


**Fig. S18. Similarity between growth-limiting metabolites found at different temperatures.** For this analysis, an import reaction was added for each metabolite individually and the following change in optimal relative growth rate (RGR) was scored. The change in RGR was scaled to the maximum over all proteins at each temperature. The similarity between limiting metabolites at different temperatures was then quantified by **(a, b)** Spearman correlation ($\rho_{S}$) and **(d-f)** Jaccard index (JI) at two different thresholds using the changes in RGR across all metabolites**.** The analysis was performed with two different light intensities, $I$, (i.e., $150 \mu mol m^{-2}s^{-1}$ [a, c, e] and $400 \mu mol m^{-2}s^{-1}$ [b, d, f]). The complete results from this analysis can be found in Datasets S5 and S6.


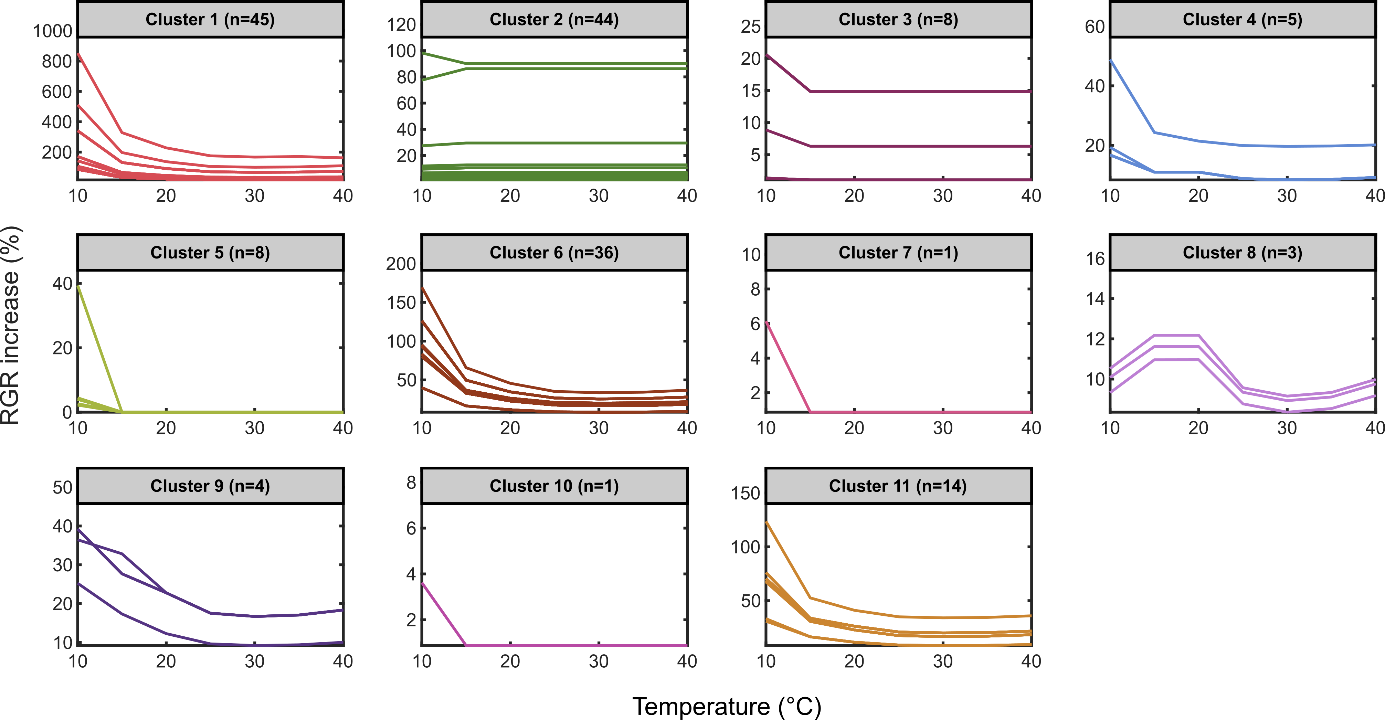


**Fig. S19. K-medoids clustering of predicted growth responses to metabolite supplementation.** Supplementation was simulated by adding an import reaction for each metabolite individually, followed by prediction of the optimal relative growth rate (RGR). All simulations were carried out with a fixed ratio between NH_4_^+^ and NO_3_^-^ uptake fluxes of 1:3 (M’rah Helali *et al.*, 2010) and a light intensity of $I=150 \mu mol m^{-2}s^{-1}$. Metabolites with increases in relative growth rate below 1% were excluded from the clustering. Cosine distance (1-cosine similarity) was used to compute pairwise distances between the temperature responses. The best K was determined by the maximum of median silhouette Index values over all tested K (Fig. S8a and Methods). The complete results from this analysis can be found in Dataset S5.


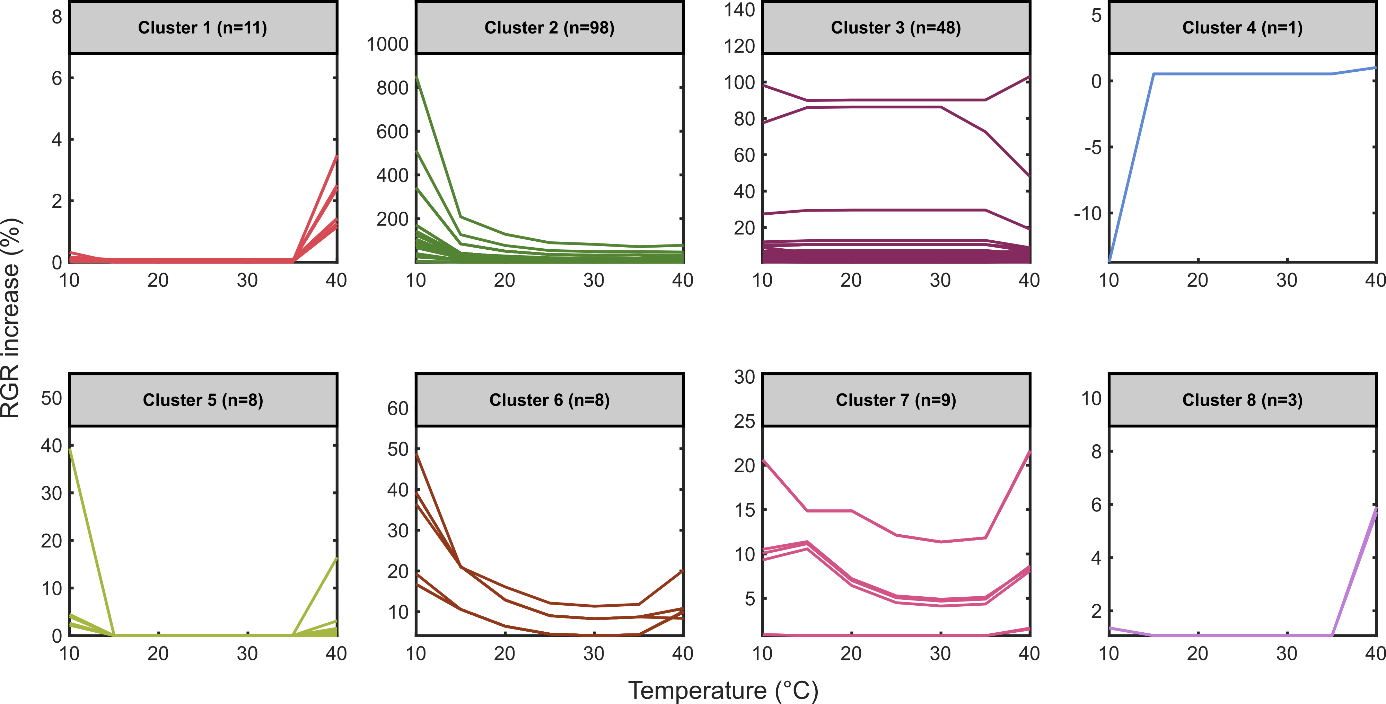


**Fig. S20. K-medoids clustering of predicted growth responses to metabolite supplementation.** Supplementation was simulated by adding an import reaction for each metabolite individually, followed by prediction of the optimal relative growth rate (RGR). All simulations were carried out with a fixed ratio between NH_4_^+^ and NO_3_^-^ uptake fluxes of 1:3 (M’rah Helali *et al.*, 2010) and a light intensity of $I=400 \mu mol m^{-2}s^{-1}$. Metabolites with increases in relative growth rate below 1% were excluded from the clustering. Cosine distance (1-cosine similarity) was used to compute pairwise distances between the temperature responses. The best K was determined by the maximum of median silhouette Index values over all K (Fig. S8b, Methods). The complete results from this analysis can be found in Dataset S6.

# SI Tables

**Table S1. Experimental data and temperature dependences used to parameterize the FvCB model.** Whenever possible, data were collected for *A. thaliana* Col-0. $R$: universal gas constant, $T$: temperature, $E_{a}$: activation energy, $k_{25}$: parameter value at 25 °C, $\Delta H_{a}$: enthalpy change (activation), $\Delta H_{a}$: enthalpy change (deactivation), $T_{0}$: reference temperature, $\Delta S$: entropy change

| parameter | explanation | reference value at 25°C ($\boldsymbol{k}_{\boldsymbol{25}}$) | temperature function |
| --- | --- | --- | --- |
| $\boldsymbol{k}_{\boldsymbol{c}}$ | $k_{cat}$ of RuBisCO carboxylation reaction | $4.4 s^{-1}$ (Walker *et al.*, 2013) | reference (Boyd *et al.*, 2019)  $k_{T}=k_{25}\cdot e^{-\frac{E_{a}\left( 298.15-T \right)}{RT\cdot298.15}}$  $E_{a}\left( 10-25 ^{\circ}C \right)=90360 J mol^{-1}$  $E_{a}\left( 25-40 ^{\circ}C \right)=62200 J mol^{-1}$ |
| $\boldsymbol{k}_{\boldsymbol{o}}$ | $k_{cat}$ of RuBisCO oxygenation reaction | $1.27 s^{-1}$ (Walker *et al.*, 2013) | reference (Boyd *et al.*, 2019)  $k_{T}=k_{25}\cdot e^{-\frac{E_{a}\left( 298.15-T \right)}{RT\cdot298.15}}$  $E_{a}\left( 10-25 ^{\circ}C \right)=92959 J mol^{-1}$  $E_{a}\left( 25-40 ^{\circ}C \right)=47110 J mol^{-1}$ |
| $\boldsymbol{K}_{\boldsymbol{c}}$ | RuBisCO $K_{M}$ value for CO_2_ | $265 \mu bar$ (Walker *et al.*, 2013) | reference (Walker *et al.*, 2013)  $K_{c}\left( T \right)=10\cdot e^{c-\frac{\Delta H_{a}}{RT}}$  $c=23.32$  $\Delta H_{a}=49700 J mol^{-1}$ |
| $\boldsymbol{K}_{\boldsymbol{o}}$ | RuBisCO $K_{M}$ value for O_2_ | $201 mbar$ (Walker *et al.*, 2013) | reference (Walker *et al.*, 2013)  $K_{o}\left( T \right)={10}^{4}\cdot e^{c-\frac{\Delta H_{a}}{RT}}$  $c=14.72$  $\Delta H_{a}=29100 J mol^{-1}$ |
| $\boldsymbol{S}_{\boldsymbol{c}/\boldsymbol{o}}$ | RuBisCO relative specificity for CO_2_ over O_2_ | $\frac{k_{c}}{K_{c}}\cdot\frac{K_{o}}{k_{o}}$ |  |
| $\boldsymbol{V}_{\boldsymbol{cmax}}$ | maximal carboxylation velocity | $96.3 \mu mol m^{-2}s^{-1}$ (Heckwolf *et al.*, 2011) | reference (Walker *et al.*, 2013)  $V_{cmax}\left( T \right)=k_{25}\cdot e^{c-\frac{\Delta H_{a}}{RT}}$  $c=16.66$  $\Delta H_{a}=41400 J mol^{-1}$ |
| $\boldsymbol{J}_{\boldsymbol{max}}$ | light saturated potential rate of electron transport | $138.5 \mu mol m^{-2}s^{-1}$ (Gandin *et al.*, 2012) | reference (Leuning, 2002)  $J_{max}\left( T \right)=k_{25}\cdot C\cdot\frac{e^{\frac{H_{a}}{RT_{0}}\cdot\left( 1-\frac{T_{0}}{T} \right)}}{1+e^{\frac{\left( S_{v}T-H_{d} \right)}{RT}}}$  $C=1+e^{\frac{\left( S_{v}T_{0}-H_{d} \right)}{RT_{0}}}$  $T_{0}=298.15 K$  $H_{a}=50300 J mol^{-1}$  $H_{d}=152044 J mol^{-1}$  $S_{v}=495 J mol^{-1}K^{-1}$  Please refer to Fig. S10 for a comparison of temperature models. |
| $\boldsymbol{g}_{\boldsymbol{m}}$ | mesophyll conductance | $0.2 mol m^{-2}s^{-1}bar^{-1}$ (Flexas *et al.*, 2007) | references (Bernacchi *et al.*, 2002; Walker *et al.*, 2013)  $g_{m}\left( T \right)=k_{25}\cdot\exp\left( \frac{c-\frac{\Delta H_{a}}{RT}}{1+e^{\frac{T\Delta S-\Delta H_{d}}{RT}}} \right)$  $c=3$  $\Delta H_{a}=7400 J mol^{-1}$  $\Delta H_{d}=434000 J mol^{-1}$  $\Delta S=1400 J mol^{-1}K^{-1}$ |
| $\boldsymbol{g}_{\boldsymbol{s}}$ | stomatal conductance | $0.2 mol m^{-2}s^{-1}$ (Heckwolf *et al.*, 2011; Gandin *et al.*, 2012)  (The value from fitted temperature function: $0.27 mol m^{-2}s^{-1}$) | $g_{s}\left( T \right)=\beta_{1}T^{2}+\beta_{2}T+\beta_{3}$  The function was fit to experimental data for *A. thaliana* Col-0 (von Caemmerer & Evans, 2015). Please refer to Fig. S11 for a comparison of temperature modeling functions tested. The quadratic function was preferred over the cubic function because the optimal value represents the global optimum in the quadratic function, instead of a saddle point. |
| $\boldsymbol{r}_{\boldsymbol{b}}$ | boundary layer conductance | $1 m^{2} s mol^{-1}$ (Farquhar & Wong, 1984) |  |
| $\boldsymbol{q}$ | absorptance | $0.85$ (von Caemmerer, 2000) |  |
| $\boldsymbol{f}$ | correction factor for the spectral quality of light | $0.15$ (Evans, 1987) |  |
| $\boldsymbol{\theta}$ | convexity factor for the light dependence of the electron transport rate | $0.7$ (Evans & Terashima, 1987) |  |
| $\boldsymbol{R}_{\boldsymbol{d}}$ | “dark” respiration (only used for FvCB model alone) | $0.86 \mu mol m^{-2}s^{-1}$ (Farquhar *et al.*, 1980) |  |
| $\boldsymbol{\phi}$ | ratio between oxygenation and carboxylation (only used for FvCB model alone) | $0.27$ (Farquhar *et al.*, 1980) |  |
| TPU | Triose phosphate utilization (only used for FvCB model alone) | $8.14 mol m^{-2}s^{-1}$ (Morales *et al.*, 2018) |  |
| $\boldsymbol{E}_{\boldsymbol{a}}\left( \boldsymbol{k}_{\boldsymbol{c}} \right)$ | activation energy of $k_{c}$ (only used for FvCB model alone) | $58520 J mol^{-1}$ (Farquhar *et al.*, 1980) |  |
| $\boldsymbol{E}_{\boldsymbol{a}}\left( \boldsymbol{k}_{\boldsymbol{o}} \right)$ | activation energy of $k_{o}$ (only used for FvCB model alone) | $58520 J mol^{-1}$ (Farquhar *et al.*, 1980) |  |
| $\boldsymbol{E}_{\boldsymbol{a}}\left( \boldsymbol{K}_{\boldsymbol{c}} \right)$ | activation energy of $K_{c}$ (only used for FvCB model alone) | $59356 J mol^{-1}$ (Farquhar *et al.*, 1980) |  |
| $\boldsymbol{E}_{\boldsymbol{a}}\left( \boldsymbol{K}_{\boldsymbol{o}} \right)$ | activation energy of $K_{o}$ (only used for FvCB model alone) | $35948 J mol^{-1}$ (Farquhar *et al.*, 1980) |  |
| $\boldsymbol{E}_{\boldsymbol{a}}\left( \boldsymbol{V}_{\boldsymbol{cmax}} \right)$ | activation energy of $V_{cmax}$ (only used for FvCB model alone) | $58520 J mol^{-1}$ (Farquhar *et al.*, 1980) |  |
| $\boldsymbol{LMA}$ | leaf mass per area | $25 gDW m^{-2}$ (see Table S2) | $LMA\left( T \right)=\frac{\beta_{1}}{1+ e^{\beta_{2}-\frac{\beta_{3}}{T}}}+\beta_{4}$  The function was fit to experimental data from multiple studies (please refer to Table S2 for data and references and Fig. S3 for a comparison of temperature models. |

**Table S2. Experimental data on the leaf mass per area (LMA) at different temperatures, which were used to describe the temperature dependence of LMA.** All data were collected for *A. thaliana*, preferably ecotype Col-0.

| LMA (gDW m^-2^) | T (°C) | Reference |
| --- | --- | --- |
| 18.00 | 25 | (Flexas *et al.*, 2007) |
| 22.22 | 20 | (Hummel *et al.*, 2010) |
| 32.20 | 10 | (Pons, 2012) |
| 24.60 | 22 | (Pons, 2012) |
| 32.30 | 10 | (Pons, 2012) |
| 17.90 | 22 | (Pons, 2012) |
| 38.02 | 12 | (Pyl *et al.*, 2012) |
| 30.67 | 16 | (Pyl *et al.*, 2012) |
| 19.65 | 24 | (Pyl *et al.*, 2012) |
| 21.00 | 25 | (Walker *et al.*, 2013) |
| 16.20 | 25 | (von Caemmerer & Evans, 2015) |
| 15.70 | 25 | (Luo *et al.*, 2021) |

**Table S3. *Arabidopsis thaliana* T-DNA insertion mutant lines assessed for their leaf development at 17 °C.** WT: wild type

| Arabidopsis Gene ID | NASC ID | SALK line identifier |
| --- | --- | --- |
| WT (Columbia-0 CS76778) | N76778 | na |
| AT3G23580 | N657325 | SALK_150365C |
| AT5G08740 | N661996 | SALK_030158C |
| AT3G52930 | N663895 | SALK_124383C |
| AT1G17290 | N655815 | SALK_107662C |
| AT5G38710 | N658847 | SALK_108179C |
| AT4G18440 | N656813 | SALK_100845C |
| AT2G21940 | N661121 | SALK_122662C |
| AT2G02010 | N661068 | SALK_106240C |
| AT5G11520 | N658076 | SALK_008526C |
| AT2G45290 | N659664 | SALK_011139C |
| AT1G22170 | N653346 | SALK_087895C |
| AT1G09795 | N655019 | SALK_024115C |
| AT2G17630 | N660568 | SALK_115392C |
| AT1G62960 | N653411 | SALK_105387C |

Table S4. Performance of different regression models with default parameters that were trained using the reduced feature set after feature selection. Bold text indicates the best-performing model for each score. KNN: K-nearest neighbor regression, MLP: Multi-layer Perceptron regression, GBDT: Gradient Boosting regression, SVR: support vector regression, RBF: Radial basis function, Cubist: Cubist regression, RMSE: root mean squared error; MAE: mean absolute error; MAPE: mean absolute percentage error; r: Pearson correlation, CV: cross validation

| Approach | RMSE | MAE | MAPE | R^2^ | r |
| --- | --- | --- | --- | --- | --- |
| Random Forest* | **6.84** | 2.29 | 0.39 | **0.62** | **0.79** |
| Random Forest | 6.88 | 2.28 | 0.39 | **0.62** | **0.79** |
| KNN | 7.1 | **2.26** | 0.38 | 0.59 | 0.77 |
| MLP | 7.16 | 2.33 | 0.39 | 0.59 | 0.77 |
| XGBoost | 7.18 | 2.29 | 0.39 | 0.58 | 0.77 |
| GBDT | 7.22 | 2.35 | 0.40 | 0.58 | 0.76 |
| SVR (RBF kernel) | 7.51 | 2.29 | **0.37** | 0.55 | 0.77 |
| Cubist | 7.69 | 2.29 | 0.38 | 0.52 | 0.74 |
| Bayesian ridge | 8.8 | 2.61 | 0.44 | 0.38 | 0.61 |
| SVR (linear kernel) | 9.47 | 2.56 | 0.41 | 0.28 | 0.58 |
| AdaBoost | 9.65 | 2.87 | 0.51 | 0.25 | 0.72 |

* with tuned hyperparamters (grid search with 100x 5-fold CV)

**Table S5. Statistical comparison of measured plant dry weights of *A. thaliana* T-DNA insertion lines to the Col-0 wild type using a linear mixed-effect model.** “BH”: Benjamini-Hochberg procedure, * predicted growth reduction at 17 °C, MCC: Matthews correlation coefficient, REML: restricted maximum likelihood, lmer, lme, emmeans, and glht denote the R functions of the packages lme4, nlme, emmeans, and multcomp, respectively.

|  | lmer | | | | lme | | | |
| --- | --- | --- | --- | --- | --- | --- | --- | --- |
| Post-hoc test function | glht | | emmeans | | glht | | emmeans | |
| fixed  intercept | 🗶 | 🗶 | 🗶 | 🗶 | 🗶 | 🗶 | 🗶 | 🗶 |
| random intercept | ✓ | ✓ | ✓ | ✓ | ✓ | ✓ | ✓ | ✓ |
| p-value adjustment | “none” | “BH” | “none” | “BH” | “none” | “BH” | “none” | “BH” |
| Gene ID | P-value | | | | | | | |
| AT3G23580* | 0.001 | 0.004 | 0.004 | 0.025 | 0.001 | 0.004 | 0.001 | 0.009 |
| AT5G08740* | 0.002 | 0.008 | 0.007 | 0.033 | 0.002 | 0.008 | 0.003 | 0.013 |
| AT3G52930* | 0.010 | 0.023 | 0.018 | 0.058 | 0.010 | 0.023 | 0.013 | 0.030 |
| AT1G17290 | 0.008 | 0.023 | 0.022 | 0.058 | 0.008 | 0.023 | 0.011 | 0.030 |
| AT5G38710 | 0.026 | 0.046 | 0.051 | 0.090 | 0.026 | 0.046 | 0.030 | 0.053 |
| AT4G18440 | 0.127 | 0.162 | 0.176 | 0.213 | 0.127 | 0.162 | 0.133 | 0.169 |
| AT2G21940 | 0.052 | 0.082 | 0.088 | 0.136 | 0.052 | 0.082 | 0.058 | 0.090 |
| AT2G02010 | 0.016 | 0.031 | 0.032 | 0.063 | 0.016 | 0.031 | 0.019 | 0.038 |
| AT5G11520 | 0.298 | 0.321 | 0.346 | 0.373 | 0.298 | 0.321 | 0.303 | 0.326 |
| AT2G45290 | 0.578 | 0.578 | 0.613 | 0.613 | 0.578 | 0.578 | 0.580 | 0.580 |
| AT1G22170 | 0.115 | 0.161 | 0.163 | 0.213 | 0.115 | 0.161 | 0.121 | 0.169 |
| AT1G09795 | 0.010 | 0.023 | 0.025 | 0.058 | 0.010 | 0.023 | 0.013 | 0.030 |
| AT2G17630 | 0.139 | 0.163 | 0.182 | 0.213 | 0.139 | 0.163 | 0.145 | 0.169 |
| AT1G62960 | 0.000 | 0.004 | 0.002 | 0.023 | 0.000 | 0.004 | 0.001 | 0.009 |
| Log-likelihood (REML) | 174.649 | | | | | | | |
| MCC | 0.452 | 0.452 | 0.522 | 0.576 | 0.452 | 0.452 | 0.452 | 0.522 |

# SI Dataset legends

**Dataset S1. Sequence-based features for machine learning of protein thermostability optima.** In total, 2839 features were extracted for each sequence. Random forest regression was used within recursive feature elimination with five-fold cross-validation (RFECV) to select the most important features (69).

**Dataset S2. Total protein content at different temperatures.** All measurements in the given publications were done using the Col-0 ecotype. If plant material was harvested at multiple time points during the day, the latest point of the photoperiod was chosen. gFW: gram fresh weight, DAG: days after germination, DAS: days after sowing

**Dataset S3. Experimental measurements of relative growth rates (RGR) of *A. thaliana* Col-0 at different temperatures.** RGR were either directly taken from the publication or calculated from final dry weight measurements, assuming a seed weight of 20 µg (Jako *et al.*, 2001). DAS: days after sowing, DAG: days after germination, DAT: days after transfer, I: irradiance/light intensity

**Dataset S4. Experimental measurements of the net CO_2_ assimilation rate for *A. thaliana* Col-0 at different temperatures.** C_i_: intercellular CO_2_ partial pressure, C_a_: ambient CO_2_ partial pressure, p(O_2_): ambient O_2_ partial pressure, DAG: days after germination, R_H_: relative humidity, I: irradiance, T: temperature, A: net CO_2_ assimilation rate

**Dataset S5. K-medoids clustering of predicted growth responses to metabolite supplementation at different temperatures (irradiance: 150 µmol m^-2^ s^-1^).** For each metabolite, an import reaction (upper bound of 1 mmol/gDW/h) was added before predicting the relative growth rate (RGR) at the different temperatures, respectively. For all simulations, the ratio between NH4+ and NO3- uptake fluxes was fixed to 1:3 (M’rah Helali *et al.*, 2010). Metabolites with increases in relative growth rate below 1% were from the clustering. Cosine distance (1-cosine similarity) was used to compute pairwise distances between the temperature responses. The best K was determined by the maximum of median Silhouette Index values over all K. The explored K ranged from five to 30.

**Dataset S6. K-medoids clustering of predicted growth responses to metabolite supplementation at different temperatures (irradiance: 400 µmol m^-2^ s^-1^).** For each metabolite, an import reaction (upper bound of 1 mmol/gDW/h) was added before predicting the relative growth rate (RGR) at the different temperatures, respectively. For all simulations, the ratio between NH4+ and NO3- uptake fluxes was fixed to 1:3 (M’rah Helali *et al.*, 2010). Metabolites with increases in relative growth rate below 1% were from the clustering. Cosine distance (1-cosine similarity) was used to compute pairwise distances between the temperature responses. The best K was determined by the maximum of median Silhouette Index values over all K. The explored K ranged from five to 30.

**Dataset S7. Responses in relative growth rate (RGR) upon relief of k_cat_ adjustment of each protein individually at different temperatures (irradiance: 400 µmol m^-2^ s^-1^).**

**Dataset S8. Predicted reduction in relative growth rate (RGR) upon single gene knockouts.** To this end, each protein was blocked individually at a simulation temperature of 17 °C. The resulting RGR was divided by the wild-type RGR to obtain the decrease in RGR. To obtain the decrease in RGR, relative to the CO_2_ uptake, each RGR (i.e., mutant and wild-type) were first divided by the respective import flux of CO_2_. The last column contains the IDs of T-DNA insertion lines that were selected for experimental validation.

**Dataset S9. Fresh and dry weights of different Arabidopsis T-DNA lines and the Col-0 wild-type.** Plants with the same batch number were growth together in the same experimental batch. The R stands for replicate.

# References

**Ali AA, Xu C, Rogers A, McDowell NG, Medlyn BE, Fisher RA, Wullschleger SD, Reich PB, Vrugt JA, Bauerle WL, *et al.*** **2015**. Global-scale environmental control of plant photosynthetic capacity. *Ecological Applications* **25**: 2349–2365.

**Arnold A, Nikoloski Z**. **2014**. Bottom-up Metabolic Reconstruction of Arabidopsis and Its Application to Determining the Metabolic Costs of Enzyme Production. *Plant Physiology* **165**: 1380–1391.

**Bateman A, Martin MJ, Orchard S, Magrane M, Agivetova R, Ahmad S, Alpi E, Bowler-Barnett EH, Britto R, Bursteinas B, *et al.*** **2021**. UniProt: the universal protein knowledgebase in 2021. *Nucleic Acids Research* **49**: D480–D489.

**Bernacchi CJ, Portis AR, Nakano H, Von Caemmerer S, Long SP**. **2002**. Temperature response of mesophyll conductance. Implications for the determination of Rubisco enzyme kinetics and for limitations to photosynthesis in vivo. *Plant Physiology* **130**: 1992–1998.

**Binns M, de Atauri P, Vlysidis A, Cascante M, Theodoropoulos C**. **2015**. Sampling with poling-based flux balance analysis: optimal versus sub-optimal flux space analysis of *Actinobacillus succinogenes*. *BMC Bioinformatics* **16**: 49.

**Boyd RA, Cavanagh AP, Kubien DS, Cousins AB**. **2019**. Temperature response of Rubisco kinetics in *Arabidopsis thaliana*: thermal breakpoints and implications for reaction mechanisms. *Journal of Experimental Botany* **70**: 231–242.

**Bunce JA**. **2008**. Acclimation of photosynthesis to temperature in *Arabidopsis thaliana* and *Brassica oleracea*. *Photosynthetica* **46**: 517–524.

**von Caemmerer S**. **2000**. *Biochemical Models of Leaf Photosynthesis*. CSIRO Publishing.

**von Caemmerer S, Evans JR**. **2015**. Temperature responses of mesophyll conductance differ greatly between species. *Plant, Cell and Environment* **38**: 629–637.

**von Caemmerer S, Farquhar G, Berry JA**. **2009**. Biochemical Model of C_3_ Photosynthesis. In: Laisk A, and Nedbal L, Govindjee A, eds. Photosynthesis in silico: Understanding Complexity from Molecules to Ecosystems. Dordrecht: Springer Netherlands, 209–230.

**Chen Z, Liu X, Zhao P, Li C, Wang Y, Li F, Akutsu T, Bain C, Gasser RB, Li J, *et al.*** **2022**. *iFeatureOmega*: an integrative platform for engineering, visualization and analysis of features from molecular sequences, structural and ligand data sets. *Nucleic Acids Research* **50**: W434–W447.

**Cock PJA, Antao T, Chang JT, Chapman BA, Cox CJ, Dalke A, Friedberg I, Hamelryck T, Kauff F, Wilczynski B, *et al.*** **2009**. Biopython: freely available Python tools for computational molecular biology and bioinformatics. *Bioinformatics* **25**: 1422–1423.

**Cousins AB, Pracharoenwattana I, Zhou W, Smith SM, Badger MR**. **2008**. Peroxisomal Malate Dehydrogenase Is Not Essential for Photorespiration in Arabidopsis But Its Absence Causes an Increase in the Stoichiometry of Photorespiratory CO_2_ Release. *Plant Physiology* **148**: 786–795.

**Cousins AB, Walker BJ, Pracharoenwattana I, Smith SM, Badger MR**. **2011**. Peroxisomal hydroxypyruvate reductase is not essential for photorespiration in Arabidopsis but its absence causes an increase in the stoichiometry of photorespiratory CO_2_ release. *Photosynthesis Research* **108**: 91–100.

**Domenzain I, Sánchez B, Anton M, Kerkhoven EJ, Millán-Oropeza A, Henry C, Siewers V, Morrissey JP, Sonnenschein N, Nielsen J**. **2022**. Reconstruction of a catalogue of genome-scale metabolic models with enzymatic constraints using GECKO 2.0. *Nature Communications* **13**: 3766.

**Evans J**. **1987**. The Dependence of Quantum Yield on Wavelength and Growth Irradiance. *Functional Plant Biology* **14**: 69.

**Evans JR**. **1989**. Photosynthesis and nitrogen relationships in leaves of C_3_ plants. *Oecologia* **78**: 9–19.

**Evans J, Terashima I**. **1987**. Effects of Nitrogen Nutrition on Electron Transport Components and Photosynthesis in Spinach. *Functional Plant Biology* **14**: 59.

**Farquhar GD, von Caemmerer S, Berry JA**. **1980**. A biochemical model of photosynthetic CO_2_ assimilation in leaves of C_3_ species. *Planta* **149**: 78–90.

**Farquhar G, Wong S**. **1984**. An Empirical Model of Stomatal Conductance. *Functional Plant Biology* **11**: 191.

**Flexas J, Ortuño MF, Ribas-Carbo M, Diaz-Espejo A, Flórez-Sarasa ID, Medrano H**. **2007**. Mesophyll conductance to CO_2_ in *Arabidopsis thaliana*. *New Phytologist* **175**: 501–511.

**Gandin A, Duffes C, Day DA, Cousins AB**. **2012**. The Absence of Alternative Oxidase AOX1A Results in Altered Response of Photosynthetic Carbon Assimilation to Increasing CO_2_ in *Arabidopsis thaliana*. *Plant and Cell Physiology* **53**: 1627–1637.

**Gerlin L, Cottret L, Escourrou A, Genin S, Baroukh C**. **2022**. A multi-organ metabolic model of tomato predicts plant responses to nutritional and genetic perturbations. *Plant Physiology* **188**: 1709–1723.

**Heckwolf M, Pater D, Hanson DT, Kaldenhoff R**. **2011**. The *Arabidopsis thaliana* aquaporin AtPIP1;2 is a physiologically relevant CO_2_ transport facilitator. *Plant Journal* **67**: 795–804.

**Hobbs JK, Jiao W, Easter AD, Parker EJ, Schipper LA, Arcus VL**. **2013**. Change in heat capacity for enzyme catalysis determines temperature dependence of enzyme catalyzed rates. *ACS Chemical Biology* **8**: 2388–2393.

**Hummel I, Pantin F, Sulpice R, Piques M, Rolland G, Dauzat M, Christophe A, Pervent M, Bouteillé M, Stitt M, *et al.*** **2010**. Arabidopsis Plants Acclimate to Water Deficit at Low Cost through Changes of Carbon Usage: An Integrated Perspective Using Growth, Metabolite, Enzyme, and Gene Expression Analysis. *Plant Physiology* **154**: 357–372.

**Jako C, Kumar A, Wei Y, Zou J, Barton DL, Giblin EM, Covello PS, Taylor DC**. **2001**. Seed-Specific Over-Expression of an Arabidopsis cDNA Encoding a Diacylglycerol Acyltransferase Enhances Seed Oil Content and Seed Weight. *Plant Physiology* **126**: 861–874.

**Jarząb A, Kurzawa N, Hopf T, Moerch M, Zecha J, Leijten N, Bian Y, Musiol E, Maschberger M, Stoehr G, *et al.*** **2020**. Meltome atlas—thermal proteome stability across the tree of life. *Nature Methods* **17**: 495–503.

**Leuning R**. **2002**. Temperature dependence of two parameters in a photosynthesis model. *Plant, Cell & Environment* **25**: 1205–1210.

**Lewis NE, Hixson KK, Conrad TM, Lerman JA, Charusanti P, Polpitiya AD, Adkins JN, Schramm G, Purvine SO, Lopez-Ferrer D, *et al.*** **2010**. Omic data from evolved *E. coli* are consistent with computed optimal growth from genome-scale models. *Molecular Systems Biology* **6**: 390.

**Luo H, Carriquí M, Nadal M, Han T, Werner C, Huang J, Zhang J, Yu Z, Li F, Fang X, *et al.*** **2021**. Explicit expression of mesophyll conductance in the traditional leaf photosynthesis–transpiration coupled model and its physiological significances. *bioRxiv*: 2021.09.22.461327.

**M’rah Helali S, Nebli H, Kaddour R, Mahmoudi H, Lachaâl M, Ouerghi Z**. **2010**. Influence of nitrate—ammonium ratio on growth and nutrition of *Arabidopsis thaliana*. *Plant and Soil* **336**: 65–74.

**Mahadevan R, Schilling CH**. **2003**. The effects of alternate optimal solutions in constraint-based genome-scale metabolic models. *Metabolic Engineering* **5**: 264–276.

**Morales A, Kaiser E, Yin X, Harbinson J, Molenaar J, Driever SM, Struik PC**. **2018**. Dynamic modelling of limitations on improving leaf CO_2_ assimilation under fluctuating irradiance. *Plant Cell and Environment* **41**: 589–604.

**Murray FW**. **1967**. On the Computation of Saturation Vapor Pressure. *Journal of Applied Meteorology* **6**: 203–204.

**Niinemets Ü, Díaz-Espejo A, Flexas J, Galmés J, Warren CR**. **2009**. Importance of mesophyll diffusion conductance in estimation of plant photosynthesis in the field. *Journal of Experimental Botany* **60**: 2271–2282.

**Pons TL**. **2012**. Interaction of temperature and irradiance effects on photosynthetic acclimation in two accessions of *Arabidopsis thaliana*. *Photosynthesis Research* **113**: 207–219.

**Pyl E-T, Piques M, Ivakov A, Schulze W, Ishihara H, Stitt M, Sulpice R**. **2012**. Metabolism and Growth in Arabidopsis Depend on the Daytime Temperature but Are Temperature-Compensated against Cool Nights. *The Plant Cell* **24**: 2443–2469.

**Sánchez BJ, Zhang C, Nilsson A, Lahtvee P, Kerkhoven EJ, Nielsen J**. **2017**. Improving the phenotype predictions of a yeast genome‐scale metabolic model by incorporating enzymatic constraints. *Molecular Systems Biology* **13**: 935.

**Shaw R, Cheung CYM**. **2018**. A Dynamic Multi-Tissue Flux Balance Model Captures Carbon and Nitrogen Metabolism and Optimal Resource Partitioning During Arabidopsis Growth. *Frontiers in Plant Science* **9**: 884.

**Shaw R, Cheung CYM**. **2021**. Integration of crop growth model and constraint-based metabolic model predicts metabolic changes over rice plant development under water-limited stress. *in silico Plants* **3**: 20.

**Simas Coutinho Barbosa J, Schroeder WL, Suthers PF, Jawdy SS, Chen J-G, Muchero W, Maranas CD**. **2024**. A Multi-Tissue Genome-Scale Model of Populus trichocarpa Elucidates Overexpression Targets for Improving Drought Tolerance. *in silico Plants*.

**Walker B, Ariza LS, Kaines S, Badger MR, Cousins AB**. **2013**. Temperature response of *in vivo* Rubisco kinetics and mesophyll conductance in *Arabidopsis thaliana*: Comparisons to *Nicotiana tabacum*. *Plant, Cell and Environment* **36**: 2108–2119.

**Walker BJ, Cousins AB**. **2013**. Influence of temperature on measurements of the CO_2_ compensation point: Differences between the Laisk and O_2_-exchange methods. *Journal of Experimental Botany* **64**: 1893–1905.

**Weston DJ, Karve AA, Gunter LE, Jawdy SS, Yang X, Allen SM, Wullschleger SD**. **2011**. Comparative physiology and transcriptional networks underlying the heat shock response in *Populus trichocarpa*, *Arabidopsis thaliana* and *Glycine max*. *Plant, Cell and Environment* **34**: 1488–1506.

**Yang Y, Zhao J, Zeng L, Vihinen M**. **2022**. ProTstab2 for Prediction of Protein Thermal Stabilities. *International Journal of Molecular Sciences* **23**: 10798.

**Yin X, Goudriaan J, Lantinga EA, Vos J, Spiertz HJ**. **2003**. A flexible sigmoid function of determinate growth. *Annals of Botany* **91**: 361–371.
